# Tuning heterologous glucan biosynthesis in yeast to understand and exploit plant starch diversity

**DOI:** 10.1186/s12915-022-01408-x

**Published:** 2022-09-24

**Authors:** Barbara Pfister, Jessica M. Shields, Tobias Kockmann, Jonas Grossmann, Melanie R. Abt, Martha Stadler, Samuel C. Zeeman

**Affiliations:** 1 Institute of Molecular Plant Biology, ETH Zurich, 8092 Zurich, Switzerland; 2 Current address: College of Medicine and Health, University of Exeter, Exeter, UK; 3 Functional Genomics Center Zurich, ETH Zurich, 8057 Zurich, Switzerland; 4 SIB Swiss Institute of Bioinformatics, 1015 Lausanne, Switzerland

**Keywords:** Heterologous expression in yeast, YFP reporter, Amylopectin structure, Starch biosynthesis, Parallel reaction monitoring, Proteomics, *Arabidopsis thaliana*

## Abstract

**Background:**

Starch, a vital plant-derived polysaccharide comprised of branched glucans, is essential in nutrition and many industrial applications. Starch is often modified post-extraction to alter its structure and enhance its functionality. Targeted metabolic engineering of crops to produce valuable and versatile starches requires knowledge of the relationships between starch biosynthesis, structure, and properties, but systematic studies to obtain this knowledge are difficult to conduct in plants. Here we used *Saccharomyces cerevisiae* as a testbed to dissect the functions of plant starch biosynthetic enzymes and create diverse starch-like polymers.

**Results:**

We explored yeast promoters and terminators to tune the expression levels of the starch-biosynthesis machinery from *Arabidopsis thaliana*. We systematically modulated the expression of each starch synthase (SS) together with a branching enzyme (BE) in yeast. Protein quantification by parallel reaction monitoring (targeted proteomics) revealed unexpected effects of glucan biosynthesis on protein abundances but showed that the anticipated broad range of SS/BE enzyme ratios was maintained during the biosynthetic process. The different SS/BE ratios clearly influenced glucan structure and solubility: The higher the SS/BE ratio, the longer the glucan chains and the more glucans were partitioned into the insoluble fraction. This effect was irrespective of the SS isoform, demonstrating that the elongation/branching ratio controls glucan properties separate from enzyme specificity.

**Conclusions:**

Our results provide a quantitative framework for the in silico design of improved starch biosynthetic processes in plants. Our study also exemplifies a workflow for the rational tuning of a complex pathway in yeast, starting from the selection and evaluation of expression modules to multi-gene assembly and targeted protein monitoring during the biosynthetic process.

**Supplementary Information:**

The online version contains supplementary material available at 10.1186/s12915-022-01408-x.

## Background

Targeted engineering of a biochemical network requires understanding the pathway’s response to perturbation, and thus a detailed understanding of the involved components, including enzymes’ kinetic rates, concentrations, and interdependences. Despite the CRISPR/Cas9 revolution, targeted genome manipulation to address these issues is still difficult and time consuming to achieve in most plants and other organisms. *Saccharomyces cerevisiae* (referred to as yeast hereafter) offers a powerful heterologous system to study the function of non-yeast proteins. Because of the ease of genetic manipulation and maintenance in the lab, yeast is frequently used to test protein-protein interactions, protein function by complementation, or the impact of mutations on protein activity [1–3]. In addition, yeast often serves as a microbial cell factory: reconstituting complete metabolic pathways in a heterologous system does not only allow the large-scale production of valuable molecules but also provides a deeper understanding of the pathway as a whole [4–6].

Starch biosynthesis illustrates the need for rational engineering in planta and the prospects of using cross-system knowledge when doing so. Starch accumulates as water-insoluble granules in plants and consists of two distinct α-glucose polymers. The first is amylopectin, a large α-1,4-linked glucan with moderately frequent, clustered α-1,6-branch points, resulting in a tree-like architecture. Amylopectin forms a semi-crystalline matrix, rendering starch insoluble. The second polymer is amylose, which is smaller, lightly branched and occupies the spaces within the amylopectin matrix [7]. The structural features of starch confer valuable physicochemical properties, in particular the ability to form viscous solutions after heating in water, and gels upon subsequent cooling. Such properties render starch a bulk commodity for both food and non-food industries, where it serves as an emulsifier, thickener, adhesive, etc. [8]. Plant starches from different botanical sources vary in their physicochemical properties owing to subtle differences in starch composition, structure, and granule morphology, but the diversity of starches presently available does not satisfy industrial needs [9]. Consequently, waste-generating and/or costly chemical or physical modifications are required to improve starch functionality post-extraction. Targeted genetic improvement of starch crops could serve to diversify starches in planta and would be a more sustainable approach to providing this important industrial feedstock [10]. However, the difficulties in creating plant lines with defined genetic modifications have hindered systematic analyses to establish the relationships between the enzymatic processes, starch structure, and starch functionality. Using yeast as an orthologous host to study these relationships promises to greatly enhance our basic understanding and guide starch crop improvement and diversification, fulfilling longstanding agricultural biotechnology goals.

The nature of the starch biosynthetic pathway presents a major challenge to targeted starch improvement. Amylopectin is synthesized by the simultaneous and concerted action of starch synthases (SSs, which elongate α-1,4-linked chains), branching enzymes (BEs, which transfer part of a chain to create an α-1,6-linked branch), and debranching enzymes of the isoamylase (ISA) class (which selectively remove some branches). Amylose is synthesized within the amylopectin matrix by granule-bound starch synthase (GBSS) [11]. During amylopectin synthesis, at least three starch synthases (SS1 to SS3) elongate the glucan chains using ADPglucose (adenosine 5’-diphosphate-glucose) as glucose donor. In a simplified model, the branches derived from BE activity are first elongated by SS1, then SS2 elongates them further, and SS3 synthesizes the long chains that span layers of the amylopectin tree [12]. SS4 is also an active SS, but its primary function appears to lie in the initiation of starch granules [13, 14]. Branch points are introduced by one or two classes of branching enzymes (BEs) [15]. There are two active BEs in Arabidopsis—BE2 and BE3—which belong to the same class and seem largely redundant [16]. Last, some of the branches created by BEs are hydrolyzed again by a trimming isoamylase (ISA) activity, which in Arabidopsis is a heteromultimeric enzyme composed of ISA1 and ISA2 subunits. This trimming is thought to aid the crystallization of the glucan [17]. In general, the enzymes of starch biosynthesis are highly conserved among plants. However, their relative contribution to the overall process differs between different species, partly explaining the structural and physicochemical diversity in starches from various crops [18].

The current model of starch biosynthesis is primarily based on the analyses of plant lines that show a complete loss or drastic reduction in expression of one or few enzymes. However, interpretation of such phenotypic data can be complicated by multiple issues, as enzymes often show partial or complete functional overlap with others [16, 19] or display functional dependency [20]. Also other pleiotropic effects may contribute to the phenotype, for instance by altering complex formation [21] or if enzymes not normally participating in starch synthesis further modify aberrant glucans [16, 17]. While numerous mutants in different species have added to our current understanding, there is much less information from overexpression or moderate knockdown plant lines. Nevertheless, there are indications that enzyme ratios are also important in determining glucan structure [22, 23], a concept consistent with the enzymes acting in tandem and interdependently. In vitro analyses are another source of valuable information on enzyme kinetics and substrate preferences but are often limited to particular substrates, which may only partly reflect the range of native substrates in vivo, including the growing surface of the starch granule [24].

The difficulties in correctly delineating the exact function of individual proteins on the overall starch biosynthesis process prompted us to develop a heterologous system in which we could study protein function in a defined, in vivo environment. We showed that the synthesis of starch-like glucans can be reconstituted in yeast using the genes from Arabidopsis [25]. Heterologous expression of *SS1* to *SS4*, *BE2*, *BE3*, *ISA1*, and *ISA2*, together with a bacterial ADPglucose pyrophosphorylase (AGPase) gene for substrate provision, yielded insoluble granules with a semi-crystalline structure similar to that of plant amylopectin. This demonstrated that yeast can serve as a simple and clean platform to facilitate studying the multi-enzyme process of starch biosynthesis. Here, we redesigned our yeast system in order to modulate both starch-biosynthesis gene content and their relative expression levels. Combining individual SSs with a BE at defined ratios allows to assess the contribution of each enzyme in a quantitative way, thereby providing a clear-cut view of their impact on glucan structure.

## Results

### Characterization of promoters and terminators for expression modulation

In our original yeast system to analyze plant starch biosynthesis [25], all transgenes were driven by the strong galactose-inducible promoters *pGAL1* or *pGAL10*. This allowed the induction of gene expression (and consequently glucan synthesis) by switching from glucose to galactose as a carbohydrate source but with no modulation of expression strength. We redesigned the system using native yeast promoters and terminators of different strengths to control the expression levels of the *SS* and *BE* genes from *Arabidopsis thaliana* (Arabidopsis). We reasoned that the use of *pGAL1* for the expression of the bacterial AGPase glgC-TM (to supply SSs with their ADPglucose substrate) would suffice to induce glucan biosynthesis through the glucose-galactose switch. Indeed, strains expressing the complete set of amylopectin-biosynthetic enzymes but containing no *glgC-TM* gene did not produce any glucans, demonstrating that the flux into starch biosynthesis is tightly controlled by this locus (Additional file 1: Fig. S1A [13, 20, 25–30]).

**Fig. 1:**
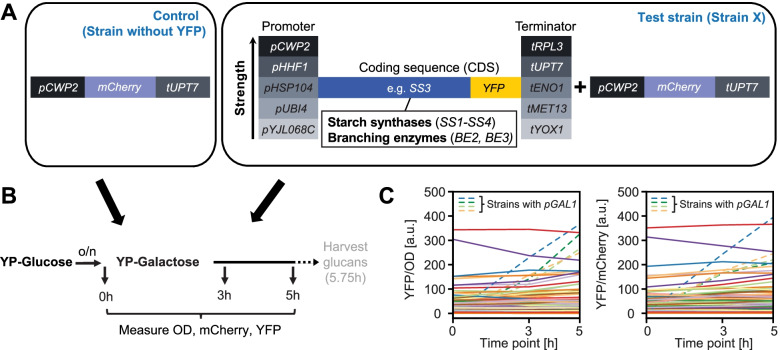
Experimental setup to characterize promoter and terminator activities. **A** Test strains (right) contain a CDS-YFP reporter controlled by promoters and terminators of different strengths, plus an mCherry reporter as internal control. Control strains (left) with only the mCherry reporter serve to correct for background when monitoring YFP. **B** Yeast strains cultivated in rich medium with galactose were assessed for optical cell density (OD), and mCherry and YFP fluorescence at the indicated time points using a plate reader. The same growth regime (harvest after 5.75h) was used later for strains designed for glucan production. **C** YFP fluorescence of test strains normalized to OD (left panel) or to mCherry (right panel). Strains with *pGAL1*-driven YFP reporters are indicated by dashed lines

To modulate gene expression of the *SS* and *BE* genes in yeast, we mined large-scale reporter gene datasets [26, 27] and selected five yeast promoters that spanned an 18-fold range of activity and five terminators that spanned a 13-fold range of activity (Fig. 1A). The selected promoters showed little or no change in activity when galactose was used as a carbohydrate source instead of glucose [26]. The *pCUP1* promoter, which is barely active under basal conditions [31, 32], and *pGAL1* were included as controls. We employed a YFP reporter system in haploid CEN.PK113-11C *S. cerevisiae* to systematically test the activities of the selected promoter/terminator modules fused to the coding sequence (CDS) of a SS or BE (without their predicted chloroplast transit peptides) and with a C-terminal YFP tag in our experimental setup (Fig. 1A). In total, we created 60 yeast strains that contained a single, stably integrated YFP reporter construct and a separate mCherry reporter as an internal control.

We measured YFP fluorescence at multiple time points and normalized it to either optical density (OD) (YFP/OD) or mCherry fluorescence (YFP/mCherry; Fig. 1). The two normalization methods gave highly comparable values (R^2^ ≥ 0.95, Fig. 1C, Additional file 1: Fig. S2A), showing that either corrects for changes in cellular density. We primarily used YFP/mCherry fluorescence for data analysis because of lower technical error (see “Methods”) but include YFP/OD data for comparison where relevant. High reproducibility (Additional file 1: Fig. S2) allowed us to compile all measurements into a single data set.

Normalized YFP fluorescence of the different strains showed a wide spread, illustrating the range of expression levels that were successfully obtained from using different promoters, CDS, and terminators (Fig. 1C). Comparing the normalized YFP fluorescence at the different time points showed that the expression per unit biomass was remarkably stable during the time course (Fig. 1C; Additional file 1: Figs. S3, S4). Thus, switching from glucose to galactose in the medium had no or little effect on the activities of our promoters. Western blots showed that measured YFP signals primarily derived from the full-length, soluble CDS-YFP fusion proteins (Additional file 1: Fig. S5).

**Fig. 2:**
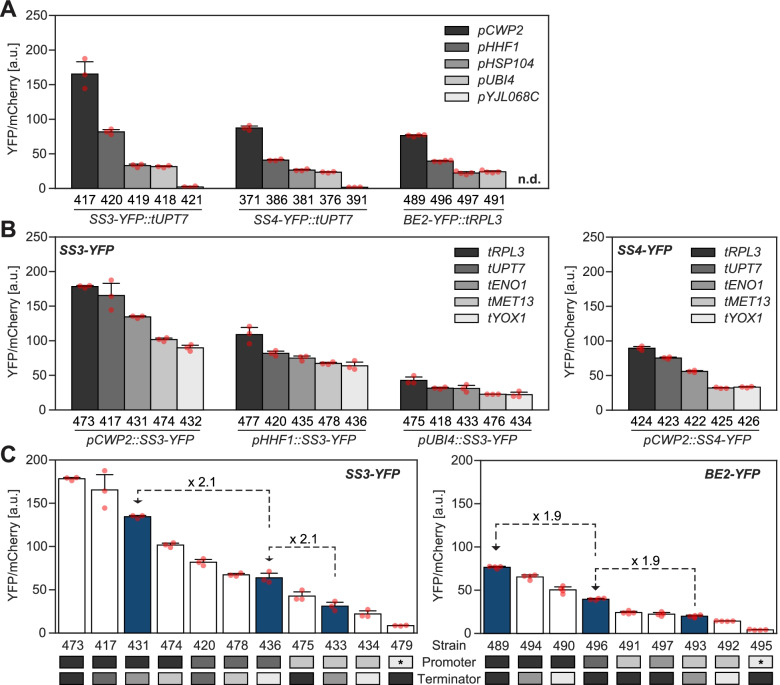
Promoters and terminators influence heterologous gene expression independently and irrespective of the CDS. YFP fluorescence normalized to mCherry at time point 3 h (means ± S.D.; *n* = 3 replicate cultures, except for all strains with *BE2-YFP*, where *n* = 4), with red points depicting the individual replicates (see Additional file 2 for numerical data). Strain numbers are given below the bars. Data was partly replotted in the individual panels. **A** Effect of the different promoters exemplified on three CDS-YFP fusions. Within each CDS group, only the promoter varies. n.d., not determined. **B** Effect of the different terminators exemplified on SS3-YFP (left) and SS4-YFP (right). Within each strain group, only the terminator varies. **C** Range of expression of SS3-YFP (left) and BE2-YFP (right) obtained by varying the promoters and terminators. Promoter/terminator identities are indicated by the shades shown in panels **A** and **B**, respectively (the promoter marked with an asterisk is *pCUP1*). Dark blue bars correspond to the promoter/terminator combinations that were later used to express untagged SS3 and BE2 at strong, medium, or weak levels, with numbers showing the observed fold changes of fluorescence

**Fig. 3:**
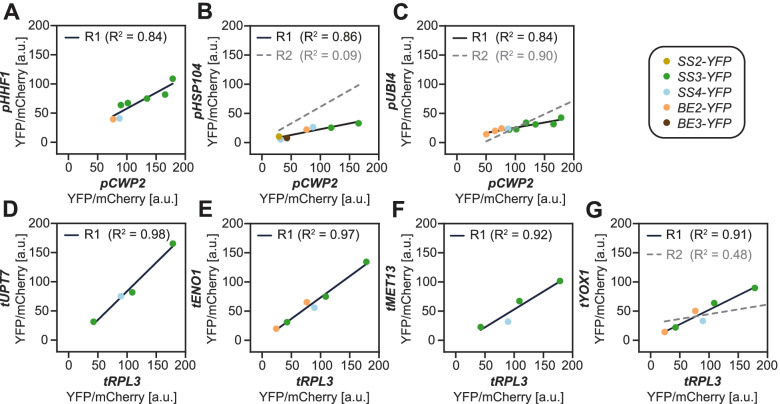
Influence of the promoters and terminators on expression levels. Mean YFP fluorescence normalized to mCherry (time point 3 h) of strains that are isogenic except for the promoter (**A–C**) and terminator (**D–G**) of the CDS-YFP constructs, respectively. The mean YFP/mCherry fluorescence of one strain is plotted on the *x*-axis and that of the other on the *y*-axis. The respective YFP protein fusion is indicated by color. Only promoter/terminator comparisons for which at least four comparisons were available are shown. Solid black lines show linear regressions, in which single outliers from SS1-YFP (**C**, **G**) or SS2-YFP (**B**) fusions were excluded; the corresponding data points are beyond the displayed areas. Dashed gray lines (R2) show regressions that include the outlier samples. Details and statistics are given in Additional file 1: Table S2, and numerical data is given in Additional file 2. R^2^, coefficient of determination

We next examined our data for distinct effects of the promoters and terminators, focusing on the 3-h time point. When comparing strains that differ only by the promoter that drives the YFP reporter, we typically observed the following sequence of promoter activities: *pCWP2* > *pHHF1 > pHSP104* ~ *pUBI4 > pYJL068C* (Fig. 2A), as anticipated [26]. The relative promoter activities were largely independent of the CDS, terminator, or yeast genomic locus (Fig. 3A–C). However, for SS1- and SS2-YFP constructs, we occasionally observed exceptions, rendering the assumption of universal promoter activities to predict the expression of all our constructs difficult (Fig. 3A–C).

The terminators also influenced gene expression largely independently of the promoter and CDS (Figs. 2B and 3D–G). The sequence in terminator strengths matched that reported before by Yamanishi et al. [27]. Yet, while they reported a ca. 13-fold difference between the strongest (*tRPL3*) and weakest terminator (*tYOX1*) from our set, we observed only a 2-fold difference (Fig. 3G). Similar to the promoters, there was an outlier from SS1-YFP that diverged from the other CDS for reasons unknown (Fig. 3G). Despite the fact that the range in terminator strengths was less than previously reported, the combined use of promoters and terminators allowed us to create a 20-fold range of expression levels with small increments, as exemplified on SS3-YFP and BE2-YFP (Fig. 2C). Thus, heterologous gene expression in yeast can be stringently controlled by using just a few modules, though careful upfront testing is required.

### Generation of yeast strains expressing a single SS and BE2

For the analysis of glucans produced by the Arabidopsis enzymes, we expressed the proteins in a background purged of any interfering endogenous glucan-metabolic activity. We first created the precursor strain 362.1 carrying deletions in glycogenins (*GLG1* and *GLG2*), glycogen synthases (*GSY1* and *GSY2*), and glycogen branching enzyme (*GLC3*). As a precaution we also eliminated maltase activity from this background by CRISPR/Cas9 gene editing, even though maltases primarily hydrolyze maltose and hardly act on long malto-oligosaccharides or glucans [33, 34], leading to strain 504 (Additional file 1: Figs. S6, S7, Additional file 3). Glycogen debranching enzyme (*GDB1*), and glycogen phosphorylase (*GPH1*) were subsequently deleted during the integration of the Arabidopsis genes or of an empty vector. Strain 504 further possessed the galactose-inducible *glgC-TM* gene for providing ADPglucose and the mCherry reporter for biomass quantification.

We employed the promoter/terminator modules to express enzyme pairs consisting of a single SS and BE2 (in their untagged forms) at different ratios in strain 504. BE2 was used rather than BE3 because we had more BE2-YFP reporter data available. Western blots using polyclonal antibodies specific for the SS proteins showed that the untagged SSs migrated similarly to the endogenous Arabidopsis counterparts present in chloroplasts (Additional file 1: Fig. S8). Antibodies were raised against BE2 but unfortunately did not recognize their target in yeast or in plants (not shown). For SSs and for BE2, we selected promoters and terminators in order to obtain weak, medium, or strong expression based on our YFP reporter data, approximately doubling the expression with each step (see Fig. 2C for examples of SS3 and BE2). The resulting expression constructs were combined to achieve a wide range of SS/BE2 ratios in a set of six strains for each of the four SSs (24 strains in total; Additional file 1: Table S1). The resulting molar SS/BE2 stoichiometries were estimated comparing the fluorescence values of the SS- and BE2-YFP fusions, assuming that the signals of the YFP-tagged fusion proteins are a proxy for the abundances of the untagged versions (Additional file 1: Table S1). Activity gels suggested that the levels of SS and BE2 activity indeed vary between the strains and also reconfirmed that they retain enzymatic activity when expressed in yeast (Additional file 1: Fig. S9). However, we emphasize that activity gels are semi-quantitative, necessitating validation of protein levels, as described below.

### Yeasts with low branching activity produce insoluble glucans

To assess glucan synthesis, we grew the six strains for each SS-BE2 combination as in Fig. 1B and harvested the cells after 5.75h in YP-galactose. We used the mCherry reporter and OD as a proxy for wet weights (WW), allowing us to down-size cultures (Additional file 1: Fig. S10). As expected, no glucans were observed when cells were grown in medium with glucose where glgC-TM expression is repressed (Additional file 1: Figs. S1B and S11).

**Fig. 4:**
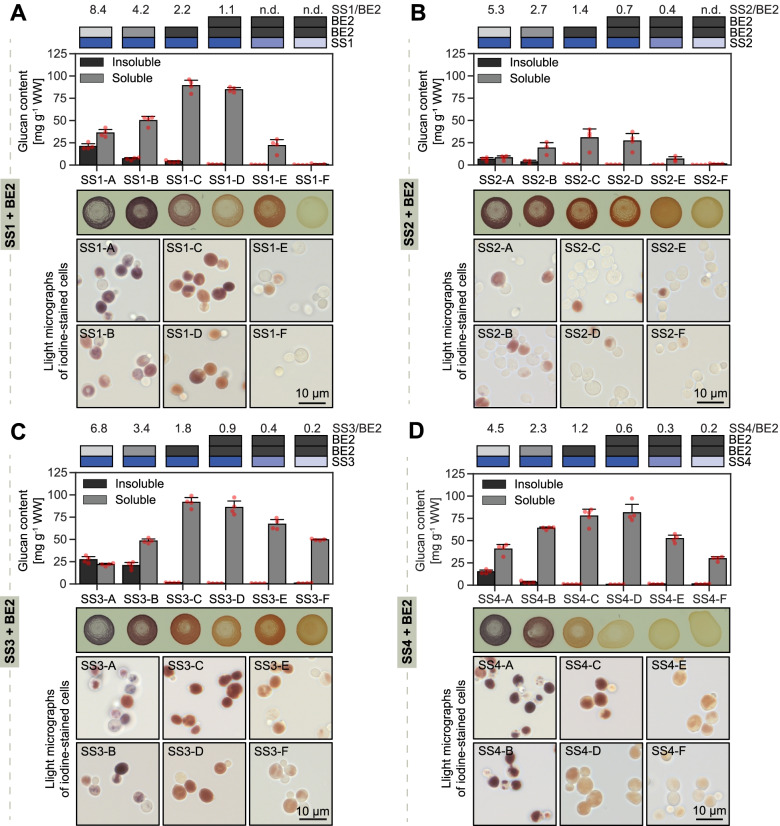
Glucan biosynthesis in yeast strains expressing a single starch synthase (SS) and BE2. Upper panels: Glucan content of yeasts expressing the indicated SS and BE2 grown as in Fig. 1B (means ± S.D. from 4 replicate cultures, except for SS2-B [*n* = 3], SS2-E [*n* = 3] and SS4-C [*n* = 5]). Red points depict the individual replicates. The anticipated expression strengths and the estimated molar ratio of the SS to BE2 protein molecules are given on top. Numerical data are provided in Additional file 4, sheet 1. N.d., not determined; WW, wet weight. Middle panels: Iodine staining of cell patches grown for 24 h on galactose-containing plates. Glucose-grown controls are presented in Additional file 1: Fig. S11. Lower panels: Iodine staining of the cell cultures used for glucan extraction. The size bar applies to all images

Strains SS1-A to SS1-E, which expressed SS1 and BE2 at various ratios, all accumulated high amounts of glucans, but the partitioning of the glucan between soluble and insoluble fractions varied considerably (Fig. 4A). In strain SS1-A, where the SS1/BE2 ratio was anticipated to be highest, 37 ± 2 % (mean ± S.D.) of the glucans were insoluble, despite the absence of any trimming ISA activity. With increasing BE2 activity (strains SS1-B to SS1-D), most to all glucans became soluble. Glucan contents decreased from SS1-D to SS1-F, probably because the decreasing SS1 protein abundance limits glucose-polymerizing activity (note that strain SS1-F employed a very weak *pCUP1*-driven *SS1* construct).

The differences in accumulation of insoluble and soluble glucans of the yeast strains were accompanied by dissimilar staining of cells with iodine, both when grown on plates and when grown in liquid culture (Fig. 4A, middle and lower panels). Iodine is a qualitative glucan stain; blue staining is typical of glucans with few branches and single-helical structures (as in amylose), red-brown staining indicates double-helical structures (as in amylopectin), and orange-brown staining indicates many short branches and relatively few secondary structures (as in glycogen) [35, 36]. Accordingly, the rather bluish staining of SS1-A suggests that its glucans carry relatively few branches, which was expected considering the low BE2 activity. However, the increasingly reddish to orange staining of strains SS1-B to SS1-D indicates the accumulation of increasingly branched glucans, again in agreement with the expected increasing BE2 activity relative to SS1 activity. Together, these data suggest that SS1/BE2 ratio markedly influences the kind of glucan produced, with low branching activity favoring the synthesis of insoluble glucans with fewer branches.

The patterns of glucan partitioning and iodine staining in relation to synthase and branching activity were similar in strains expressing SS2, SS3, and SS4 as sole SS together with BE2 (Fig. 4B–D). However, in case of the strains containing SS2, we consistently measured few total glucans (Fig. 4B). This was unexpected since previous yeast strains expressing SS2 together with BEs produced glucan levels as high as for the other SSs [25]. However, in these previous strains, SS2 had been driven by the *pGAL1* promoter, which appears to yield several-fold higher SS2 levels than the strongest constructs employed here (judged by targeted proteomics comparing SS2 abundance in the previous strain 28 and in the current set, described below). In addition, iodine staining of SS2-containing strains grown in liquid culture revealed a heterogeneous cell population with only a few stained cells (Fig. 4B, lower panel). This stochasticity is reminiscent of glycogen biosynthesis in yeast mutants lacking glycogenins, which provide the glucan primers for subsequent elaboration by the endogenous glycogen synthase(s) and a BE [37]. Thus, here it may be indicative of a poor ability of SS2 to initiate glucans, which may only become apparent if SS2 abundance is low. Since both glycogenin genes have been deleted in our strains, it is unclear what serves as a primer within the yeast cells.

### Relative BE and SS activities markedly influence glucan structure

Iodine staining of cells does not allow differentiation between the staining of soluble and insoluble glucans produced within the same cell. We therefore acquired iodine absorption spectra of the isolated soluble and insoluble yeast glucans and determined their wavelengths of maximum light absorption (*λ*_max_ values). To further assess the glucan structures, we determined the relative abundances of the chains that make up the glucose polymers after linearization by enzymatic debranching (chain length distributions or CLDs).

**Fig. 5:**
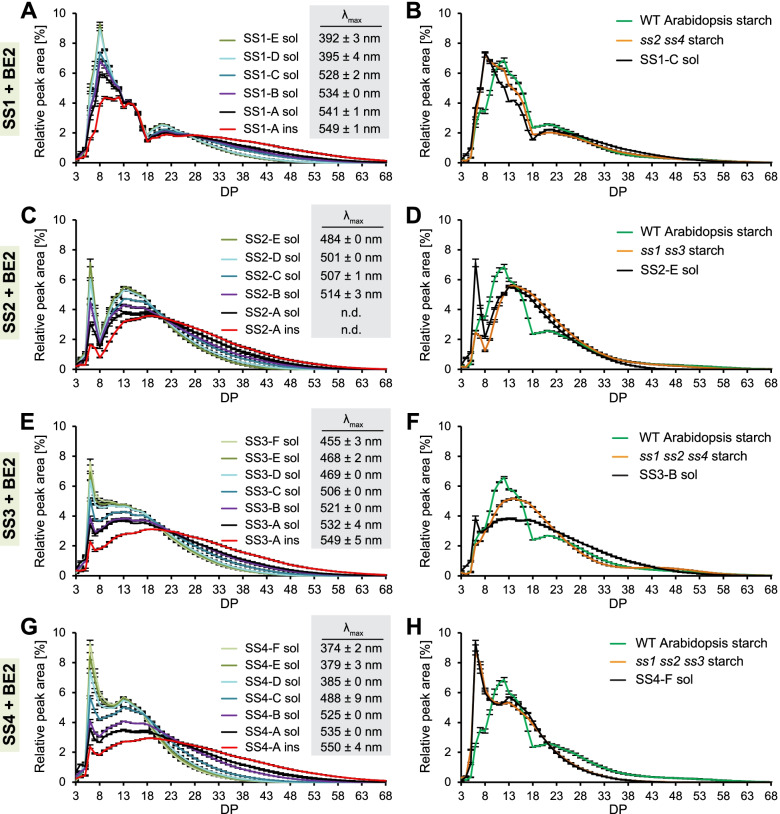
Chain-length distributions (CLDs) of glucans and their wavelengths of maximum absorption after iodine complexion (*λ*_max;_ gray boxes). CLDs from plant starches are included for comparison. *λ*_max_ values were not acquired from these mutant plant starches as their amylose component would have masked the absorption spectra of the amylopectins. Relative peak areas are means ± S.E.M., and values in gray boxes indicate the *λ*_max_ of the glucan after iodine complexion (means ± S.D.). Data was partly replotted in the individual panels. Numerical data are provided in Additional file 5. Sol, soluble glucans; ins, insoluble glucans; DP, degree of polymerization. Sample sizes for CLDs are 4 yeast replicate cultures or plant rosettes, except for wild-type (WT) Arabidopsis (*n =* 2 in panel **F** and *n* = 3 in all other panels), SS1-C sol (*n =* 3), SS2-A ins (*n =* 3), SS3-B sol (*n =* 2), and SS4-A sol (*n =* 2). Sample sizes for *λ*_max_ values are 4 yeast replicate cultures or plant rosettes, except for SS1-E sol, SS2-B sol, SS2-C sol, SS2-D sol, SS2-E sol, SS3-A sol, and SS4-A sol, where *n =* 3

The CLDs of soluble glucans from the SS1-BE2 yeast strain set all showed similar features: they had a bimodal distribution with a peak at chains with a degree of polymerization (DP) of 8 and one at DP21 (Fig. 5A). The dominance of DP8 clearly increased from strains SS1-A to SS1-E, in which the branching to synthase activity was expected to increase, at the expense of long chains > DP26. The abundance of shorter chains was accompanied by progressively lower *λ*_max_ values of the glucans after complexion with iodine (numbers inset in Fig. 5A), suggesting fewer secondary structures. Soluble glucans from SS1-F had a *λ*_max_ that was even lower than that of glycogen (typically ~450 nm) [36, 38]. By contrast, the *λ*_max_ of the insoluble glucans from SS1-A was similar to that of plant amylopectin (*λ*_max_ of 539 nm and 549 nm for Arabidopsis and potato amylopectin, respectively), indicating the presence of secondary structures. The chain lengths of insoluble glucans from SS1-A were also longer compared with their soluble counterparts (Fig. 5A).

The CLD of wild-type Arabidopsis leaf starch was distinct from that of yeast glucans made by SS1 and BE2 (Fig. 5B). This was expected because wild-type leaves also contain BE3, ISA1-ISA2, and all the other SS isoforms, of which particularly SS1 and SS2 are known to influence the CLDs [19, 20, 39, 40]. Structural comparison with the corresponding Arabidopsis *ss* triple mutants was not feasible, since plants deficient in both SS3 and SS4 are incapable of initiating glucan synthesis and are thus near starchless [41]. However, the CLD of starch from *ss2 ss4* mutants, which only possess SS1 and SS3 for amylopectin chain elongation, was highly similar to that of glucans from the yeast strains expressing SS1, as chains of DP8 were most abundant (Fig. 5B). We note that the yeast glucans from SS1-C, whose CLD was most similar to that of *ss2 ss4* starch, were soluble, while plant starch is insoluble. This was also the case for the comparisons of the other yeast strains sets (described below) and indicates that the yeast glucans differ from plant starch in aspects that are not apparent in CLDs but that also control the partitioning of glucans into the soluble *vs.* insoluble fraction, such as branching pattern or particle size.

Strains containing BE2 together with SS2, SS3, or SS4 (Fig. 5C–H) showed the same trend towards producing glucans with more short chains and lower *λ*_max_ values when branching activity increased. However, instead of a peak at DP8, these glucans had a marked peak at DP6. As DP6 constitute the shortest chains that are efficiently transferred by plant BEs [42, 43], their accumulation probably reflects the limited ability of SS2, SS3, and SS4 to use DP6 as a substrate. The CLDs from starches from corresponding Arabidopsis mutants again resembled those from the yeast glucans. However, in case of SS3, the peak at DP14 was considerably broader in the yeast glucans than in *ss1 ss2 ss4* starch for unknown reasons.

Taken together, these data reconfirmed that the SSs retain their distinct specificities for chain elongation when expressed in yeast. Importantly, our results show unambiguously that the SS/BE2 ratio strongly influences the resultant glucan structure: more branching activity favors the accumulation of short chains and glycogen-like iodine binding, indicative of water-soluble glucans with no or few secondary structures. Conversely, less branching activity favors the accumulation of longer chains and, interestingly, the formation of insoluble glucans despite the absence of ISA1-ISA2 debranching activity.

### Determination of protein abundances by targeted proteomics

To test whether the modulation of protein abundances, anticipated from the YFP reporter data, holds true for the untagged proteins actively engaged in glucan synthesis in yeast, we developed parallel reaction monitoring (PRM) proteomics assays for the relative quantification of each of the heterologously expressed proteins. We selected multiple surrogate peptides for each protein and characterized the assays with respect to their dynamic range. We then assessed all strains created for glucan production at the 3-h time point, when the rate of glucan synthesis was expected to be high.

**Fig. 6:**
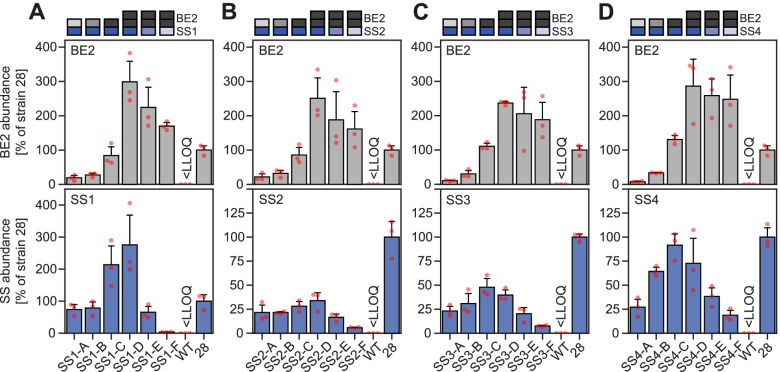
Relative protein abundances of BE2 and SS proteins determined by parallel reaction monitoring (targeted proteomics) at the 3-h time point. Quantification used 2–3 surrogate peptides for each protein and isotopically labelled standard peptides for normalization. Protein abundances (means ± S.D., *n* = 3 replicate cultures; red points show the data from each replicate) are expressed relative to strain 28, which expresses all SS and BE proteins controlled by the *pGAL1* promoter [25]. Data was partly replotted in the individual panels for ease of comparison. In the wild-type (WT) samples, the signals of all SS and BE2 surrogate peptides were below the lower limit of quantitation (LLOQ), as expected from the absence of the proteins. Numerical data is provided in Additional file 6, sheet 7

In all of the strain sets, the abundance of BE2 protein sequentially increased from strains A to D (Fig. 6). This was expected from the use of progressively stronger BE2 expression constructs, although in most cases the measured increases were greater than anticipated from the YFP reporters. In strains D, E, and F, BE2 abundance decreased slightly, even though the expression constructs in all three strains were identical, for reasons that were unclear.

For the SS proteins, the abundances decreased from strain D to F, which was again consistent with the use of progressively weaker expression constructs. However, despite the use of identical expression constructs, strains A and B, which typically produced insoluble glucans, unexpectedly gave lower SS abundances than C and D. These differences probably have a biological rather than technical origin, since similar differences were observed by Western blotting using different extraction methods and they were also apparent in native gels of enzyme activities (Additional file 1: Figs. S9 and S12, Additional file 7). Possible biological causes, explored by re-analysis of shotgun proteomics data, are presented in Additional file 1: Table S4.

**Fig. 7:**
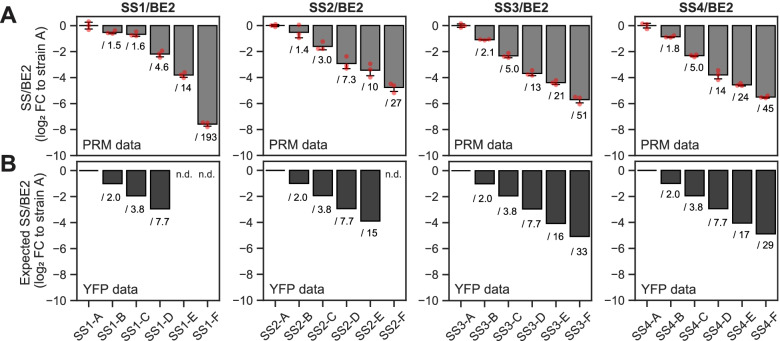
Relative ratios of each SS to BE2 determined by targeted proteomics follow the predictions from YFP measurements. Log_2_ fold changes (FC) of enzyme ratios relative to strain A from each set. Numbers below the bars indicate the non-logarithmic fold changes. Numerical data is provided in Additional file 8. **A** Changes in SS/BE2 ratios as measured by parallel reaction monitoring (PRM). Data are means ± S.D. (*n =* 3 replicate cultures), with red points showing the individual replicates. **B** Changes in molar ratios of each SS to BE2 were estimated from YFP measurements of tagged protein forms. Ratios are means (*n =* 3 replicate cultures, except for strains with BE2-YFP, where *n* = 4) re-calculated from data shown in Additional file 1: Table S1. n.d., not determined

We re-calculated our PRM data to assess the SS/BE2 ratios in our yeast strains, expressing the ratios relative to those observed in strain A from each set. This showed that, despite the fact that some protein levels were not as expected, the relative SS/BE2 ratio sequentially decreased from strains A through to F in all cases (Fig. 7A). The ratios also covered a broad spread in each strain set, as fold changes ranged from 27 (compare strains SS2-A and SS2-F) to 193 (compare strains SS1-A and SS1-F). Thus, the observed modulations in enzymatic SS/BE2 ratios largely follow the expectation from the YFP measurements of the tagged versions (Fig. 7B).

## Discussion

This work shows that it is possible to systematically vary the expression levels of enzyme combinations in yeast—in this case SS and BE pairs. By doing so, we gain a deeper insight into the interdependent functional relationship between them in terms of the glucan structure they produce. This level of insight can be difficult to obtain using isolated proteins in vitro, where it is hard to obtain a steady-state of glucan production. It is also difficult in the complexity of a native plant system, where many enzymes are present, and functional overlap between different enzyme isoforms can obscure phenotypes.

We argue that finely tuned heterologous expression in yeast provides an optimal system to address the complexity of a process like starch synthesis. It allows control over protein complement and, as shown here, protein amounts. Furthermore, after induction, glucans accumulate from essentially zero to high levels in a robust and reproducible fashion.

### Influence of SS/BE enzyme ratios on glucan structure and solubility

**Fig. 8:**
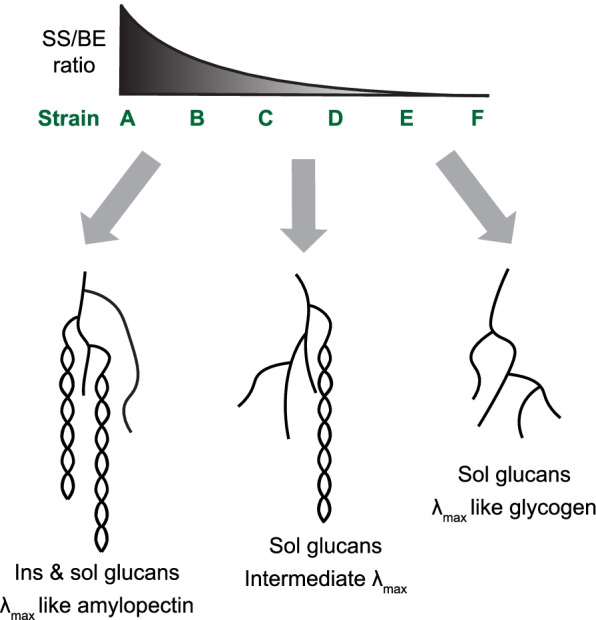
Model of the relationship between the SS to BE ratio, glucan structure, and solubility. In strains A and B, where the SS/BE ratio is highest, a mixture of insoluble (ins) and soluble (sol) glucans is formed. These glucans are characterized by abundant long chains that frequently adopt double-helical secondary structures as in plant amylopectin, despite probably imperfect branching patterns. If the SS/BE ratio is decreased (strains C and D), chains become shorter and secondary structures less likely, resulting in exclusively soluble glucans. In strains E and F, where elongating SS activity is limited, glucans have a large portion of short glucan chains that directly derive from BE activity and have not (or only little) been elongated by a SS. These glucans have a wavelength of maximum absorption after iodine complexion (*λ*_max_) similar to glycogen, indicative of few to no secondary structures

Our use of enzyme pairs—a single SS and BE2—was important to facilitate ratiometric analyses, and discern the features of the different SS isoforms. However, it did not allow us to recapitulate starch biosynthesis, which would require the expression of many more enzymes [25]. In all yeast strain sets, we observed both common trends and some unique SS-dependent features. In general, the higher the SS/BE ratio, the more long glucan chains were present and the higher were the *λ*_max_ values, indicative of more secondary structures (summarized in Fig. 8). This is consistent with the starch phenotypes of plant mutants deficient in a large fraction of their BE activity (such as the maize *amylose extender* or pea *rugosus* mutants), which produce starches with less frequently branched amylopectin accompanied by an increase in apparent amylose [44, 45]. (Apparent amylose increases because long amylopectin chains can have amylose-like characteristics and partly because lower levels of amylopectin lead to increased relative amounts of true amylose synthesized by GBSS [46].) Such starches are valuable for industry because of their special physicochemical properties, such as higher peak gelatinization temperature [47, 48]. They also display reduced digestibility in the human intestine, which is associated with health benefits regarding insulin response and favorable gut microbiota [49, 50]. However, the benefits of these starch traits are often compromised by low yields [51, 52], since reduced BE activity limits the number of ends for SSs to act on. This is in line with the decreases in total glucan amounts in strains A and B in our yeast strain sets (Fig. 4).

Our quantitative analysis demonstrates that even small changes in SS/BE ratio can confer appreciable differences in glucan structures, providing a rational framework to generate the optimal balance between beneficial starch properties and yields in crops. Specifically, we observed that as little as a ~3-fold change in SS/BE ratio between individual strains could have a marked impact on the glucan’s chain lengths, the formation of secondary structures and its solubility. For instance, decreasing the SS/BE ratio by ~2–3 from strain B to C in each set was sufficient to render all glucans soluble (Fig. 4). Further decreasing the SS/BE ratio by ~3 from strain SS1-C to SS1-D increased the number of short chains and reduced *λ*_max_ values even more, indicative of abolishment of any remaining secondary structures. These differences were similar between strains C and D from the set with SS4 but were weaker in those with SS2 and SS3 (Figs. 4 and 5). In some cases, structural differences were apparent with one type of analysis but not another. For example, the *λ*_max_ values of soluble and insoluble glucans from strain SS1-A and SS3-B were similar, yet the insoluble glucans had considerably more long chains (Fig. 5A). This reflects the known difficulties in fully capturing glucan structure with any one analytical approach [53], and the need to combine multiple techniques, as done here.

The transition from only soluble to some insoluble glucans in yeast occurred at an estimated protein ratio of ~2, i.e., when there were approximately twice as many SS molecules as BE molecules in the yeast cells (typically at strain B from each set; Fig. 4). It is important to note that this ratio is estimated from the YFP signals obtained in the reporter strains and that the actual ratios of the untagged proteins are unknown both in our yeast system and in plants. Furthermore, the activities (turnover numbers) of the individual SS likely vary, so that the number of protein molecules is only a proxy for elongation capacity. Nevertheless, the consistency between the strain sets is remarkable, especially given that differences in the chain length profiles generated by the various SS isoforms was clear (Fig. 5). Particularly apparent is the distinct preference of SS1 for producing DP8, compared with the other SS isoforms, which was supported by the close resemblance of the yeast glucan CLDs to those of the starches from the corresponding Arabidopsis lines, i.e., to mutants possessing only or predominantly the SS expressed in the yeast. These observations are important because both chain lengths and branch point distributions are considered important in influencing amylopectin’s capacity to form secondary structures and crystallize [20, 54]. However, our data suggest that merely the elongating to branching ratio can influence glucan solubility, regardless the elongating enzymes’ specificities.

The insoluble glucans made in yeast had *λ*_max_ values very close to those of plant amylopectins (Fig. 5), indicating that similar secondary structures are formed. However, we reemphasize that the insoluble glucans here, being made by just two enzymes, are unlikely to possess the refined crystalline lamellae of amylopectin in starch granules. It is more likely that abundant long chains are responsible for driving the transition between soluble and insoluble states. In order to obtain insoluble glucans in yeast with a branching frequency comparable to plant amylopectin may require not only a mix of SSs to confer an optimal CLD but also the presence of the ISA1-ISA2 debranching activity in addition to BE. Studies from plant mutants lacking this ISA debranching enzyme revealed that its glucans are enriched in branches that are very close to each other and consequently remain largely soluble as phytoglycogen [17, 55, 56]. ISA activity was not included in the strains described here but was previously shown to promote the formation of insoluble glucans in yeast cells expressing a full complement of Arabidopsis starch biosynthetic enzymes [25]. Even in that case, however, not all of the glucans were insoluble, and their structures did not exactly match that of Arabidopsis amylopectin. One proposed reason was that, being all expressed from inducible *GAL* promoters, the biosynthetic enzymes were not present at the physiologically correct stoichiometry. This is a possibility we can now address using the tools devised here. Another exciting possibility is that additional protein factors, beyond the currently known enzymatic set, also contribute to the crystallization process—promoting a change in state of glucans via non-enzymatic means. Potential candidates for such a role are the ESV1 (EARLY STARVATION1) and LESV (LIKE ESV1) proteins. These apparently non-enzymatic proteins are found associated with starch granules from different botanical sources [57]. Based on the premature degradation of leaf starch in the Arabidopsis *esv1* mutant, it was speculated that these proteins may be involved in facilitating amylopectin crystallization [57]. Although no molecular mechanism was proposed for this hypothesized function, we suggest that our yeast system represents an ideal, simplified system in which to test their functions and develop a mechanistic model.

### On the modulation of gene expression in yeast

Our systematic approach to alter gene expression in yeast is exceptional in several aspects. First, we fused the fluorescence reporter to each of our CDS of interest (instead of analyzing only the reporter itself) and analyzed promoters and terminators in various combinations. This showed that each part impacts gene expression largely independent of the other components and that promoter/terminator activities can differ when combined with certain CDS. It also enabled the promoter/terminator activities to be estimated by regression analysis rather than by single comparisons (Fig. 3). Second, we show that careful pre-selection of promoters affords stable expression during the switch from glucose to galactose in the medium used to induce ADPglucose production (Fig. 1C and Additional file 1: Figs. S3 and S4). Thus, the cellular concentration of the selected proteins is maintained despite other changes in transcriptional activities [26]. Third, we accurately quantified the levels of our proteins of interest by targeted proteomics. This demonstrated the extent to which the anticipated modulation in expression holds true when proteins are expressed in their native, untagged form in our growth regime (Figs. 6 and 7).

The present YFP reporter data largely recapitulate previous observations by Keren et al. [26] and Yamanishi et al. [27], although the contribution of the terminators on expression levels was smaller here than previously reported. The inconsistencies in activities probably stem from cumulative effects of differences in constructs (e.g., module length), experimental setup (e.g., growth conditions), and data analysis (e.g., background correction; summarized in Additional file 1: Table S2), many of which have been shown to impact gene expression [58–60]. In addition, we show that the CDS can have a marked influence on the promoter and terminator activities, since their strengths deviated when driving SS1-YFP and, sometimes, SS2-YFP (Fig. 3). Most upstream regulatory sequences for binding transcription factors in yeast function only if placed 5’ of the transcription start site, but exceptions exist [61–63]. Thus, it is possible that, within the CDS used, cryptic regulatory sequences are present. Also mRNA secondary structures formed around the AUG start codon can impact gene expression and depend partly on the CDS [60].

Interestingly, our protein quantification by targeted proteomics revealed lower abundances of SS and BE2 proteins in strains producing insoluble glucans, despite partly employing identical expression units (strains A and B from each set; Fig. 6). There are various possible reasons for these observations, such as changes in promoter activities or translation rates as a metabolic response to glucan accumulation. However, given that the changes occurred for proteins driven by various promoter/terminator combinations, they are unlikely to derive from specific regulation of the genetic parts. The differences are also unlikely to be caused by the use of multi-gene expression constructs, e.g., by upstream terminators influencing the activities of downstream promoters [64], since they also concerned SS3 and SS4, which were expressed by single expression units.

It further seems unlikely that glucan biosynthesis per se has a global impact on the yeasts proteome, but selective degradation via autophagy could occur. Indeed, label-free shotgun proteomics comparing strains 29 (producing insoluble glucans) and 48A (producing no glucans; Additional file 1: Table S4) revealed very few proteins as changed in abundance, but Atg8, a key component of autophagy [65, 66], was ~3× more abundant in the yeast strain 29. Thus, autophagosomes could potentially clear glucans from the cytosol, along with associated biosynthetic enzymes. However, gene ontology analysis did not identify autophagy-related proteins as enriched, and the extent to which autophagy occurs and reduces SS and/or BE2 abundance is presently unknown. Regardless of the cause, the occurrence of unanticipated changes in protein abundance among our strains illustrates the importance of having procedures in place to accurately quantify protein abundances. Quantifications could also be conducted by immunoblotting, where antibodies are available, but a major benefit of proteomics is that multiple signals are assessed, i.e., multiple fragment ions of each peptide and multiple peptides for each protein, as opposed to a single protein band in immunoblotting, adding robustness [67]. In our PRM assays, measurement accuracy and reliability are further improved by the use of labelled standards, which can confirm correct peptide identification, allow determining the dynamic linear detection ranges, and could even be developed further to yield absolute quantifications [68].

## Conclusions

In this study, we successfully reconstituted the biosynthesis of plant glucans in yeast and demonstrated the importance of controlling expression levels as well as enzyme type on the glucans produced. Our approach, concentrating on enzymatic pairs consisting of a single SS and BE2, represents a firm starting point for further studies—the expression of more complex protein mixtures, for example, precisely matching the enzyme ratios in yeast to those in plants so as to accurately rebuild plant starch in this heterologous system. It serves as a clean reference system to dissect the specific features of enzymes at the heart of biosynthesis, explaining their individual specificities, regulation, and the functional significance of assembly into protein complexes. It further serves an ideal starting point to explore the roles of factors such as ESV1 and LESV, as well as other proteins whose molecular functions in starch biosynthesis are still poorly understood.

## Methods

### Experimental design and data analysis

Experiments and analyses were conducted non-blinded. Unless otherwise noted, yeasts and samples were grouped according to replicate number (not according to line/genotype) in a non-random way during growth and processing. Criteria for data exclusion, if any, are described in the individual sections. Regression analyses were conducted using the least ordinary square model from the Statsmodels Python module.

### Chemicals, media, and plant materials

Chemicals were purchased from Sigma-Aldrich, unless otherwise specified. Rich media (yeast extract peptone dextrose [YPD] medium, yeast extract peptone [YP]-galactose medium), synthetic complete (SC) media, and SC plates containing 5-fluoroorotic acid were prepared as described [25]. For selection of yeast transformants by antibiotic resistance, freshly prepared YPD medium containing 2% (w/v) bacto agar (BD) was complemented with sterile-filtrated geneticin (G418; Calbiochem) or hygromycin (Roth) to reach final concentrations of 150 and 200 mg L^−1^, respectively. YP-maltose plates were prepared by adding sterile-filtrated maltose stock (Fluka) to autoclaved YP medium containing 2% (w/v) bacto agar (BD) to a final concentration of 2% (w/v) maltose.

Alleles of *Arabidopsis thaliana* mutants are provided in Additional file 9, sheet 2 [13, 16, 19, 25, 39, 41, 69]. Mutants were either of ecotype Wassilewskija (WS; for CLDs and Western blots) or Columbia-0 (Col-0; for Western blots). Plants were grown on soil in a CLF Plant Climatics Percival under a 12-h photoperiod as described [70]. For CLDs, 4-week-old plants were harvested at the end of the light period. For Western blots, leaf material was collected at the middle of the day from plants grown for 4 to 7 weeks.

Potato amylopectin is tuber starch from the *amf* amylose-free potato variety [71]. Amylopectin from *Arabidopsis thaliana* is starch purified from *gbss* mutants [11].

### Cloning of plasmids

Vectors, primers used to create them, and sequences of all generated plasmids are available in Additional file 10 [25, 32, 72, 73]. To express the *E. coli* glgC-TM fused to a C-terminal HA-tag and carrying three amino acid changes to render it insensitive to allosteric regulation (R67K, P295D, G336D) [74], we used the same construct (pBP38_pGAL1-GlgC-TM-HA in pGSY1) as in our previous study [25].

#### Plasmids to delete glycogen-metabolic genes

Plasmids to knock-out genes without expressing a transgene at these loci based on the integrative USER-cloning vector pX-2 [72] and were modified to target the specific loci as previously described [25]. Most of these vectors (Additional file 10, sheet 4) were further adapted by removing the majority of *tADH3* and *tCYC1*—present in pX-2 for expression of inserts—by cutting the vectors with S*ac*II and *Mlu*I and inserting a short fragment formed by two primers (5′-GGTGTGCTGTACAGA-3′; 5′-CGCGTCTGTACAGCACACCGC-3′). Plasmids were verified by sequencing of the modified parts.

#### Golden-gate cloning for heterologous gene expression

Golden-gate cloning was performed as described [32], employing four out of five positions for multi-gene assembly. Part plasmids were verified by sequencing of the inserts. The correctness of pre-assembled integration vectors, cassette vectors, and multi-gene plasmids was confirmed by partial sequencing of the inserts and diagnostic restriction digests.

To clone new parts containing yeast genomic fragments into pYTK001 [32], the corresponding sequences were PCR-amplified from wild-type CEN.PK113-11C *S. cerevisiae*, except for *pGAL1* and *tCYC1* where the same sequences were used as in our previous study [25] for ease of comparison. In case of *pCWP2* and *pCUP1*, the last nucleotide before the ATG start codon had to be changed from A to T to create the overhangs compatible with modular cloning. The lengths of promoters and terminators were generally equal to the sequences tested before [26, 27], but *pCWP2* was shortened to ca. 600 bp and *pYJL068C* elongated to ca. 540 bp to have more uniform lengths among the promoters.

Arabidopsis CDS of SS1 to SS3 were domesticated in pYTK001 [32] less their putative chloroplast transit peptides predicted by ChloroP [75], adding a C-terminal glycine and serine to the CDS to follow the conventions of the yeast modular cloning toolkit [32]. BE2 and BE3 were domesticated similarly but removing shorter transit peptides, since peptides within the predicted transit peptides had been detected in proteomics from Arabidopsis leaves [76] (Additional file 10, sheet 1). These shortened putative transit peptides matched the second most probable cleavage site according to ChloroP. For SS2, we used the same SS2 sequence codon-optimized for *S. cerevisiae* as before [25]. The CDS of SS4 less its chloroplast transit peptide as predicted by ChloroP was domesticated in pJET2.1 (Life technologies; for part 3) and *Sma*I-linearized pENTR11 (Invitrogen; for part 3a) because of difficulties to clone it into pYTK001.

Four spacer plasmids in pYTK001 were generated by adding forward and reverse primers that formed overhangs compatible to the *Bsm*BI-reaction with pYTK001 (Additional file 10, sheet 1). These spacer plasmids served as a replacement for a transcriptional unit at position 1, 2, 3, or 4 when required. To replace two transcriptional units, forward and reverse primers that form overhangs mimicking the connectors were directly added to the *Bsm*BI golden-gate reaction (Additional file 10, sheet 1).

### Generation of yeast strains

The genotypes of *Saccharomyces cerevisiae* (yeast) strains used in this study are presented in Additional file 9, sheet 1. All strains derive from CEN.PK113-11C (*MATa MAL2-8*^*C*^*SUC2 his3D ura3-52*), kindly provided by Prof. Barbara A. Halkier (University of Copenhagen, Denmark) and named wild type here. Yeast strains were generated by integrating constructs at gene-encoding loci, thereby deleting them, or at the intergenic loci *XII-2* and *XII-5* on chromosome 12 [72]. Yeast strains 28 and 29 are described in [25].

Yeast transformation was essentially conducted as described [72]. Correct construct integration in streak-purified transformants was tested by PCR combining a primer complementary to the new insert and a primer complementary to the genomic region up- or downstream of the integration site. Recycling of the *URA3* marker, test for petiteness, and growth tests on SC-ura plates were performed as previously [25], and the successful loss of the *URA3* marker was confirmed by PCR.

#### CRISPR/Cas9 mediated gene editing of maltase genes

Gene edits of maltase genes were conducted using the method for iterative markerless editing of the yeast genome [73] (Additional file 10, sheet 5). This involved three components that were simultaneously transformed into yeast: (1) a linearized guide RNA construct; (2) a CRISPR/Cas9 expression plasmid (pWS158) [73]; and (3) double-stranded DNA for gene editing during the repair of the Cas9 cut. For the first component, potential protospacers were identified by Cas-Designer [77] and filtered for complementarity to all available maltase gene sequences from CEN.PK113-7D, close proximity (< 140 bp) to the maltase ATG start codons and the prediction of no off-targets with 0 or 1 mismatches in Cas-OFFinder [78]. Three guide RNA targets (A-MALx2; B-MALx2; C-MALx2) were separately cloned into pWS082 as described [73], and 200 ng of *Eco*RV-digested plasmids was used for each transformation. For the second component, pWS158 [73] was digested with *Bsm*BI (NEB) to release the drop-out marker, and 100 ng gel-purified vector backbone were used per transformation. For the third component, linear repair dsDNA constructs were designed to introduce an in-frame stop codon close to the cut site, followed by a unique barcode. These constructs were flanked by ~60-bp-long arms homologous to *MALx2* genes and generated by PCR amplification of two partly overlapping forward and reverse primers (Additional file 10, sheet 5) using DreamTaq DNA polymerase (Thermo Fisher Scientific). Two micrograms gel-purified PCR products were used in each transformation.

The three components were simultaneously transformed into strain 362.1 using a protocol slightly modified from Gietz and Schiestl [79]. Uracil prototroph transformants were streak-purified and amplified several times on YPD plates to help curation of the CRISPR/Cas9 expression plasmid. Loss of this plasmid was confirmed by the inability of yeasts to grow on SC-ura plates. Purified strains were tested for impaired maltose metabolism by their inability to grow on YP-maltose plates and absence of maltase activity. Correct gene editing was confirmed by PCR (for primers, see Additional file 10, sheet 5) and Sanger sequencing of the amplicons.

### Growth of yeast strains in liquid culture

Replicate cultures derive from independent pre-cultures of the same yeast strain. Unless otherwise noted, yeasts were grown in deep-well plates (DWPs). Therefore, a small amount of yeast cells from a YPD plate was resuspended in YPD in a 96-DWP (Brand), the plate covered with sterile aluminum foil and shaken overnight at 300 rpm and 30 °C at an angle of ca. 20°. Pre-cultures were used to inoculate main cultures in YP-galactose to a starting OD of ca. 0.3 in 2-ml cultures in a 24-DWP (Axygen, Corning Life Sciences) or 400-μl cultures in a 96-DWP. For non-inducing controls, main cultures were prepared in YPD with a starting OD of ca. 0.2. Plates were closed with a sterile gas-permeable BREATHseal foil (Greiner Bio-One) and shaken at 30 °C and ~300 rpm at an angle of ca. 20° until further processing as described in the individual sections.

### Fluorescence measurements

Fluorescence parameters and OD were acquired after overnight growth of the pre-culture in YPD (time point 0h), and after 3 and 5 h growth in YP-galactose in DWPs. To minimize background fluorescence from YP media, cells pelleted from a culture aliquot were washed and reconstituted in water, then assayed for fluorescence and OD. Fluorescence parameters were acquired in 96-well microplates (FLUOTRAC; Greiner Bio-One) in an Infinite M1000 plate reader (Tecan) using excitation at 586 nm / emission at 612 nm for mCherry and excitation at 507 nm / emission at 533 nm for YFP (10 nm bandwidth in all cases). YFP signals were normalized to cell density or mCherry fluorescence. mCherry and YFP (but not OD) fluorescence were acquired from the same well, allowing correcting for variation in sample volume, thereby decreasing technical error. From all normalized YFP signals, we furthermore subtracted the normalized YFP fluorescence from strain 361, which contains the mCherry but no YFP reporter. A single replicate of strain 489 was excluded from data analysis since it became contaminated with another culture during processing.

### Quantitative and structural analysis of glucans

Yeasts grown in DWPs were harvested after 5.75 h growth in YP-galactose by centrifugation at 3000*g* for 3–5 min, washed once with water, and resuspended in water. An aliquot of the suspension was used to determine mCherry fluorescence and OD as described above. The remaining cells were pelleted, flash frozen in liquid nitrogen and stored at −80°C. Wet weights (WW) of the cell pellets were calculated from the average of WW according to mCherry and OD measurements, assuming that 1 mg cells (WW) in a plate reader well gave an mCherry signal of 1.51×10^5^ and an OD of 0.51 (Additional file 1: Fig. S10). Soluble and insoluble glucans from yeasts were prepared using homogenization in perchloric acid and methanol precipitation as described [25]. Briefly, cell suspensions were homogenized in 1.12 M perchloric acid, centrifuged at 6000*g* for 5 min, and separated into the insoluble fraction (pellet) and soluble fraction (supernatant). After neutralization of the soluble fraction, polysaccharides present in this fraction were precipitated in 80 % (v/v) methanol. Soluble glucans constitute these methanol-precipitable glucans. Insoluble glucans constitute all glucans present in the insoluble fraction. Soluble and insoluble glucans were quantified in an enzymatic assay as described [80]. All measurements were included in the present data set.

Arabidopsis starches were extracted in perchloric acid as described previously [20]. Preparation of glucans for CLD and data acquisition were essentially conducted as described [25]. Occasionally, samples were excluded from the CLD data analysis if peak areas were insufficient peak areas or they showed signs of degradation. Iodine absorption spectra to determine *λ*_max_ values, iodine staining of cells grown on SC plates, light microscopy of cells grown in liquid culture were essentially conducted as previously described [25].

### Native PAGE (polyacrylamide gel electrophoresis) and maltase activity assays

Yeasts were cultivated in shake flasks using YPD and YP-galactose as media in pre- and main cultures, respectively, and harvested after 3 h (for native PAGE) or 5 h (for maltase activity assays) shaking in YP-galactose and subjected to soluble protein extraction as described [25]. Native PAGE, including preparation of Arabidopsis extracts, was conducted as described [25].

Maltase activities were assayed in reactions containing 0.75 μg ml^−1^ native yeast protein, 100 mM potassium phosphate buffer pH 6.5, 1 mM dithiothreitol (DTT), and a single carbohydrate substrate (160 or 40 mM maltose, 40 mM maltotriose, maltotetraose, maltopentaose, or maltohexaose, or 17.5 mg ml^−1^ glycogen type II from oyster which had been washed by precipitation in 75% [v/v] methanol, reconstituted in water and solubilized by boiling at 99°C for 10 min prior to use). Reactions were split and incubated at 28°C for 15 and 30 min, respectively, and enzymatic activities were stopped by boiling the assays at 99°C for 10 min in a water bath. Glucose produced by maltase activity was quantified in enzymatic assays as described [80]. Starting glucose concentrations (time point 0 min) constitute the sum of glucose present in assays in which once the protein extract and once the substrate was replaced by water, to account for free glucose present in the protein extracts and substrate preparations.

### Protein extraction for Western blotting

Yeasts were cultivated in DWPs, harvested after 3 h cultivation in YP-galactose by centrifugation at 3000*g* for 3–5 min, and the cell pellets washed twice with water and further treated as described in the individual sections. For plant samples, extraction of total leaf protein was conducted as described [70], with replicates deriving from individual plants.

For comparison of YFP-tagged proteins present in the soluble and total protein extracts (Additional file 1: Fig. S5), the washed cell pellets were resuspended in 100 μl native extraction buffer [100 mM 3-(N-morpholino)propanesulfonic acid (MOPS), pH 7.75 at 4 °C, 10% (v/v) glycerol, 1 mM ethylenediaminetetraacetic acid (EDTA), complemented with protease inhibitor (Complete EDTA-free, from Roche)], frozen in liquid nitrogen, and stored at −80°C. After thawing on ice, 100-μl glass beads (425–600 μm diameter, acid washed) were added and the cell suspensions homogenized by vortexing at full speed for 14 min at 4 °C with cooling in between. The homogenates without glass beads were transferred to a fresh tube and split into two parts, one for soluble protein extraction and one for total protein extraction. For total protein extracts, the homogenates were supplemented with sodium dodecyl sulfate (SDS) to reach 2% (w/v) final concentration, boiled for 5 min at 98°C, cleared by centrifugation and stored at −20 °C until use. For soluble protein extracts, the homogenate was cleared by centrifugation, frozen in liquid nitrogen, and stored at −20°C. In both extracts, protein concentrations were determined by bicinchoninic acid assay (BCA) using bovine serum albumin (BSA) as standard. Protein extracts were supplemented with SDS-PAGE loading buffer and boiled at 95°C for 5 min prior to loading.

For comparison of extraction by urea and SDS (Additional file 1: Fig. S12), the washed yeast suspensions were split into two halves. The first half was processed with urea exactly as for protein extraction for targeted proteomics and stored at −80°C until use. The other half of the suspension was used for total protein extraction using SDS. For the latter, extracts were prepared as described for total proteins above, but using 100 mM Tris pH 8.2 at 4 °C, complemented with protease inhibitor (Complete EDTA-free, from Roche) as extraction buffer and supplementing the homogenate with SDS and DTT to final concentrations of 2% (w/v) and 30 mM, respectively, before boiling and clearing. Protein concentrations from the urea and SDS extracts were determined by BCA assay and Qubit protein assay (Invitrogen), respectively, using BSA as standard.

### Western blotting and antibodies

Western blotting and signal detection of IRDye secondary antibodies were conducted as described [70]. Primary and secondary antibodies including dilutions, sources, and validation are detailed in Additional file 9, sheet 3. Quantification of total protein on membranes was performed using Revert 700 total protein stain kit (Li-Cor) and Image Studio Lite Ver. 5.2 software (Li-Cor).

### Proteomics

PRM assays used 2–3 surrogate peptides for each protein of interest and normalization to heavy-labelled standard peptides. Methods are detailed in Additional file 11 [81–86], information on proteins and surrogate peptides are given in Additional file 6 [74, 81, 87], and calibration curves of the standard peptides are provided in Additional file 12. Label-free shotgun proteomics used to compare strains with and without glucan production (Additional file 1: Table S4) is detailed in Additional file 13 [25, 88–91] and in Additional file 14.

### Accession numbers and *S. cerevisiae* loci

The Arabidopsis Genome Initiative gene codes for *Arabidopsis thaliana* genes analyzed in the present study are as follows: At5g24300 (*SS1*), At3g01180 (*SS2*), At1g11720 (*SS3*), At4g18240 (*SS4*), At5g03650 (*BE2*), At2g36390 (*BE3*). glgC from *Escherichia coli* has the GenBank accession number V00281.1. The gene IDs of the S. cerevisiae loci are the following: YFR015C (*GSY1*), YLR258W (*GSY2*), YPR184W (*GDB1*), YEL011W (*GLC3*), YPR160W (*GPH1*), YKR058W (*GLG1*), YJL137C (*GLG2*), YGR292W (*MAL12*), YSC0005 (*MAL22*), YBR299W (*MAL32*), YSC0010 (*MAL42*).

## Supplementary Information

**Additional file 1: Table S1**. Yeast strains generated for glucan analysis. **Table S2**. Regression analysis of the influence of the promoter or terminator on expression levels. **Table S3**. Summary of yeast maltase activity towards different glucan substrates. **Table S4**. Proteins changes between yeast strain 29 and strain 48A measured by shotgun proteomics. **Figure S1**. Control glucan measurements. **Figure S2**. Quality control of YFP fluorescence measurements. **Figure S3**. Stability of normalized YFP fluorescence over the time course. **Figure S4**. Stability of expression conferred by different promoters. **Figure S5**. Immunoblots of YFP-tagged proteins expressed in yeast. **Figure S6**. Maltase activity towards different glucan substrates. **Figure S7**. Gene editing of all *MALx2* genes results in loss of maltase activity. **Figure S8**. Western blots of untagged SS and BE proteins. **Figure S9**. Native PAGE monitoring the activities of starch synthases (SSs) and branching enzymes (BEs) in yeast. **Figure S10**. Relationships between yeast mCherry fluorescence, optical density (OD) and wet weight (WW). **Figure S11**. Iodine staining of cell patches grown on galactose- or glucose-containing plates. **Figure S12**. Quantification of SS1 abundance after different extraction methods by Western blotting. **Figure S13**. Assessment of linear range for quantification of SS1 by Western blotting.

**Additional file 2.** Numerical data and statistics of regressions of YFP reporter data associated with Figs. 1, 2, and 3.

**Additional file 3.** Numerical data of maltase activity assays associated with Figures S6-S7.

**Additional file 4.** Numerical data of glucan measurements associated with Fig. 4.

**Additional file 5.** Numerical data of chain-length distributions and lambda max values associated with Fig. 5.

**Additional file 6.** Targeted proteomics - Numerical data of Fig. 6 and information on proteins, surrogate peptides, transitions and runs.

**Additional file 7.** Numerical data of Western blot quantification associated with Figures S12-S13.

**Additional file 8.** Numerical data of SS/BE2 ratios associated with Fig. 7.

**Additional file 9.** Yeast strains, plant lines and antibodies used in the present study.

**Additional file 10.** Vectors and CRISPR/Cas9 constructs used in the present study.

**Additional file 11.** Detailed methods of targeted proteomics.

**Additional file 12.** Targeted proteomics - Calibration curves of heavy-labelled standard peptides tested for quantification.

**Additional file 13.** Shotgun proteomics: Detailed methods.

**Additional file 14.** Shotgun proteomics: List of quantified proteins and numerical data.

## Data Availability

The datasets supporting the conclusions of this article are included within the article and its additional files. The raw mass spectrometry data of targeted proteomics and the associated Skyline analysis files are available via ProteomeXchange (dataset identifier PXD033364) and Panorama Public [92]. The proteomics data of the label-free shotgun experiment have been deposited to the ProteomeXchange Consortium via the PRIDE [89] partner repository with the dataset identifier PXD032241 [93]. All yeast strains and other materials generated in this study are available upon request.

## Abbreviations

ADPglucose: Adenosine 5’-diphosphate-glucose
AGPase: ADPglucose pyrophosphorylase
BE: Branching enzyme
BCA: Bicinchoninic acid assay
BSA: Bovine serum albumin
CDS: Coding sequence
CLD: Chain length distribution
Col-0: Columbia-0
CRISPR: Clustered regularly interspaced short palindromic repeats
DP: Degree of polymerization
DTT: Dithiothreitol
DWP: Deep-well plate
EDTA: Ethylenediaminetetraacetic acid
GBSS: Granule-bound starch synthase
HPAEC-PAD: High-performance anion-exchange chromatography with pulsed amperometric detection
ISA: Isoamylase
*λ*_max_: Wavelength of maximum light absorption after complexion with iodine (I_2_/KI solution or vapor derived from it)
MOPS: 3-(N-morpholino)propanesulfonic acid
OD or OD_600_: Optical density at 600 nm
PRM: Parallel reaction monitoring
R^2^: Coefficient of determination
SC-medium: Synthetic complete medium
S.D.: Standard deviation
SDS: Sodium dodecyl sulfate
PAGE: Polyacrylamide gel electrophoresis
S.E.: Standard error
SS: Starch synthase
WT: Wild type
WW: Wet weight
WS: Wassilewskija
YFP: Yellow fluorescent protein
YP(D): Yeast extract peptone (dextrose)
