# Supplementary material for: Tuning heterologous glucan biosynthesis in yeast to understand and exploit plant starch diversity
Source: BMC Biol. 2022 Sep 24;20:207. doi: 10.1186/s12915-022-01408-x (PMC9509603; doi:10.1186/s12915-022-01408-x)
Supplement: Supplementary file 1 — Additional file 1: Table S1. Yeast strains generated for glucan analysis. Table S2. Regression analysis of the influence of the promoter or terminator on expression levels. Table S3. Summary of yeast maltase activity towards different glucan substrates. Table S4. Proteins changes between yeast strain 29 and strain 48A measured by shotgun proteomics. Figure S1. Control glucan measurements. Figure S2. Quality control of YFP fluorescence measurements. Figure S3. Stability of normalized YFP fluorescence over the time course. Figure S4. Stability of expression conferred by different promoters. Figure S5. Immunoblots of YFP-tagged proteins expressed in yeast. Figure S6. Maltase activity towards different glucan substrates. Figure S7. Gene editing of all MALx2 genes results in loss of maltase activity. Figure S8. Western blots of untagged SS and BE proteins. Figure S9. Native PAGE monitoring the activities of starch synthases (SSs) and branching enzymes (BEs) in yeast. Figure S10. Relationships between yeast mCherry fluorescence, optical density (OD) and wet weight (WW). Figure S11. Iodine staining of cell patches grown on galactose- or glucose-containing plates. Figure S12. Quantification of SS1 abundance after different extraction methods by Western blotting. Figure S13. Assessment of linear range for quantification of SS1 by Western blotting. [file 12915_2022_1408_MOESM1_ESM.pdf]

Additional File 1

Supporting Information for:

Tuning heterologous glucan biosynthesis in yeast to understand and exploit plant starch diversity

Barbara Pfister[1*], Jessica M. Shields[1,2], Tobias Kockmann[3], Jonas Grossmann[3,4], Melanie R. Abt[1], Martha Stadler[1], and Samuel C. Zeeman[1]

[1] Institute of Molecular Plant Biology, ETH Zurich, 8092 Zurich, Switzerland

[2] Current address: College of Medicine and Health, University of Exeter, Exeter, UK

[3] Functional Genomics Center Zurich, ETH Zurich, 8057 Zurich, Switzerland

[4] SIB Swiss Institute of Bioinformatics, 1015 Lausanne, Switzerland

* Corresponding author; e-mail: bpfister@ethz.ch

	Variable *SS* construct					Constant *BE2* construct					Expected ratio SS/BE2	
**Yeast strain**	**Expected level**	**Pro-moter**	**CDS**	**Termin-ator**	**Locus**	**Expected level**	**Pro-moter**	**CDS**	**Termin-ator**	**Locus**	**Mean**	**S.D.**
**SS1-A**	strong	*pUBI4*	*SS1*	*tRPL3*	*GDB1*	weak	*pUBI4*	*BE2*	*tENO1*	*GDB1*	8.4	0.8
**SS1-B**	strong	*pUBI4*	*SS1*	*tRPL3*	*GDB1*	medium	*pHHF1*	*BE2*	*tRPL3*	*GDB1*	4.2	0.2
**SS1-C**	strong	*pUBI4*	*SS1*	*tRPL3*	*GDB1*	strong	*pCWP2*	*BE2*	*tRPL3*	*GDB1*	2.2	0.1
**SS1-D**	strong	*pUBI4*	*SS1*	*tRPL3*	*GDB1*	2x strong	*pCWP2*	*BE2*	*tRPL3*	*GDB1, XII-5*	1.09	0.05
**SS1-E**	medium	*pUBI4*	*SS1*	*tENO1*	*GDB1*	2x strong	*pCWP2*	*BE2*	*tRPL3*	*GDB1, XII-5*	n.d.	n.d.
**SS1-F**	weak	*pCUP1*	*SS1*	*tRPL3*	*GDB1*	2x strong	*pCWP2*	*BE2*	*tRPL3*	*GDB1, XII-5*	n.d.	n.d.
**SS2-A**	strong	*pCWP2*	*SS2*	*tUPT7*	*GDB1*	weak	*pUBI4*	*BE2*	*tENO1*	*GDB1*	5.3	0.5
**SS2-B**	strong	*pCWP2*	*SS2*	*tUPT7*	*GDB1*	medium	*pHHF1*	*BE2*	*tRPL3*	*GDB1*	2.7	0.1
**SS2-C**	strong	*pCWP2*	*SS2*	*tUPT7*	*GDB1*	strong	*pCWP2*	*BE2*	*tRPL3*	*GDB1*	1.37	0.04
**SS2-D**	strong	*pCWP2*	*SS2*	*tUPT7*	*GDB1*	2x strong	*pCWP2*	*BE2*	*tRPL3*	*GDB1, XII-5*	0.69	0.02
**SS2-E**	medium	*pUBI4*	*SS2*	*tUPT7*	*GDB1*	2x strong	*pCWP2*	*BE2*	*tRPL3*	*GDB1, XII-5*	0.35	0.02
**SS2-F**	weak	*pUBI4*	*SS2*	*tYOX1*	*GDB1*	2x strong	*pCWP2*	*BE2*	*tRPL3*	*GDB1, XII-5*	n.d.	n.d.
**SS3-A**	strong	*pCWP2*	*SS3*	*tENO1*	*GPH1*	weak	*pUBI4*	*BE2*	*tENO1*	*GDB1*	6.8	0.6
**SS3-B**	strong	*pCWP2*	*SS3*	*tENO1*	*GPH1*	medium	*pHHF1*	*BE2*	*tRPL3*	*GDB1*	3.4	0.1
**SS3-C**	strong	*pCWP2*	*SS3*	*tENO1*	*GPH1*	strong	*pCWP2*	*BE2*	*tRPL3*	*GDB1*	1.76	0.06
**SS3-D**	strong	*pCWP2*	*SS3*	*tENO1*	*GPH1*	2x strong	*pCWP2*	*BE2*	*tRPL3*	*GDB1, XII-5*	0.88	0.03
**SS3-E**	medium	*pHHF1*	*SS3*	*tYOX1*	*GPH1*	2x strong	*pCWP2*	*BE2*	*tRPL3*	*GDB1, XII-5*	0.42	0.05
**SS3-F**	weak	*pUBI4*	*SS3*	*tENO1*	*GPH1*	2x strong	*pCWP2*	*BE2*	*tRPL3*	*GDB1, XII-5*	0.20	0.04
**SS4-A**	strong	*pCWP2*	*SS4*	*tRPL3*	*GPH1*	weak	*pUBI4*	*BE2*	*tENO1*	*GDB1*	4.5	0.5
**SS4-B**	strong	*pCWP2*	*SS4*	*tRPL3*	*GPH1*	medium	*pHHF1*	*BE2*	*tRPL3*	*GDB1*	2.3	0.1
**SS4-C**	strong	*pCWP2*	*SS4*	*tRPL3*	*GPH1*	strong	*pCWP2*	*BE2*	*tRPL3*	*GDB1*	1.17	0.06
**SS4-D**	strong	*pCWP2*	*SS4*	*tRPL3*	*GPH1*	2x strong	*pCWP2*	*BE2*	*tRPL3*	*GDB1, XII-5*	0.59	0.03
**SS4-E**	medium	*pHHF1*	*SS4*	*tUPT7*	*GPH1*	2x strong	*pCWP2*	*BE2*	*tRPL3*	*GDB1, XII-5*	0.27	0.01
**SS4-F**	weak	*pUBI4*	*SS4*	*tUPT7*	*GPH1*	2x strong	*pCWP2*	*BE2*	*tRPL3*	*GDB1, XII-5*	0.15	0.01

Table S1. Yeast strains generated for glucan analysis.

The constructs for expression of untagged starch synthases (SS) and BE2, showing their expression strength expected from our YFP-fusion reporter data, the employed promoter and terminators and targeting loci. The *SS1* or *SS2* expression units were fused to the *BE2* expression unit and targeted to the *GDB1* locus. *SS3* and *SS4* were targeted to the *GPH1* locus. Second copies of *BE2* were inserted into yeast locus *XII-5* (Mikkelsen et al., 2012). All strains additionally contain the mCherry reporter and *pGAL1*::*glgC-TM* for the galactose-inducible production of ADPglucose and, after insertion of the *SS* and *BE2* expression constructs, carry deletions in all glycogen-metabolic genes.

Expected molar ratios of the SS to BE2 were calculated as the quotient of the fluorescence intensity of the strain expressing the corresponding SS-YFP fusion divided by the fluorescence intensity of the strain expressing the BE2-YFP construct (using data YFP fluorescence normalized to mCherry at the 3h time point; **Additional file 8**). As an approximation, we assumed the same expression of BE2 at the *XII-5* locus as at the *GDB1* locus (were the YFP reporter construct had been targeted to). Ratios are means ± SD (n = 3 replicate cultures, except for strains with BE2-YFP, where n = 4). N.d., not determined.

			Excluded data			Statistics of regression						
**Comparison (y *vs.* x)**	**Expected activity**	**Observed activity (slope)**	Strain 1 (x-axis)	Strain 2 (y-axis)	**Comment**	SE (slope)	*p*-Value (slope)	Inter-cept	SE (inter-cept)	*p*-Value (inter-cept)	R^2	*n*
pHHF1 vs. pCWP2	0.37[a]	0.54	n.a.	n.a.	Best fit (R1, Fig. 4A)	0.10	**3.5E-03**	3.38	13.1	8.1E-01	0.84	7
		0.57			Forced through origin	0.03	**1.1E-06**	0.00	n.a.	n.a.	0.99	7
pHSP104 vs. pCWP2	0.12[a]	0.20	#485 (105.1)	#441 (277.5)	Best fit (R1, Fig. 4B)	0.04	**2.6E-03**	3.00	3.27	4.0E-01	0.86	7
		0.23			Forced through origin	0.02	**1.5E-05**	0.00	n.a.	n.a.	0.96	7
		0.57	n.a.	n.a.	Best fit (R2, Fig. 4B)	0.76	4.8E-01	4.25	70.8	9.5E-01	0.09	8
		0.61			Forced through origin	0.33	1.1E-01	0.00	n.a.	n.a.	0.32	8
pUBI4 vs. pCWP2	0.29[a]	0.18	#437 (363.2)	#439 (166.3)	Best fit (R1, Fig. 4C)	0.03	**1.8E-04**	7.49	3.12	4.3E-02	0.84	10
		0.24			Forced through origin	0.01	**8.2E-09**	0.00	n.a.	n.a.	0.98	10
		0.47	n.a.	n.a.	Best fit (R2, Fig. 4C)	0.05	**7.6E-06**	-21.5	7.90	2.3E-02	0.90	11
		0.35			Forced through origin	0.04	**1.7E-06**	0.00	n.a.	n.a.	0.91	11
tUPT7 vs. tRPL3	0.63[b]	0.98	n.a.	n.a.	Best fit (R1, Fig. 4D)	0.09	**8.3E-03**	-14.7	10.5	3.0E-01	0.98	4
		0.87			Forced through origin	0.04	**2.8E-04**	0.00	n.a.	n.a.	0.99	4
tENO1 vs. tRPL3	0.30[b]	0.73	n.a.	n.a.	Best fit (R1, Fig. 4E)	0.06	**2.8E-04**	0.19	6.10	9.8E-01	0.97	6
		0.73			Forced through origin	0.03	**1.3E-06**	0.00	n.a.	n.a.	0.99	6
tMET13 vs. tRPL3	0.04[b] 0.16[c]	0.61	n.a.	n.a.	Best fit (R1, Fig. 4F)	0.13	4.1E-02	-8.43	14.9	6.3E-01	0.92	4
		0.55			Forced through origin	0.05	**1.4E-03**	0.00	n.a.	n.a.	0.98	4
tYOX1 vs. tRPL3	0.04[b] 0.08[c]	0.50	#437 (363.2)	#438 (70.3)	Best fit (R1, Fig. 4G)	0.08	**2.8E-03**	2.61	7.58	7.5E-01	0.91	6
		0.52			Forced through origin	0.03	**2.3E-05**	0.00	n.a.	n.a.	0.98	6
		0.16	n.a.	n.a.	Best fit (R2, Fig. 4G)	0.08	8.4E-02	28.3	12.6	7.5E-02	0.48	7
		0.29			Forced through origin	0.06	**3.6E-03**	0.00	n.a.	n.a.	0.78	7

Table S2. Regression analysis of the influence of the promoter or terminator on expression levels.

All regressions are linear fits (least-ordinary squares model) using YFP/mCherry data from the 3h time point. Best fits not forced through the origin correspond to the regressions presented in **Fig. 3**. “Excluded data” refer to the yeast strains that were omitted in the comparison, with their YFP/mCherry values (a.u.) at time point 3h shown in brackets. *p*-Values of slopes and of intercepts <0.01 are in bold. SE, standard error; n.a., not applicable; R^2, coefficient of determination; *n*, number of means used in the regression.

[a] Expected activities relative to *pCWP2*, re-calculated from the promoter activities reported by Keren et al. (2013). That study used stably integrated YFP reporter constructs, assessed cells at maximum growth rate in synthetic complete medium (with 2% galactose), used a plate reader for fluorescence acquisition, subtracted a yeast background containing no YFP reporters and calculated defined promoter activities as YFP production rate per OD unit per second.

[b] Expected activities relative to *tRPL3*, re-calculated from the terminator activities reported by Yamanishi et al. (2013). That study expressed the GFP reporter from a CEN/ARS plasmid and assessed terminator activities in cells grown for 48 h in synthetic medium (with 2% glucose; without uracil) directly after transformation, using flow cytometry for fluorescence acquisition and calculating fluorescence relative to a reference terminator without background subtraction.

[c] Expected activities relative to *tRPL3*, re-calculated from the terminator activities reported by Yamanishi et al. (2013) as described for foot note (b) but expressing the GFP reporter constructs from a genomic locus and assessing yeasts during exponential growth phase in synthetic complete medium (2% glucose; with uracil).

Strain	Substrate	Concentration	**Glucose release** [nmol glucose µg^{-1} protein min^{-1}] Mean	S.D.
48A	Maltose (DP2)	160 mM	0.27	0.02
199			0.27	0.01
48A	Maltose (DP2)	40 mM	0.13	0.01
199			0.13	0.00
48A	Maltotriose (DP3)	40 mM	0.040	0.003
199			0.042	0.003
48A	Maltotetraose (DP4)	40 mM	0.004	0.001
199			0.005	0.001
48A	Maltopentaose (DP5)	40 mM	0.0025	0.0003
199			0.0013	0.0002
48A	Maltohexaose (DP6)	40 mM	0.0010	0.0002
199			0.000	0.001
48A	Glycogen	17.5 mg ml^{-1}	-0.0011	0.0001
199			-0.0014	0.0002

Table S3. Summary of yeast maltase activity towards different glucan substrates.

Glucose release was calculated from the slopes of the linear regressions shown in **Figs. S6** and **S7**. Note that maltase activity rapidly decreases with increasing degree of polymerization (DP) of the substrate. Given concentrations are starting concentrations of the substrate in the assay. Activities are means with standard deviation (S.D.) from two replicate cultures.

CEN.PK113-7D accession	Standard name	Systematic name	Description (Saccharomyces genome database)	Fold change (29 *vs.* 48A)	*q*-Value
n.a.	glgC-TM	n.a.	*E.coli* ADPglucose pyrophosphorylase	13.76	3.1E-06
N1P0H8_YEASC	CWP1	YKL096W	Cell wall mannoprotein	13.43	7.6E-03
N1P7W2_YEASC	HBT1	YDL223C	Shmoo tip protein, substrate of Hub1p ubiquitin-like protein	10.28	2.3E-02
N1PAM1_YEASC	STL1	YDR536W	Glycerol proton symporter of the plasma membrane	6.72	3.0E-02
N1P249_YEASC	PRM10	YJL108C	Pheromone-regulated protein	5.00	1.2E-02
N1P8D9_YEASC	PHO3	YBR092C	Constitutively expressed acid phosphatase similar to Pho5p	3.73	1.6E-02
N1NVH2_YEASC	VPS28	YPL065W	Component of the ESCRT-I complex	3.39	6.2E-03
N1P3Y8_YEASC	CTT1	YGR088W	Cytosolic catalase T	2.74	7.6E-03
N1P6W4_YEASC	ATG8	YBL078C	Ub-like protein	2.69	4.1E-02
N1P0X3_YEASC	UTH1	YKR042W	Mitochondrial inner membrane protein	2.69	2.9E-02
N1P0S9_YEASC	DIA1	YMR316W	Protein of unknown function	2.55	2.5E-02
N1P8E6_YEASC	GSH1	YJL101C	Gamma glutamylcysteine synthetase	2.53	2.9E-02
N1PAS5_YEASC	YBR085C-A	YBR085C-A	Protein of unknown function	2.50	3.8E-02
N1P0Q3_YEASC	JEN1	YKL217W	Monocarboxylate/proton symporter of the plasma membrane	2.35	4.1E-02
N1P3G9_YEASC	NQM1	YGR043C	Transaldolase of unknown function	2.12	4.0E-02
N1P385_YEASC	CMK1	YFR014C	Calmodulin-dependent protein kinase	2.09	1.7E-02
N1P6H7_YEASC	BIO2	YGR286C	Biotin synthase	2.06	4.8E-03
N1P396_YEASC	YGL039W	YGL039W	Aldehyde reductase	2.06	1.4E-02
N1P674_YEASC	GPD1	YDL022W	NAD-dependent glycerol-3-phosphate dehydrogenase	2.02	2.3E-02
N1P3V6_YEASC	ERG1	YGR175C	Squalene epoxidase	0.43	2.1E-02
N1NYP0_YEASC	FET3	YMR058W	Ferro-O2-oxidoreductase	0.39	1.0E-02
N1P837_YEASC	PLN1	YKR046C	Perilipin, involved in formation and stability of lipid droplets	0.35	1.2E-02

Table S4. Proteins changes between yeast strain 29 and strain 48A measured by shotgun proteomics.

Strain 29 produces insoluble glucans, while strain 48A does not as it does not possess the ADPglucose pyrophosphorylase glgC-TM (**Fig. S1A**). Yeasts were grown as described in **Fig. 1B**, harvested after 3h cultivation in YP-galactose and subjected to total protein extraction, tryptic peptide preparation and label-free shotgun proteomics. Sample size was 4 replicate cultures for each strain. Protein abundances were quantified based on MS1 intensities using ProgenesisQI. Only protein changes that are at least two fold with a *q*-value ≤0.05 are regarded as significant and listed here. In total 2788 endogenous yeast and heterologously expressed proteins were quantified at a false discovery rate of 0.5%, among which 19 were significantly more abundant (in green) and 3 significantly less abundant (in orange). Source data is provided in **Additional file 14**. Gene ontology analysis using the Gene Ontology Consortium database (Carbon et al., 2021; Ashburner, 2000) released 2020-11-01 (doi: 10.5281/zenodo.5725227) did not reveal any biological process, molecular function or cellular component as enriched among the significantly changed proteins.

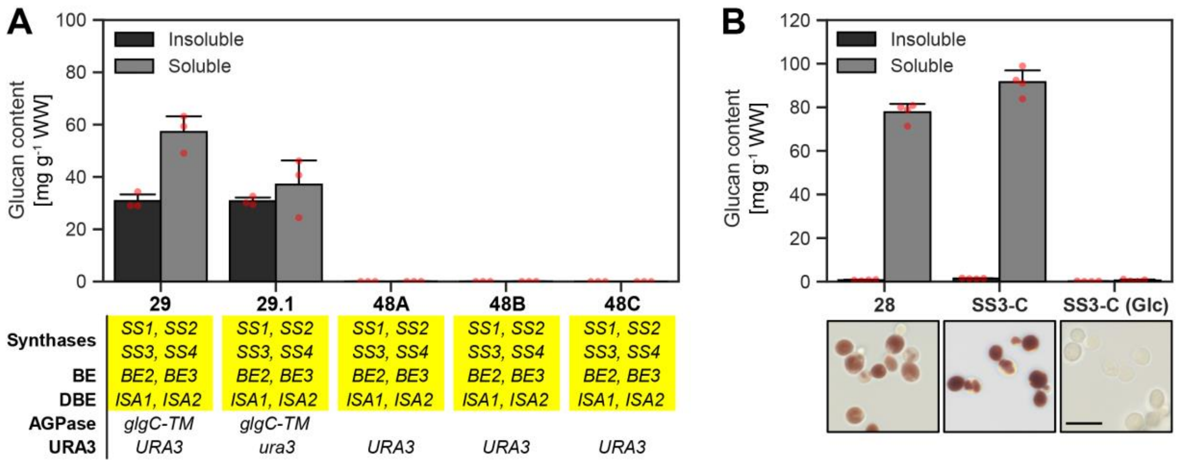

Fig. S1. Control glucan measurements. Yeasts were grown according to the regime shown in **Fig. 1B**, except for SS3-C (Glc), which was inoculated in a main culture of YP-glucose to repress *glgC-TM* expression. Data are means ± S.D. (*n* = 3 replicate cultures in panel **A** and 4 replicate cultures in panel **B**), with the data from each replicate being depicted by red points.

A) Glucan content of yeast strains without glgC-TM, the ADPglucose pyrophosphorylase (AGPase). The relevant genotypes of the strains are depicted below their numbers, with Arabidopsis genes highlighted in yellow. Strain 29.1 was created by selecting cells of strain 29 that had lost the *URA3* marker. This strain served as a progenitor to delete *glgC-TM* in a subsequent transformation, yielding the independent transformants 48A, 48B, and 48C. Strains 48A, 48B and 48C had soluble and insoluble glucan contents <0.1 mg g^{-1} WW (wet weight), indicating that provision of ADPglucose by glgC-TM is a prerequisite for glucan production by the Arabidopsis enzymes. BE, branching enzymes; DBE, debranching enzyme. For numerical data, see **Additional file 4, sheet 3**.

B) Glucan content of yeast SS3-C grown in medium with glucose and strain 28 grown in medium with galactose. The lower panels show light micrographs of iodine-stained cell cultures. The size bar (10 µm) applies to all images. The absence of glucans in SS3-C grown in glucose-containing medium re-confirms that glucan synthesis is dependent on the supply of ADPglucose and that our glucan measurements are specific for α-glucans. Strain 28, which contains several *pGAL* driven *SS* and *BE* genes, was previously shown to accumulate only soluble glucans (Pfister et al., 2016), in-line with our results. For numerical data, see **Additional file 4, sheet 1**.

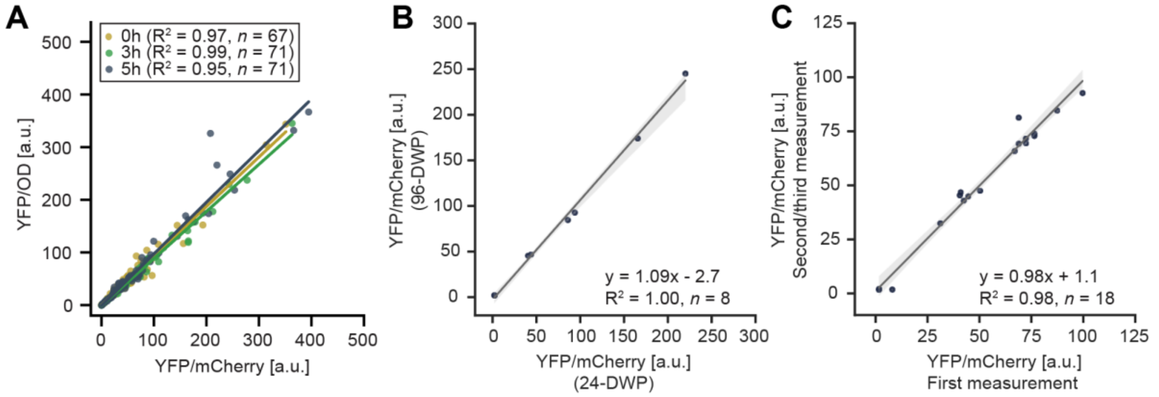

Fig. S2. Quality control of YFP fluorescence measurements.

Yeast cultivation and calculation of the fluorescence parameters were conducted as described in **Fig. 1**. Each data point represents the mean fluorescence of a yeast strain expressing the mCherry and YFP reporters. The light gray shadings around the regression lines in panels **B** and **C** show their 95% confidence intervals. Number of replicates, numerical data and statistics are provided in **Additional file 2**. R^2, coefficient of determination of the linear best-fits; *n*, number of means used in the analysis.

A) Comparison of YFP normalized to OD or mCherry at time point 0h (yellow), 3h (green) and 5h (gray). The high coefficients of determination (R^2) and the similarity of the slopes (difference <10%) of the three time points from the linear regression fits indicate that both normalization methods give comparable results.

B) Effect of culture size on fluorescence. Most main cultures in YP-galactose were grown in 24-deep well plates (DWPs), but we occasionally also used 96-DWPs for main cultures. To test the influence of culture size on fluorescence, replicate cultures of four strains with SS4-YFP (strains 371, 386, 391, and 427) were first grown overnight in a 96-DWP, then inoculated into a 24-DWP in YP-galactose and assayed for YFP and mCherry fluorescence after 3h and 5h. Shown are the YFP/mCherry data from time points 3h and 5h, which cluster together except for the two highest values that derive from *pGAL1*-driven YFP reporters.

C) Effect of measurement date on fluorescence. Replicate cultures of five strains with various YFP reporter constructs (strains 371, 386, 391, 489, and 490) were cultivated and assayed multiple times over the whole measurement period. Shown are the YFP/mCherry data from all three time points.

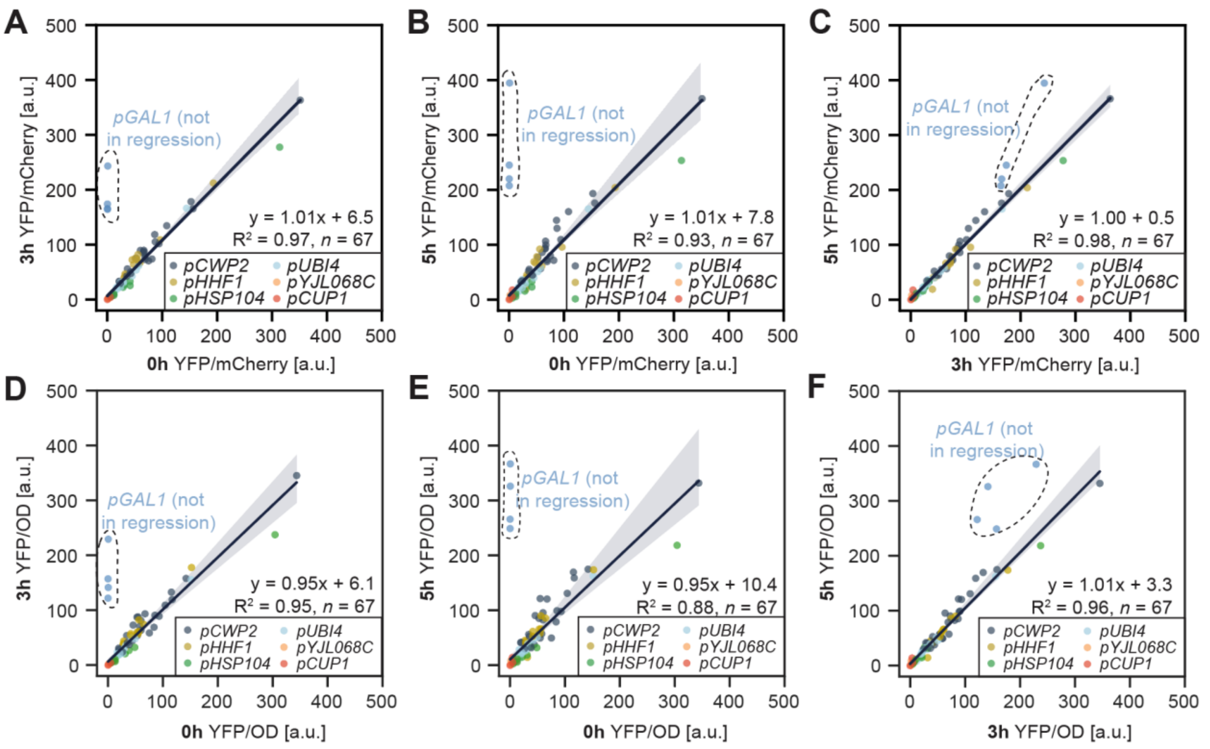

Fig. S3. Stability of normalized YFP fluorescence over the time course.

Yeast cultivation and acquisition of the fluorescence parameters were conducted as described in **Fig. 1**. Each data point represents the mean fluorescence of a yeast strain expressing the mCherry and YFP reporters. The promoters of the YFP constructs are indicated by color. Control strains with a *pGAL1*-driven YFP reporter are not included in the regressions as they are not stably expressed over the time course, as expected. The light gray shadings around the regression lines show their 95% confidence intervals. Numerical data and statistics are provided in **Additional file 2**. R^2, coefficient of determination of the linear best-fits; *n*, number of means used in the analysis.

A-C) Comparison of YFP/mCherry fluorescence at the indicated time points.

D-F) Comparison of YFP/OD fluorescence at the indicated time points.

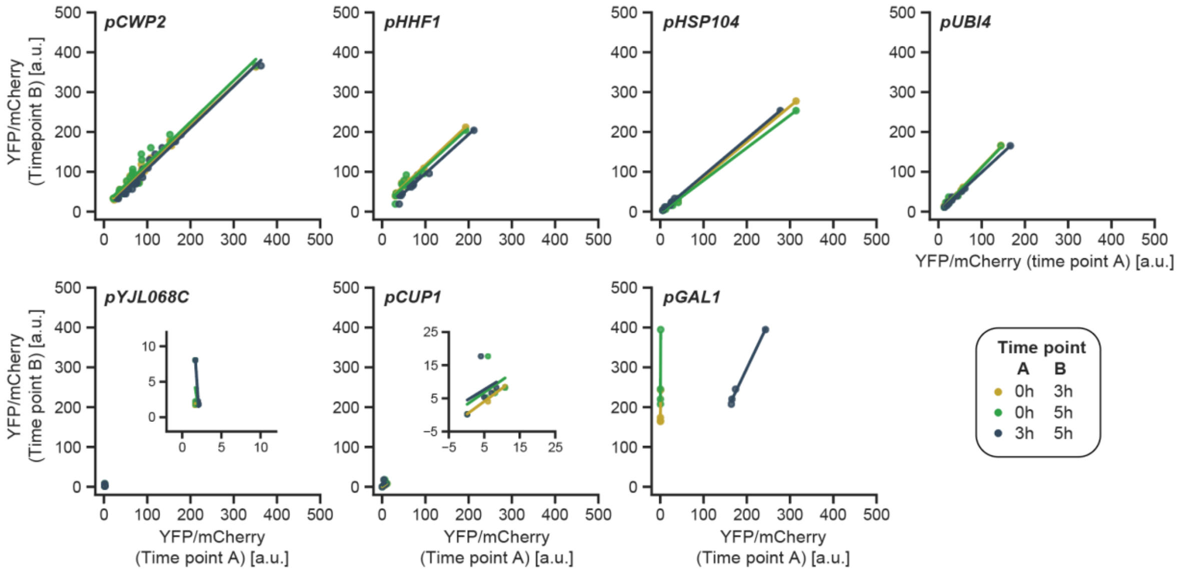

Fig. S4. Stability of expression conferred by different promoters.

Data was replotted from **Figs. S3A-C** to illustrate the change of expression during the time course for each promoter individually. The full equations and statistics of the best-fit linear regressions using the least-ordinary squares model are given in **Additional file 2**.

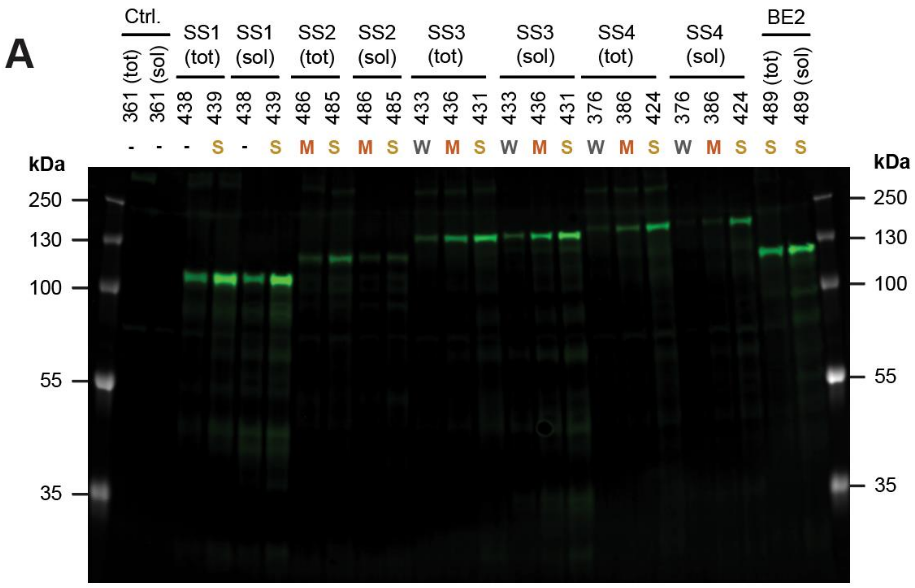

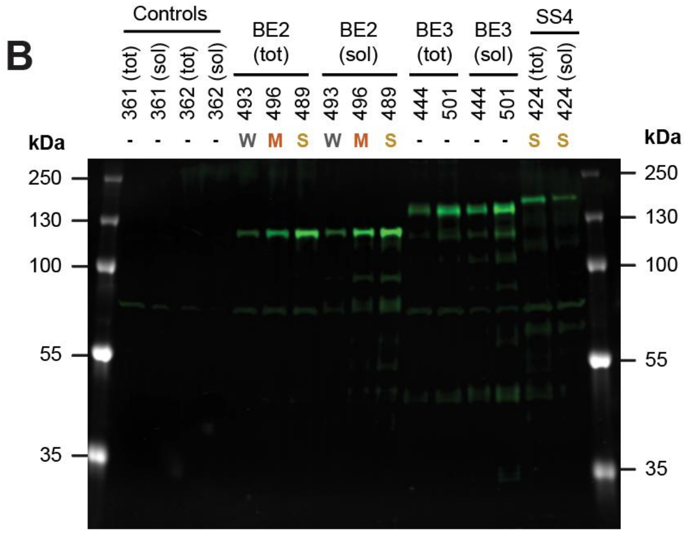

C

Protein	MW [kDa]
SS1-YFP	94
SS2-YFP	109
SS3-YFP	142
SS4-YFP	140
BE2-YFP	115
BE3-YFP	121
YFP	27

Fig. S5. Immunoblots of YFP-tagged proteins expressed in yeast.

A-B) The indicated yeast strains were grown as described in **Fig. 1B** and harvested after 3h in YP-galactose. Cells were homogenized in native extraction buffer (without detergent). Total (tot) proteins were extracted by boiling the homogenates in 2% SDS before centrifugation; these extracts contain both soluble and insoluble proteins. For native soluble (sol) extracts, no detergent or heat was applied to the homogenized cell suspensions before centrifugation – these extracts contain water-soluble proteins. Fifteen µg protein were loaded in each lane. Strains 361 and 362 possess mCherry but no YFP reporter. Where indicated, W (weak), M (medium) and S (strong) refer to the promoter/terminator combinations that were later used to produce strains for glucan biosynthesis. Green signal derives from the anti-GFP antibody, which recognizes YFP, and white signal shows the marker. Note that signal strengths in total and soluble extracts appear similar, indicating that the majority of the proteins of interest are present in a soluble state.

C) The expected molecular weights (MW) of the YFP-tagged protein. Due to the different loading buffer composition of the marker (PageRuler Plus Prestained from Thermo Fisher) compared to the protein samples, the marker bands migrate differently (see also **Fig. S8**). Accordingly, the indicated sizes of the marker bands only approximate the sizes of the sample proteins.

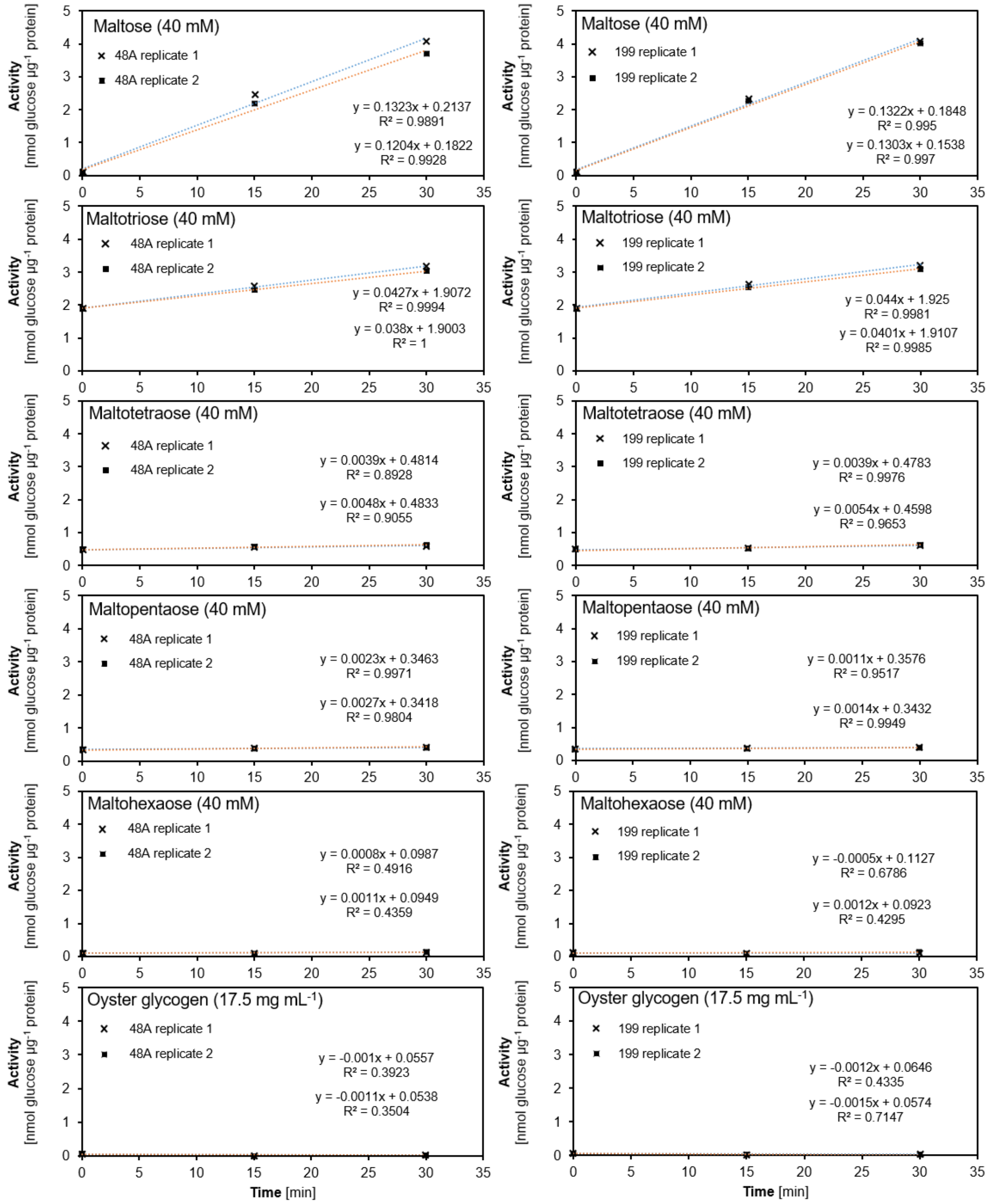

Fig. S6. Maltase activity towards different glucan substrates.

Crude native protein extracts were assayed for glucose-releasing activity towards the indicated substrates after 15 and 30 min incubation at 28°C. Strains 48A (described in **Fig. S1**) and strain 199 *(MATa MAL2-8C SUC2 his3Δ gdb1Δ gph1Δ*) have deletions *GDB1* (encoding for glycogen debranching enzyme) and *GPH1* (encoding glycogen phosphorylase) genes to disable any glucose release by these enzymes. Assays were conducted in duplicates (*n* = 2 replicate cultures).
Dotted lines show the best-fit linear regressions of the data, with the blue lines and upper equations corresponding to biological replicates 1, and the red lines and lower equations to replicate 2. The relatively high levels of glucose measured at time point 0 min when using maltotriose as substrate probably is caused by its low purity of 90% (according to its manufacturer), rendering a contamination with glucose (and possibly also maltose) likely. Activities calculated from the slopes of the linear regressions are summarized in **Table S3**. For numerical data, see **Additional file 3**. R^2, coefficient of determination.

A

Name of target guide RNA	Target guide RNA (5'-3')	Distance of introduced stop codon from ATG start codon	No growth on YP-maltose	No amplification of wild-type MALx2 allele(s)	Correct gene editing confirmed by sequencing	Positive clone(s) used for activity assays
A-MALx2	GATGGCTGGGGTGATTTAAA	105 bp	5 out of 8	2 out of 8	1 out of 8	504
B-MALx2	TGGAGTCTTTAAAACTTGCT	69 bp	5 out of 8	3 out of 8	2 out of 8	505A, 505B
C-MALx2	ATCTTTAATATACTGCAACT	126 bp	5 out of 8	1 out of 8	0 out of 8	none

B

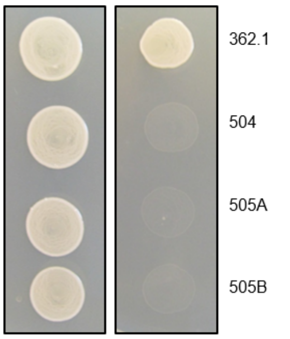

C

Strain	Replicate	Slope of linear fit	Intercept of linear fit	R^2 of linear fit	Glucose release [nmol glucose µg^{-1} protein min^{-1}] Mean (of slope)	S.D. (of slope)
48A	1	0.2843	0.3124	0.9999	**0.27**	**0.02**
	2	0.2493	0.3482	0.9993		
199	1	0.2759	0.3415	0.9997	**0.27**	**0.01**
	2	0.2606	0.3555	0.9991		
362.1	1	0.2768	0.174	0.9961	**0.278**	**0.002**
	2	0.2801	0.342	0.9999		
504	1	0.0001	0.3534	0.0961	**0.0004**	**0.0004**
	2	0.0006	0.3432	0.0587		
505A	1	0.0013	0.3264	0.4197	**0.0016**	**0.0004**
	2	0.0019	0.315	0.9079		
505B	1	0.0005	0.341	0.9204	**0.0009**	**0.0006**
	2	0.0013	0.3385	0.9989		

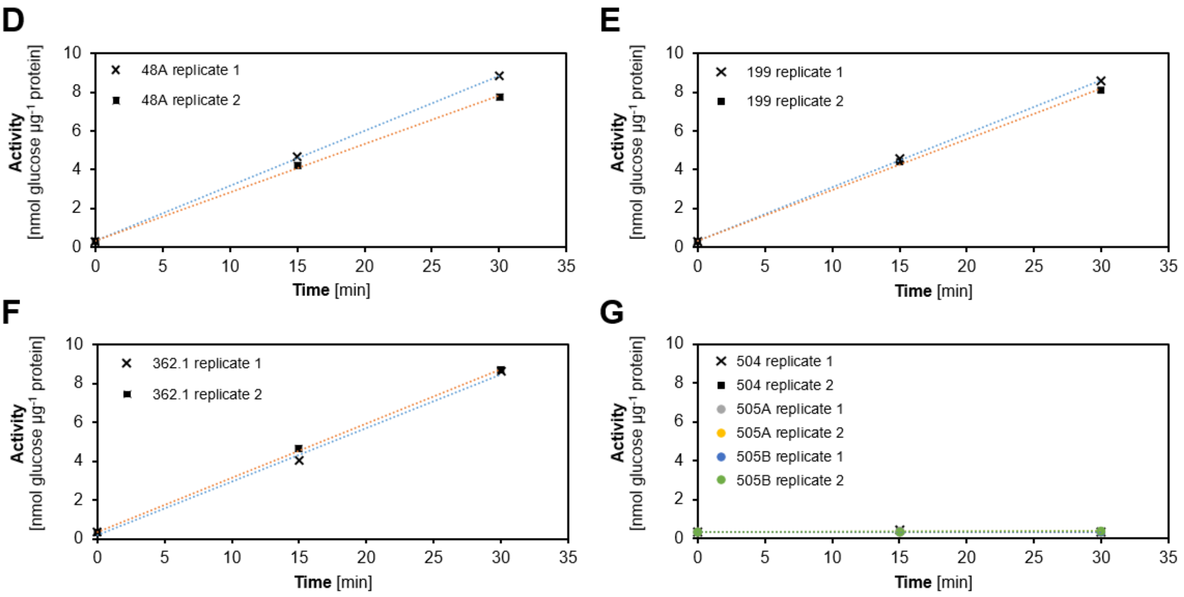

Fig. S7. Gene editing of all *MALx2* genes results in loss of maltase activity.

A) *MALx2* genes were edited by transforming three different guide RNA sequences in a CRISPR/Cas9 expression construct together with repair DNA into yeast strain 362.1. The repair DNA was designed to introduce a stop codon at the indicated position followed by a unique barcode for PCR (for detailed information on constructs and primers for genotyping, see **Additional file 10, sheet 5**). Each guide RNA matches the sequence of the three available *MALx2* gene sequences (*MAL12, MAL22* and *MAL32*) from CEN.PK113-7D, and most likely also that of *MAL42*, whose sequence is not available as the high similarity between the *MAL4* and *MAL2* loci impeded locus reconstruction (Nijkamp et al., 2012). Out of the 8 tested transformants for each edit, 1 (for A-MALx2) or 2 transformants (for B-MALx2) were regarded as successfully edited, as they failed to grow on medium with maltose as sole carbon source (**B**), showed correct gene editing in Sanger sequencing of maltase genes and did not allow any amplification of non-edited *MALx2* alleles in multiple PCRs.

B) Loss of capability of yeast with gene edits in *MALx2* genes to grow on medium with maltose as sole carbon source. Yeast cells amplified on a YPD plate were resuspended in water, dropped onto plates with YP containing 2% [w/v] glucose (YPD) or maltose (YP-maltose) as carbon source, incubated at 30°C for 16 h and photographed. Strain 362.1 was subjected to gene editing and 504,

505A, and 505B are the three resulting clones that passed all tests for gene editing described in panel **A**.

C) Absence of maltase activity in gene-edited strains. Glucose-releasing activities of maltases were assayed in duplicates as described in **Fig. S6** but using 160 mM maltose as substrate (*n* = 2 replicate cultures). Due to the low affinity of maltases against their substrates (K_m ~ 80 mM for maltose), maltase activities towards 160 mM are considerably higher than towards 40 mM maltose (**Fig. S6**; all data shown in **Figs. S6** and **S7** were acquired together). R^2, coefficient of determination;

D-G) Data underlying the activity calculations presented in **C**. Dotted lines show the best-fit linear regressions of the data, with the blue and lines corresponding to biological replicates 1 and 2, respectively. For numerical data, see **Additional file 3**.

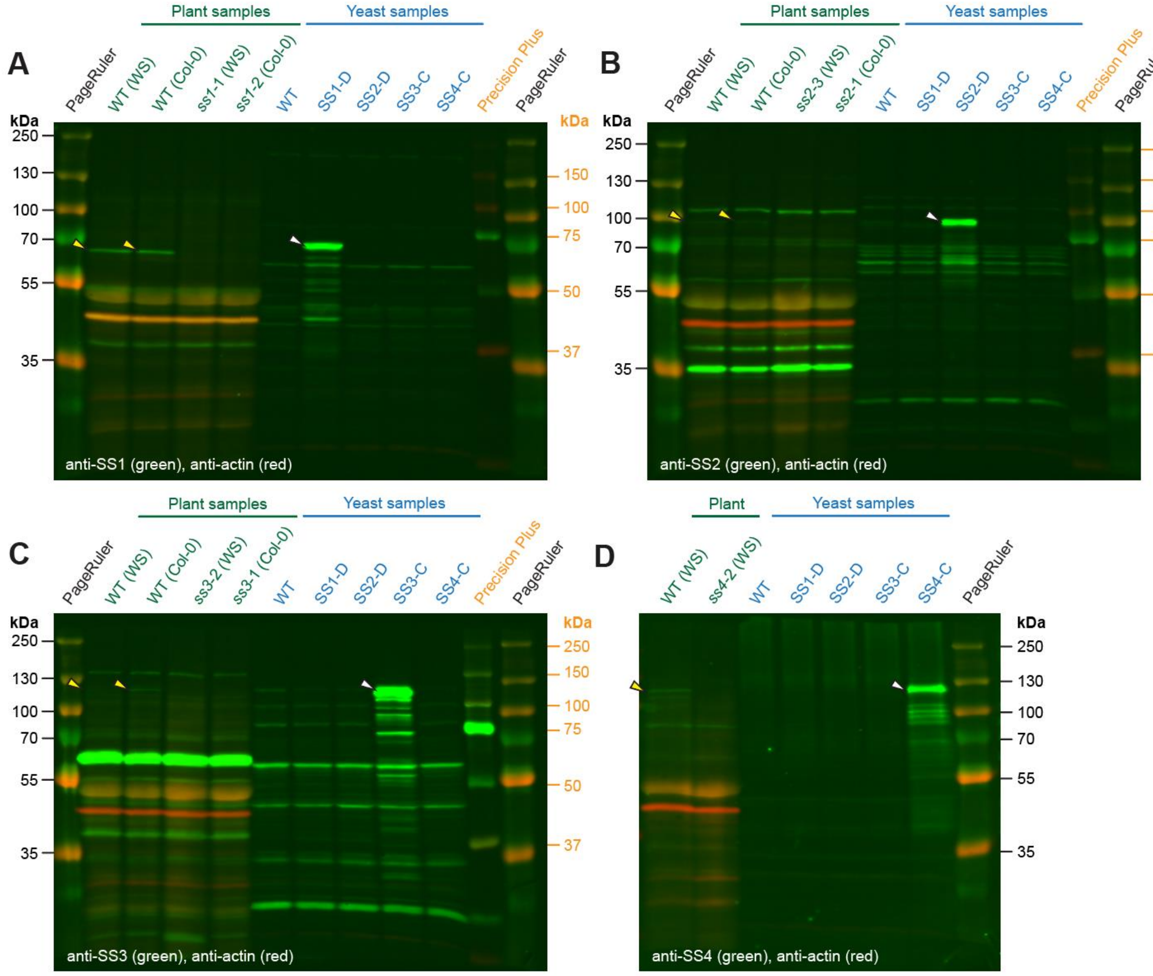

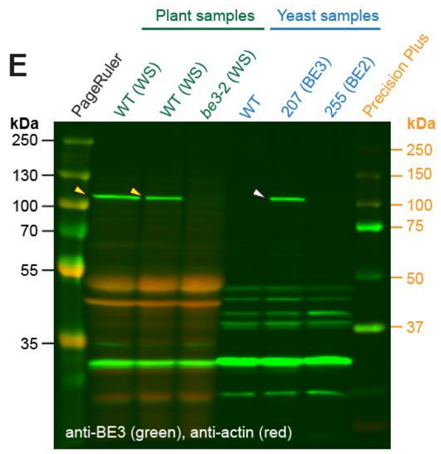

F

Protein	MW [kDa]
SS1	67
SS2	82
SS3	115
SS4	113
BE3	94

Fig. S8. Western blots of untagged SS and BE proteins.

Total proteins (using SDS buffer) were extracted from leaf disks of homozygous *Arabidopsis thaliana* lines (harvested during the middle of a normal day with a 12h photoperiod) or from yeast cultures cultivated for 3h in YP-galactose according to the growth regime shown in **Fig. 1B**. Proteins were loaded according to equal input leaf area (plant samples) or protein amount (yeast samples). Blots were probed with the anti-SS4 antibody (**D**) (Roldán et al., 2007) or newly raised polyclonal affinity-purified antibodies against SS1 (**A**), SS2 (**B**), SS3 (**C**) and BE3 (**E**). The signals from these antibodies were visualized in the green channel, while those from the anti-actin antibody – which recognizes plant β-actin and serves as loading control of plant samples –, were detected in the red channel. Strains

207 and 255 contain BE3 and BE2 as sole branching enzyme, respectively (refer to **Additional file 9** for full information on plant alleles and yeast genotypes). The expected molecular weights (MWs) of the endogenous Arabidopsis proteins (less their chloroplast transit peptides) or when expressed in yeast are provided in panel **F**. The Arabidopsis and yeast protein bands corresponding to proteins of interest are indicated by yellow and white arrows, respectively. Marker bands derive from Precision Plus Protein WesternC (Bio-Rad; MWs shown in orange) or PageRuler Plus Prestained (Thermo Fisher; MWs shown in black). Whereas Precision Plus is provided in a loading buffer similar to that of our protein samples, PageRuler uses a different buffer system which renders estimation of protein sizes less accurate. WS, Wassilewskija ecotype; Col-0, Columbia-0 ecotype.

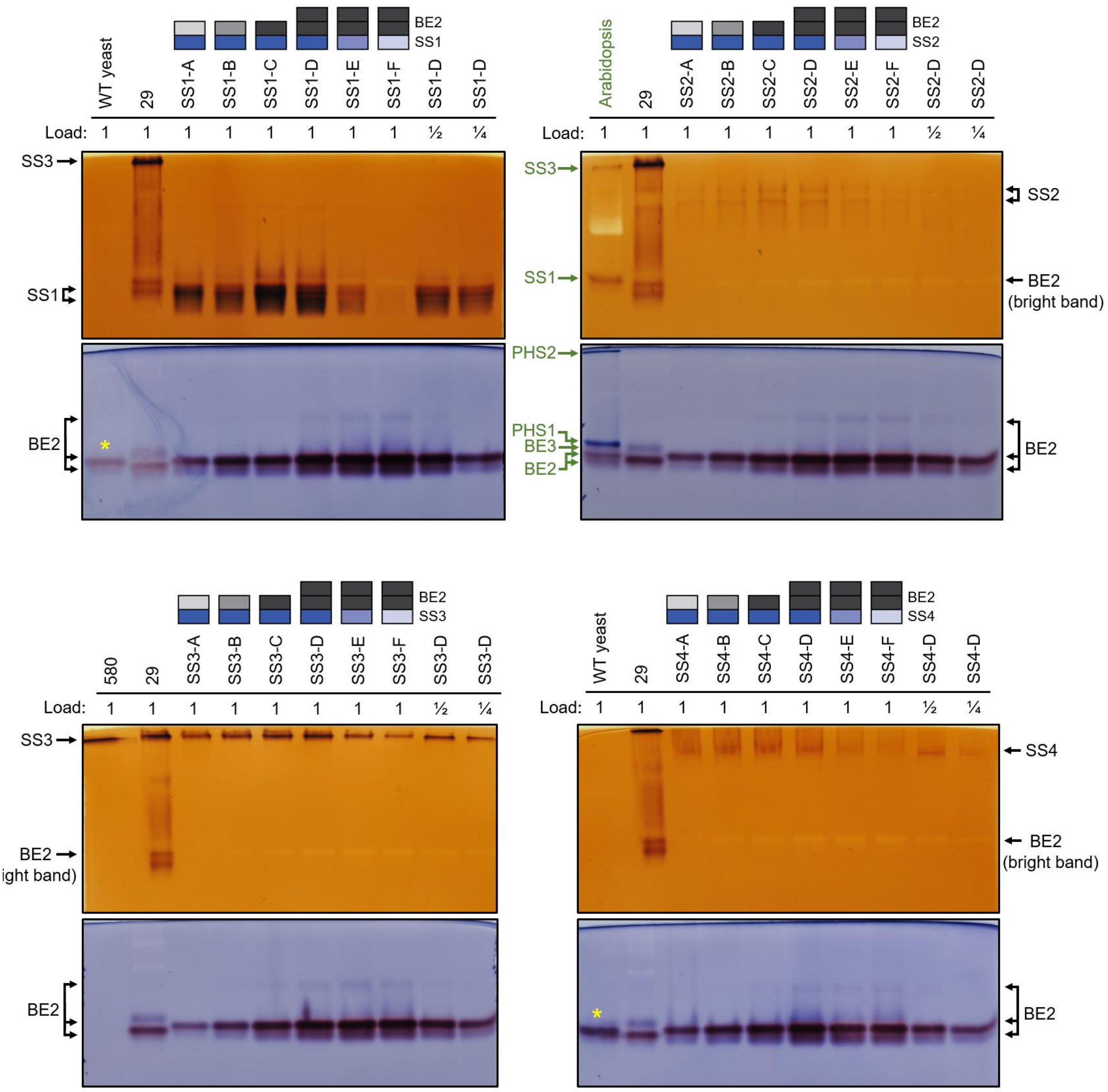

Fig. S9. Native PAGE monitoring the activities of starch synthases (SSs) and branching enzymes (BEs) in yeast.

Soluble native proteins were extracted from the indicated yeast strains (grown as in **Fig. 1B;** harvested after 3h cultivation in YP-galactose) and leaves from wild-type (WT) *Arabidopsis thaliana* plants. The anticipated expression strengths of BE2 and the SS are indicated above the gels. Arabidopsis enzyme activities (in green) were deduced from earlier mutant analysis (e.g. summarized in Supplemental Fig. S1 in Pfister et al., 2014). The BE activity observed in WT yeast (indicated by an asterisk) is Glc3p. Strain 580 does not contain a BE but is otherwise isogenic to SS3-A. Differences in migration of activity bands between strain 29 (Pfister et al., 2016) and other strains are likely due to subtle differences in constructs. "Load" indicates protein amount relative to other samples. PHS1, plastidial phosphorylase; PHS2, cytosolic phosphorylase.

Upper panels (SS activity gels): Gels containing 0.015% glycogen as substrate were incubated with ADPglucose to allow elongation of glycogen chains by SSs and stained with Lugol's solution to visualize glucan products. Highly abundant BE2 results in a bright band, presumably due to excessive branching of glycogen, reducing its affinity for iodine.

Lower panels (BE activity gels): Gels containing 0.3% glycogen as substrate were incubated with phosphorylase and glucose 1-phosphate and stained with Lugol's solution to visualize glucan products. Since BE activity creates branches, it provides new substrates for chain elongation by phosphorylase, resulting in dark-staining bands.

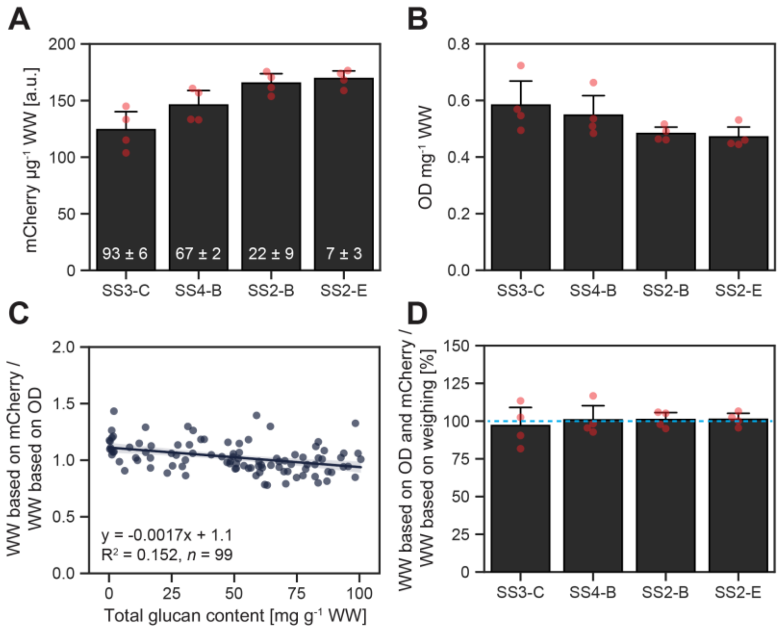

Fig. S10. Relationships between yeast mCherry fluorescence, optical density (OD) and wet weight (WW).

Relationships were established using multiple strains covering a broad range of glucan contents to account for potential effects of glucan accumulation on OD and/or mCherry measurements. Cultures of sufficient size to allow reliable weighing of the cell pellets were grown as described in **Fig. 1B**, harvested after 5.75h cultivation in YP-galactose, washed and their WWs determined by weighing the fresh cell pellet. Aliquots of the cell suspensions were subjected to mCherry and OD measurements in a plate reader to test the usability of the mCherry signal intensity and OD for estimating yeast WWs. Shown are means ± S.D. (n = 4 replicate cultures). The data shown in panel **C** derives from samples used for glucan quantifications presented in **Fig. 4**. Source data is provided in **Additional file 4**.

A) mCherry signal per µg cells (WW) present in a plate reader well. Numbers in white indicate the total glucan content ± S.D. [mg g^{-1} WW] of these strains (values taken from **Fig. 4**). Strains that accumulate more glucans tend to give lower mCherry fluorescence, presumably due to quenching of fluorescence by glucans. In average, one µg cells (WW) dispersed in a plate reader well gave an mCherry signal of 151 ± 22 (mean ± S.D.; n = 16 replicate cultures).

B) OD per mg cells (WW) present in a plate reader well, using the same cell suspensions as in panel **A**. Strains with higher glucan content had a higher optical density per mg cells, probably because the glucans also absorb light. In average, 1 mg cells (WW) in a plate reader well had an OD of 0.51 ± 0.08 (mean ± S.D.; n = 16 replicate cultures).

C) Influence of glucan content on mCherry signals and OD. The WWs of cell suspensions were calculated either based on mCherry signal or OD (using the average values stated in panels **A** and **B**, respectively). The ratio between these two values was then plotted against total glucan content. Data derives from the individual replicates used for glucan quantifications presented in **Fig. 4**. The WW used for glucan quantification on the x-axis was calculated based on the average of WW based on mCherry and based on OD. There is a weak trend towards underestimating WW based on mCherry and/or overestimating WW based on OD with increasing glucan content. The full statistics of the regression analysis are given in **Additional file 4, sheet 5**.

D) WW based on combining mCherry and OD measurements (by averaging them) relative to the WW determined by weighing of the cell pellet. All ratios are very close to 100% (indicated by a dashed blue line), suggesting that combining mCherry signal and OD reliably estimates the WW irrespective of glucan content. This approach was used for the glucan quantifications presented in **Fig. 4**.

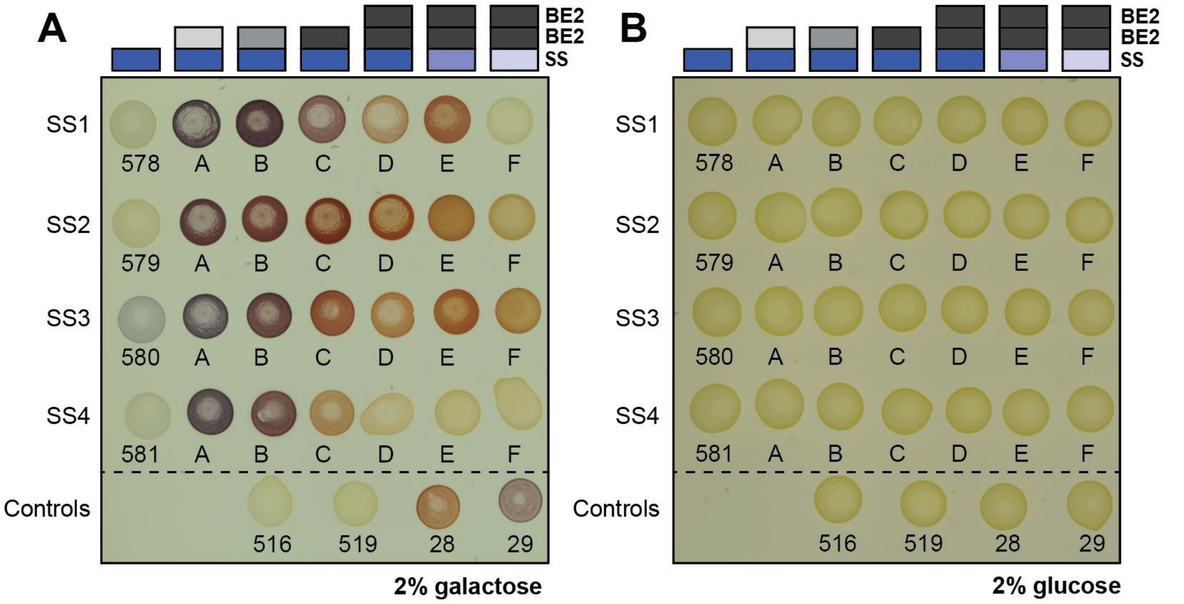

Fig. S11. Iodine staining of cell patches grown on galactose- or glucose-containing plates.

The indicated yeast strains were grown for 24h on SC-plates with 2% galactose (**A**) or 2% glucose (**B**), stained for glucans with iodine vapor from I_2/KI solution and photographed. Strains 578, 579, 580 and 581 do not express any BE, but are otherwise isogenic to strains A from the same set. Strains 516 and 519 are progenitor strains containing BE2 but no SS (see **Additional file 9, sheet 1** for a list of genotypes). Excerpts of these photographs were shown in **Fig. 4**.

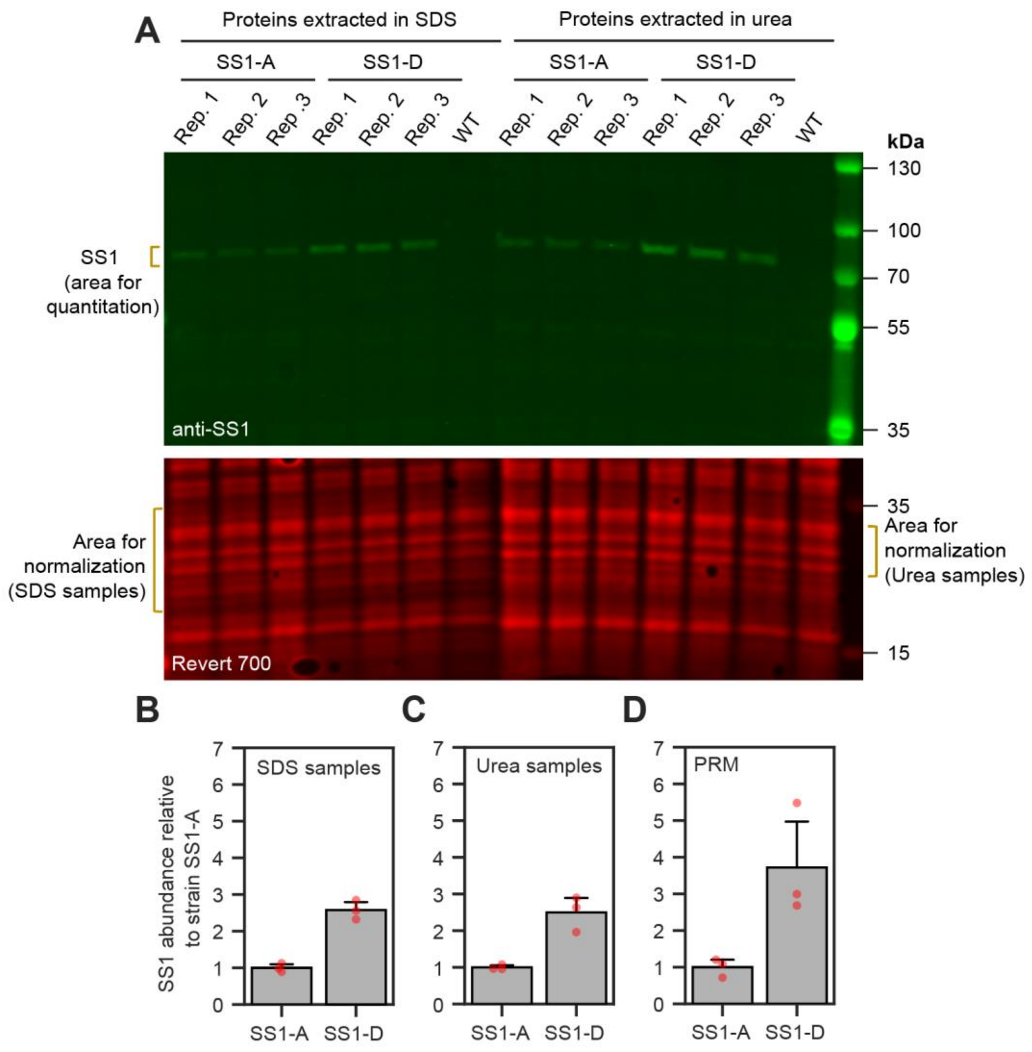

Fig. S12. Quantification of SS1 abundance after different extraction methods by Western blotting.

Three replicate (Rep.) cultures of yeast strains SS1-A and SS1-D and a culture of WT yeast (negative control) were cultivated as described in **Fig. 1B**. After 3h growth in YP-galactose, cells from each culture were pelleted, washed and split into two halves. One half was harvested and further processed for total protein extraction in 2% sodium dodecyl sulfate (SDS), a standard procedure for complete protein solubilization. The other half was harvested and processed in 8M urea buffer as for protein extraction for proteomics. Numerical data is provided in **Additional file 7, sheet 1**.

A) Anti-SS1 Western blots. Fifteen µg of protein were loaded in each lane. Prior to probing with the anti-SS1 antibody (upper panel), total proteins on membrane were reversibly stained by Revert 700 and detected by fluorescent signal (lower panel). The areas that were used to quantify SS1 (upper panel) and to normalize for protein load (lower panel) are indicated with orange brackets. A smaller area for normalization of the samples extracted in urea was chosen to avoid areas with imperfect protein transfer due to air bubbles. The calibration curves to assess the linearity of the quantification are presented in **Fig. S13**. For the validation of the SS1 antibody, see **Fig. S8A**. The expected molecular weight of SS1 is 67 kDa.

B) Quantification of SS1 after protein extraction in SDS from the blots presented in panel **A**. The signal from SS1 was normalized to total protein on membrane and expressed relative to SS1

abundance of strain SS1-A. Shown are means ± S.D. from three replicate cultures, with red points showing the individual measurements.

C) As in panel **B**, but showing the samples after protein extraction in urea.

D) SS1 abundance relative to strain SS1-A as determined by parallel reaction monitoring (PRM; data was re-calculated from **Fig. 6**). Proteins were extracted as in panel **C**. Shown are means ± S.D. from three replicate cultures, with red points showing the individual measurements.

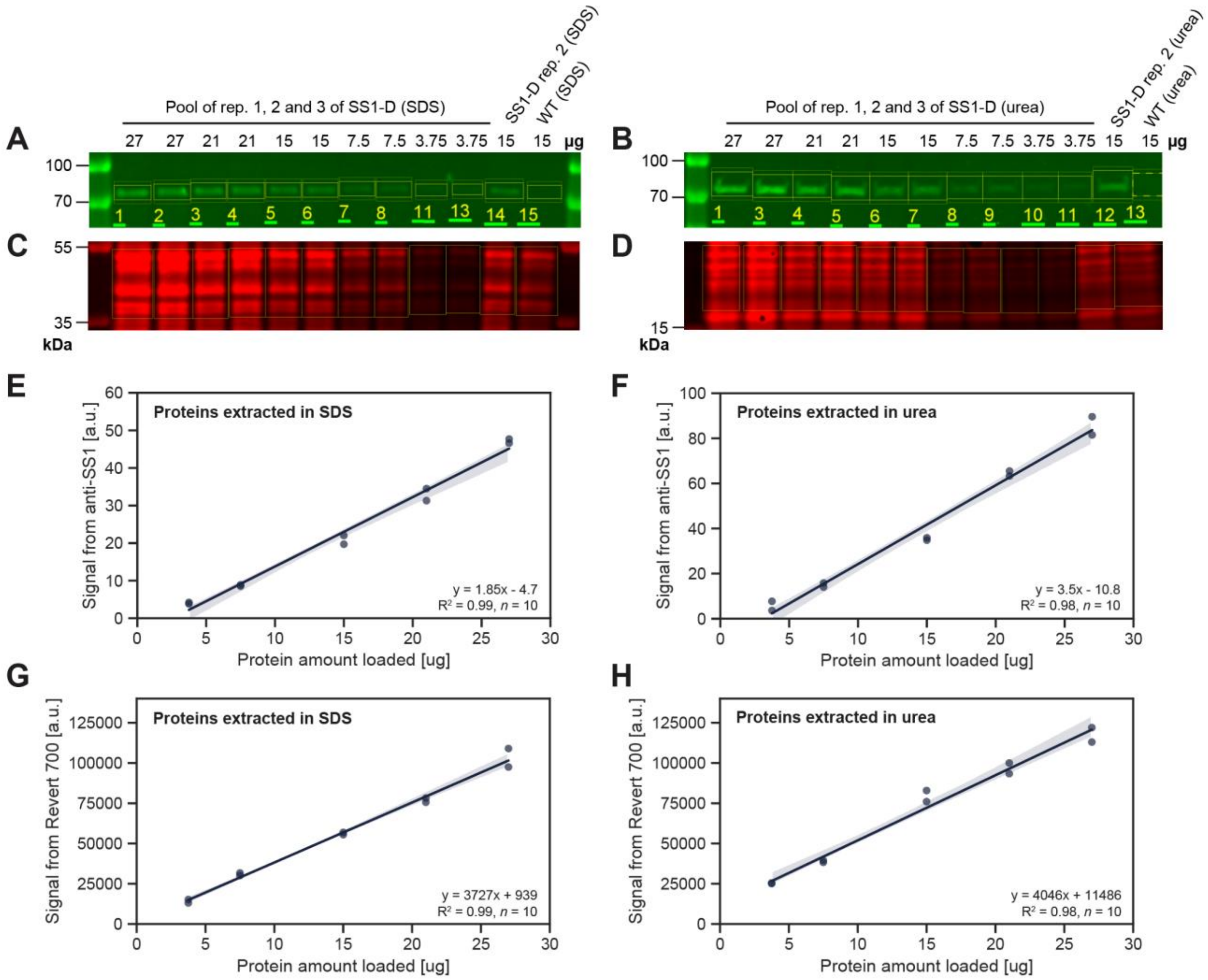

Fig. S13. Assessment of linear range for quantification of SS1 by Western blotting.

Pooled samples of SS1-D (described in **Fig. S12**) that were either extracted in 2% sodium dodecyl sulfate SDS (left panels) or 8M urea buffer (right panels) were subjected to immunoblotting using the anti-SS1 antibody. Prior to probing with the anti-SS1 antibody, the membrane was reversibly stained with Revert 700 for quantification of protein transferred onto the membrane. SS1-D replicate 2 and WT protein extracts are internal and negative controls, respectively. Marker bands derive from PageRuler Plus Prestained (Thermo Fisher). All panels derive from the same blot. Numerical data and the statistics of the regression analyses are presented in **Additional file 7**.

A-B) Quantification of the signal from the anti-SS1 antibody obtained from protein extracts in prepared in SDS (**A**) or urea (**B**). The expected protein load in µg based on upfront protein quantification is given on top. The areas used for quantification of SS1 are indicated by yellow boxes, with the yellow numbers referring to the box numbers used in **Additional file 7, sheet 2**.

C-D) As panels **A** and **B**, but showing the signal from total protein stain of Revert 700. The box numbers from panels **A** and **B** also apply to these. Different areas for quantification were chosen in panels **C** and **D** to avoid areas with imperfect protein transfer due to air bubbles.

E-F) Linear regressions (best fit) of the quantification of SS1, using the data obtained in panels **A** and **B**. The grey shadings indicate the 95% confidence interval of the slope. R^2, coefficient of determination; *n*, number of technical replicates.

G-H) As panels **E** and **F**, but showing the linear regressions (best fit) of the quantification of total protein on the membrane using the data obtained in panels **C** and **D**. R^2, coefficient of determination; *n*, number of technical replicates.

References:

Ashburner, M. (2000). Gene Ontology : tool for the unification of biology. Nat. Genet. **25**: 25–29.
Carbon, S. et al. (2021). The Gene Ontology resource: Enriching a GOld mine. Nucleic Acids Res. **49**: D325–D334.
Keren, L. et al. (2013). Promoters maintain their relative activity levels under different growth conditions. Mol. Syst. Biol. **9**: 701.
Nijkamp, J.F. et al. (2012). De novo sequencing, assembly and analysis of the genome of the laboratory strain Saccharomyces cerevisiae CEN.PK113-7D, a model for modern industrial biotechnology. Microb. Cell Fact. **11**: 36.
Pfister, B., Lu, K.-J., Eicke, S., Feil, R., Lunn, J.E., Streb, S., and Zeeman, S.C. (2014). Genetic evidence that chain length and branch point distributions are linked determinants of starch granule formation in Arabidopsis. Plant Physiol. **165**: 1457–1474.
Pfister, B., Sánchez-Ferrer, A., Diaz, A., Lu, K., Otto, C., Holler, M., Shaik, F.R., Meier, F., Mezzenga, R., and Zeeman, S.C. (2016). Recreating the synthesis of starch granules in yeast. Elife **5**: 1–29.
Roldán, I., Wattebled, F., Lucas, M.M., Delvallé, D., Planchot, V., Jiménez, S., Pérez, R., Ball, S., D'Hulst, C., and Mérida, A. (2007). The phenotype of soluble starch synthase IV defective mutants of *Arabidopsis thaliana* suggests a novel function of elongation enzymes in the control of starch granule formation. Plant J. **49**: 492–504.
Yamanishi, M., Ito, Y., Kintaka, R., Imamura, C., Katahira, S., Ikeuchi, A., Moriya, H., and Matsuyama, T. (2013). A genome-wide activity assessment of terminator regions in *Saccharomyces cerevisiae* provides a "terminatome" toolbox. ACS Synth. Biol. **2**: 337–347.
