# Supplementary material for: Tuning heterologous glucan biosynthesis in yeast to understand and exploit plant starch diversity
Source: BMC Biol. 2022 Sep 24;20:207. doi: 10.1186/s12915-022-01408-x (PMC9509603; doi:10.1186/s12915-022-01408-x)
Supplement: Supplementary file 11 — Additional file 11. Detailed methods of targeted proteomics. [file 12915_2022_1408_MOESM11_ESM.pdf]

Additional File 11. Detailed methods of targeted proteomics

Parallel reaction monitoring (PRM) assays aimed to quantify the relative abundance of the proteins heterologously expressed during glucan synthesis, of proteins that may become relevant in follow-up research, and of endogenous yeast proteins that could serve as housekeeping proteins (**Additional file 6, sheet 3**). The latter had been identified by label-free proteomics (**Additional files 13** and **14**), but their quantification is not relevant for this study, since data calibration was supported using crude stable isotope labeled (SIL) peptides. All strains created for glucan analysis in this study (SS1-A to SS1-F, SS2-A to SS2-F, SS3-A to SS3-F and SS4-A to SS4-F), strain 28 (as positive control) and wild type (as negative control) were subject to PRM analysis.

Selection of surrogate peptides

For each of the proteins of interest (**Additional file 6, sheet 3**), three or four surrogate peptides were selected in Skyline v20.1 (MacCoss Lab Software; (MacLean et al., 2010)). Peptide selection criteria were as follows: 1) uniqueness of the peptide within the proteomes of CEN.PK113-7D yeast (UniProt identifier: UP000013192) and *Arabidopsis thaliana* cv. Columbia (UniProt identifier: UP000006548); 2) a high signal intensity of the precursor and of at least four y-ions in the shotgun MS data from the label-free experiment (Additional file 7); 3) absence of missed cleavages, methionine and N-terminal glutamate or N-terminal glutamine; and 4) absence of reported post-translational phosphorylation or acetylation events in Arabidopsis proteomes (PhosPhAt 4.0, Durek et al., 2009; Uhrig et al., 2019). One surrogate peptide of SS1 (THALDTGEAVNVLK) had been identified as phosphorylated (Roitinger et al., 2015) but was still included; it is unlikely that this phosphorylation occurs when SS1 is heterologously expressed in yeast and its abundance correlated well with the other SS1 peptides (correlation coefficient $\geq$0.97).

Selected surrogate peptides (**Additional file 6, sheet 1**) were ordered with heavy-labelled C-terminal $^{13}C_6{}^{15}N_2$ lysine (+ 8 Da) or $^{13}C_6{}^{15}N_4$ arginine (+10 Da) and carbamidomethyl-modified cysteines (SpikeTides_L, JPT Peptide Technologies, Schnatbaum et al., 2011). Peptides were resuspended in 3% (v/v) acetonitrile, 0.1% (v/v) formic acid according to manufacturer’s instructions, pooled and the resulting peptide mix stored in aliquots at -20°C. The purity of the peptide mix and presence of all labelled peptides was confirmed by shotgun proteomics.

Protein extraction and preparation of tryptic peptides

Three replicates, each derived from an independent yeast culture starting from the pre-culture in YPD, were prepared for each strain. During yeast growth and peptide preparation, yeasts were grouped according to replicate number (not according to strain) in a non-random fashion. Yeasts were grown in DWPs, harvested after 3 h cultivation in YP-galactose by centrifugation at 3,000 *g* for 3 min at 4°C, the pellets washed twice with cold water and resuspended in cold water. Quenching of cellular activities by the addition of trichloroacetic acid, extraction of proteins in urea buffer and peptide preparation using trypsin was essentially conducted as described by Soste et al. (2014), using sequencing grade modified trypsin (Promega) at a trypsin to protein ratio of 1:80 (w/w) for 16 h.

Tryptic digestion was quenched by adding trifluoroacetic acid (TFA) to reach a pH $\leq$ 3 and the samples cleared by centrifugation at 3,000 *g* for 5-10 min to remove urea precipitates, if any. Peptides were desalted on Sep-Pak tC18 cartridges (100 mg absorbent, 1 cc, 37-55 µm particle size, from Waters) using a QIAvac 24 Plus vacuum manifold (Qiagen). Columns were first wetted with 1 ml 100% methanol and then equilibrated with 1 ml 80% (v/v) acetonitrile, 0.1% (v/v) TFA and subsequently with 2 ml 0.1% (v/v) TFA. The acidified peptides were loaded onto the cartridges, the cartridges washed with 3 ml 0.1% (v/v) TFA and the peptides eluted with 800 µl 80% (v/v) acetonitrile, 0.1% (v/v) TFA. Eluted peptides were flash-frozen in liquid nitrogen, dried in a vacuum centrifuge and stored at -20°C. For MS analysis, peptides were resuspended in 3% (v/v) acetonitrile, 0.1% (v/v) formic acid by vortexing and sonication. Peptides were prepared in the same way from

strains 29 and 587 to create a sample matrix for establishing the calibration curves and for acquiring data for a spectral library. Strain 587 (*MATa MAL2-8C SUC2 his3Δ HygR KanR malx2 glc3Δ gsy2Δ glg1Δ glg2Δ gsy1::pGAL1-glgC-TM-HA-tCYC1 bar1Δ XII-2::pCWP2-mCherry-tUPT7 XII-5::pCWP2-BE2-tRPL3-pCWP2-BE2-tCYC1)* is an advanced strain that expresses a similar set of genes as 29 but also contains mCherry.

<u>Determination of linear dynamic range for quantitation</u>

A reverse calibration curve for each SIL peptide was established by running a 6-point serial dilution series of the SIL peptide mix in a constant matrix of peptides derived from yeast strains 29 and 587 including iRT peptides (Biognosys). The dilution series started from ~1-5 pmol (estimated from the targeted synthesized amount) of each SIL peptide on column, followed by five 10-fold dilutions (from undiluted to a 10^{-5} dilution) and blanks containing only matrix but no SIL peptides, using 2-3 technical replicates (see **Additional file 6, sheet 5** for the list of runs). The dilution series data was acquired on the same LC-MS instrument and with an essentially same scheduled PRM method as for the glucan-producing samples (described below). A spectral library (consisting of 13072020Prm_2 and 13072020_PRM_part2) was created from strains 29 and 587 including iRT peptides and the SIL peptide mix at a dilution of 10^{-1} by shotgun proteomics on the same LC-MS device, followed by searching the Mascot generic file (generated with Proteome Discoverer 2.1, Thermo Fisher) with Mascot Server version 2.6 against a custom forward and reverse (decoy) database (containing the CEN.PK113-7D proteome, all heterologously expressed proteins, common MS contaminants and iRT peptide [Biognosys] sequences)

Reverse calibration curves were fitted in Skyline v20.1 and v20.2.0343 (MacCoss Lab Software) setting heavy as isotope label type [isotope modifications: 13C(6)15N(2) (C-term K) and 13C(6)15N(4) (C-term R)] and light as internal standard type. Transitions (y-ions) for quantification were automatically selected based on intensity of library spectra (ion match tolerance: 0.5 m/z), setting the maximum to five transitions and filtering for y-ions that 1) derived from tryptic precursors with charge +2 or +3 with an exclusion window of 2 m/z and without missed cleavage; 2) had a charge of +1; 3) constituted ion 3 to the last ion with the inclusion of N-terminal to proline as special ion; and 4) had an m/z ratio of between 50 and 1,500. Mass accuracies were set to 10 ppm for both MS1 and MS/MS filtering. Accordingly, 4 or 5 y-ions were quantified for each peptide, with the exception of peptide BE3_2 where only 3 y-ions passed the filters (transitions are listed in **Additional file 6, sheet 6**).

Peak assignment and selection of integration borders was performed automatically by Skyline software but manually inspected and modified if needed to reduce the effect of interferences. Regressions were fitted linearly in log space using the peak areas of the transitions selected for quantification (using Tukey's median polish as summary method) without any normalization method or regression weighting. Detailed methods, the calibration curves and derived figures of merit (lower limits of quantitation and detection, slopes and coefficients of determination) of the regressions are provided in **Additional file 12**. The results from the regressions were used to prepare an adjusted pooled standard peptide mix in which the abundances of the SIL peptides approximately matched those of the light peptides. Lower limits of quantitation from the regression analysis were calculated relative to signals from the adjusted peptide mix. Accordingly, these relative limits of quantitation (**Additional file 6, sheet 1**) indicate the signal ratios of the light peptides to the adjusted peptide mix that are still within the dynamic linear range according to the calibration curves.

<u>Acquisition and data analysis of the samples from glucan-producing yeasts</u>

Mass spectrometry (MS) analysis was performed on a Q Exactive HF mass spectrometer (Thermo Fisher Scientific) equipped with a Digital PicoView source (New Objective) and coupled to an M-Class UPLC (Waters). Eluents were 0.1% (v/v) formic acid for eluent A and 0.1% (v/v) formic acid, 99.9% (v/v) acetonitrile for eluent B. Samples were loaded onto an Acquity UPLC M-class Symmetry

C18 trap column (100 Å, 5 µm, 180 µm x 20 mm; Waters) coupled to an Acquity UPLC M-class HSS T3 column (1.8 µm, 75 µm x 250 mm; Waters).

Peptides were separated at a flow rate of 300 nl min^{-1} using a linear 45-min gradient from 5 to 35% B. The mass spectrometer was operated in PRM mode, using centroid as spectrum data type and acquiring full-scan MS spectra (150−2,000 m/z) at a resolution of 120,000 with a target value of 3,000,000. Precursors listed in the inclusion list (**Additional file 6, sheet 2**) were isolated within an isolation window of 1.4 m/z and using retention time windows of 5 min, and were fragmented by higher-energy collision dissociation fragmentation at a normalized collision energy of 27. PRM scans were obtained using centroid as spectrum data type, a resolution of 60,000, a maximum injection time of 119 ms and automatic gain control target of 200,000 ions.

Samples (**Additional file 6, sheet 4**) contained tryptic peptides from the corresponding yeast replicate (ca. 500 ng peptides on column; derived from the same amount of digested proteins for all samples), iRT peptides (Biognosys) and the adjusted pooled SIL peptide mix (SIL peptides with concentrations close to the endogenous counterparts) in 3% (v/v) acetonitrile, 0.1% (v/v) formic acid. Data was acquired without technical replication and using a block-randomized injection sequence, i.e. samples were grouped according to replicate number in a random fashion. All measurements from the strains mentioned above were included in the data analysis and reporting in the results section.

MS data was analysed in Skyline v20.2.0343 (MacCoss Lab Software) using the signals from the SIL peptides for single point calibration of the signals from the endogenous (light) peptides. Settings in Skyline, including the spectral library, were as those for the calibration curve but setting heavy as internal standard type. Peak assignment and selection of quantitation windows were done automatically but manually inspected and slightly adapted if needed to remove interferences. Transitions y9+ from peptide ISA1_1, y6+ from peptide SS2_3, and y3+ from peptide BE2_1 with charge 3+ were excluded from quantitation because their elution profile markedly deviated from those of the other transitions assigned to the same precursor and/or because they appeared in samples known not to contain the associated light peptides (e.g. in wild-type samples).

Normalized peptide abundances were checked for being higher than their lower limits of quantitation (**Additional file 6, sheet 8**). If only a single peptide abundance was below the lower limit of quantitation, protein abundances were calculated based on the remaining two peptides; this was the case for SS1 quantification in the three replicates of strain SS1-F and for SS3 quantification in one replicate of strain SS3-F. If all peptide abundances were below the lower limit of quantitation, the protein abundance was still determined based on all these peptides, but their limitation clearly marked in the associated Figures and in **Additional file 6, sheet 7**; this situation was only observed in strains in which the corresponding proteins were not expected to be present (e.g., for all heterologously expressed proteins in WT). Protein abundances were calculated in Skyline-daily 20.2.1.404 employing Tukey's median polish as summary method.

References:

Durek, P., Schmidt, R., Heazlewood, J.L., Jones, A., MacLean, D., Nagel, A., Kersten, B., and Schulze, W.X. (2009). PhosPhAt: The Arabidopsis thaliana phosphorylation site database. An update. Nucleic Acids Res. **38**: 828–834.

MacLean, B., Tomazela, D.M., Shulman, N., Chambers, M., Finney, G.L., Frewen, B., Kern, R., Tabb, D.L., Liebler, D.C., and MacCoss, M.J. (2010). Skyline: An open source document editor for creating and analyzing targeted proteomics experiments. Bioinformatics **26**: 966–968.

Roitinger, E., Hofer, M., Köcher, T., Pichler, P., Novatchkova, M., Yang, J., Schlögelhofer, P., and Mechtler, K. (2015). Quantitative phosphoproteomics of the ataxia telangiectasia-mutated (ATM) and ataxia telangiectasia-mutated and Rad3-related (ATR) dependent DNA damage response in arabidopsis thaliana. Mol. Cell. Proteomics **14**: 556–571.

Schnatbaum, K., Zerweck, J., Nehmer, J., Wenschuh, H., Schutkowski, M., and Reimer, U. (2011). SpikeTides™—proteotypic peptides for large-scale MS-based proteomics. Nat. Methods **8**: i–ii.

Soste, M., Hrabakova, R., Wanka, S., Melnik, A., Boersema, P., Maiolica, A., Wernas, T., Tognetti, M., Von Mering, C., and Picotti, P. (2014). A sentinel protein assay for simultaneously quantifying cellular processes. Nat. Methods **11**: 1045–1048.

Uhrig, R.G., Schläpfer, P., Roschitzki, B., Hirsch-Hoffmann, M., and Gruissem, W. (2019). Diurnal changes in concerted plant protein phosphorylation and acetylation in Arabidopsis organs and seedlings. Plant J. **99**: 176–194.
