# Supplementary material for: Tuning heterologous glucan biosynthesis in yeast to understand and exploit plant starch diversity
Source: BMC Biol. 2022 Sep 24;20:207. doi: 10.1186/s12915-022-01408-x (PMC9509603; doi:10.1186/s12915-022-01408-x)

Additional File 12. Calibration curves of heavy-labelled standard peptides tested for quantification.

Curves and accompanying statistices were established in Skyline software using a 6-point dilution series starting from undiluted pooled peptide mix (10^0) and five 10-fold dilutions. measured in two (10^0 to 10^{-3} dilutions) or three technical (10^{-4} and 10^{-5} dilutions and blanks) replicates. The third blank was not considered as it contained carryover. The lowest dilution (10^{-5}) and, sometimes, other dilutions (indicated by open rectangles), were excluded from the analyses if they impeded the calculation of figures of merit in Skyline. The purple and green line show the limit of detection (LOD) and lower limit of quantitation (LOQ), respectively. LODs represent the peak areas of blanks plus two standard deviations, and LOQs were calculated by setting their maximum bias and CV to 35% each. In cases where the apparent LOD is higher than the LOQ, the LOD could not be calculated properly since at least one blank had no peak at all within the detection window, resulting in a peak area of 0 that could not be log transformed (e.g. for peptide SS1_3). The red line is the calculated concentration of a randomly chosen run.

Additional File 12 of Pfister et al.

1) SS1_1: AVEEGAAEVGIPSGK

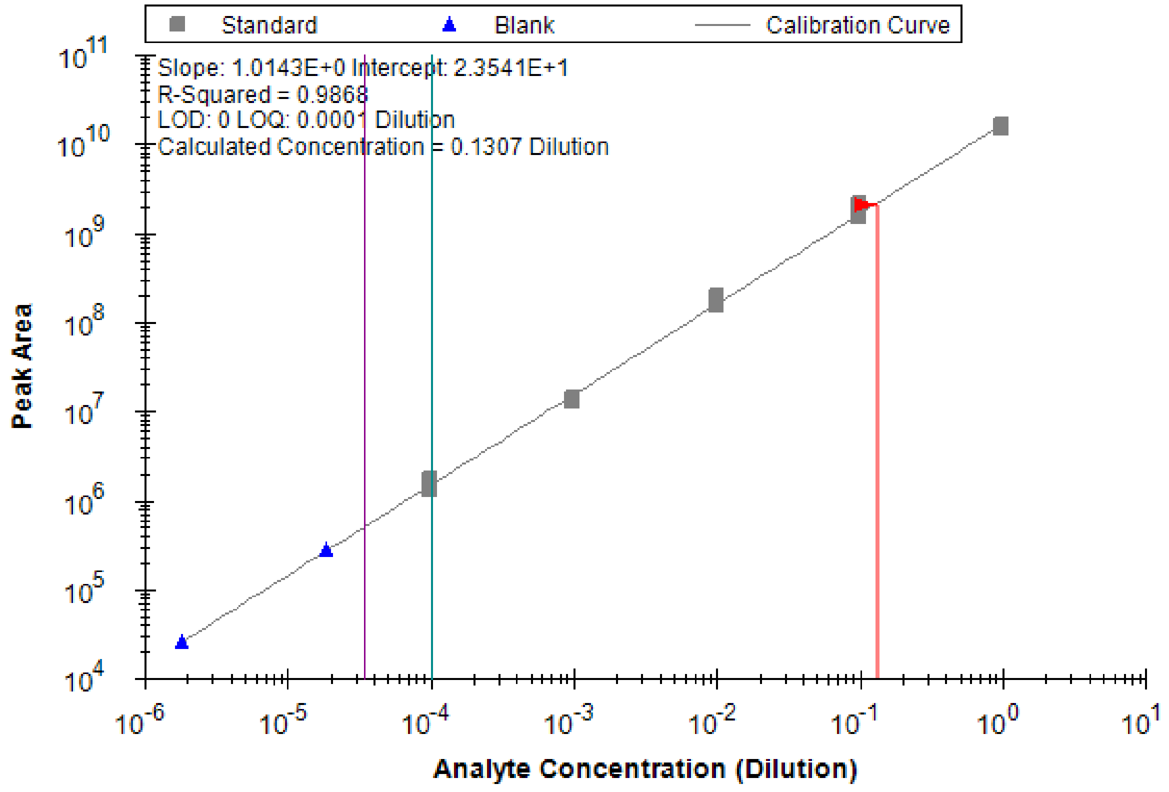

2) SS1_2: TGGLGDVCGSLPIALAGR

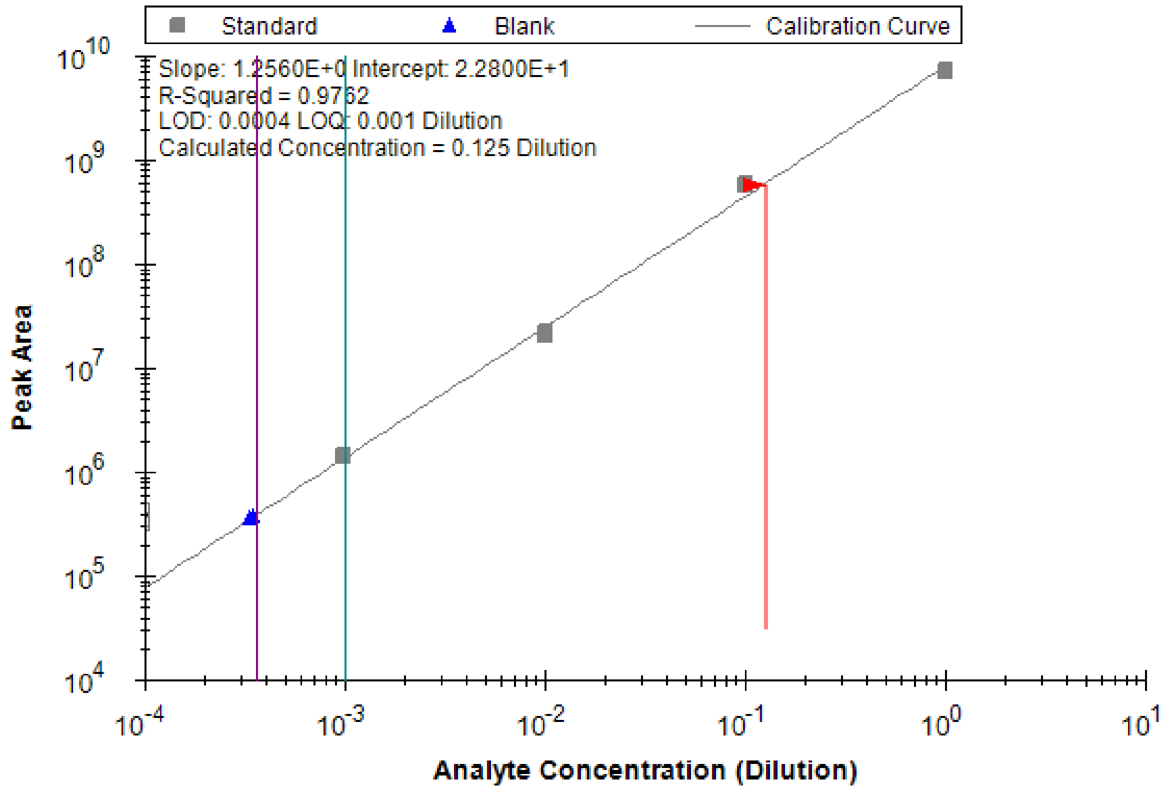

3) SS1_3: THALDTGEAVNVLK

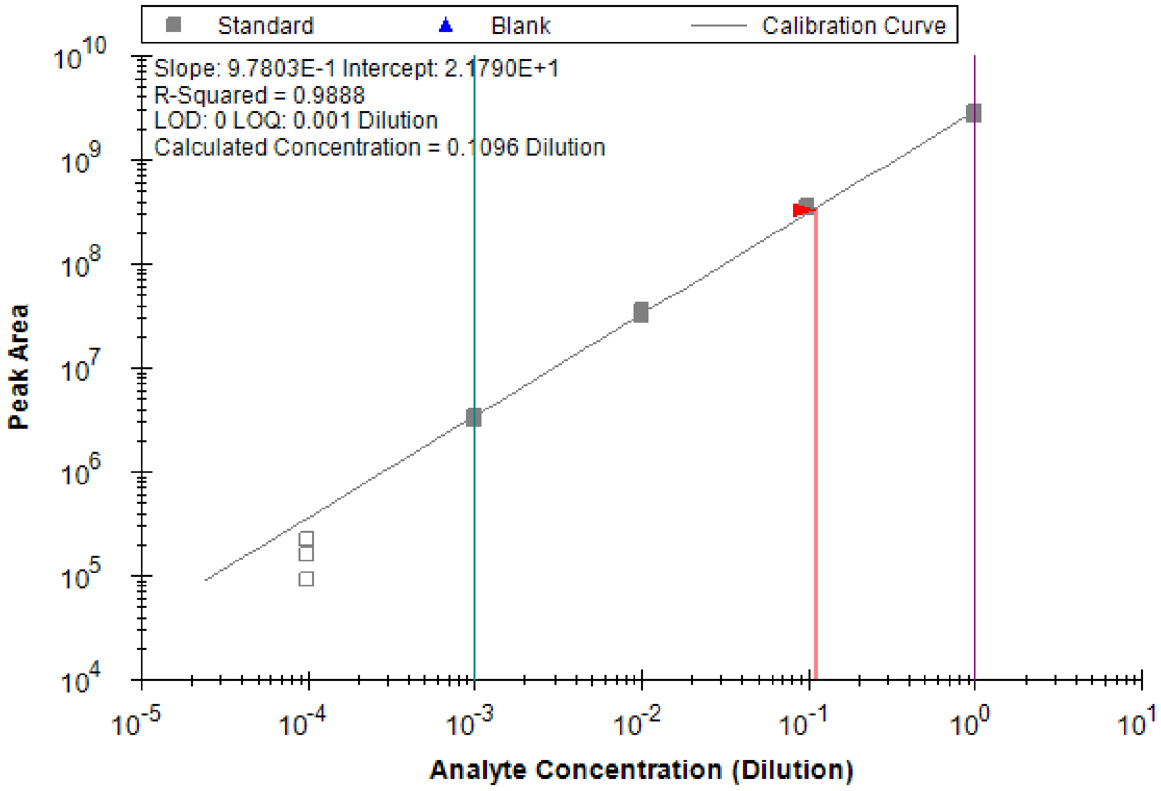

4) SS2_1: AGAFWSDPLPSYLTK

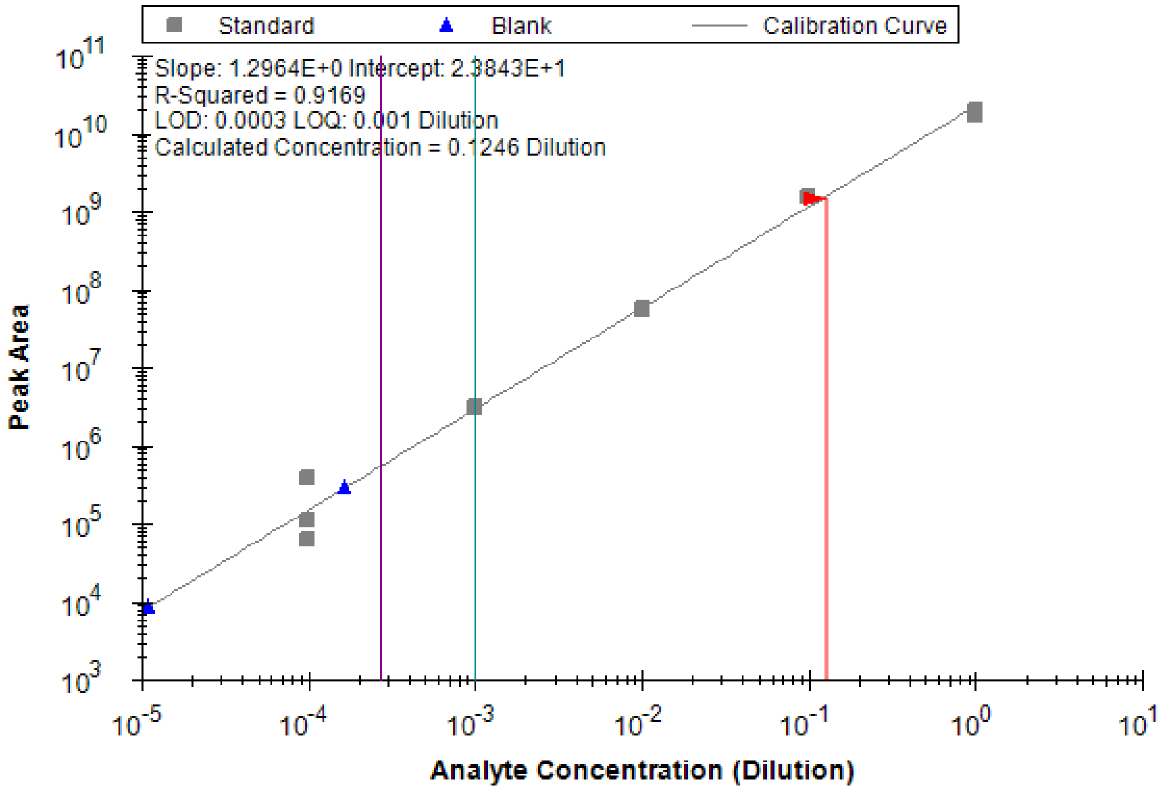

5) SS2_2: TGGLGDVAGALPK

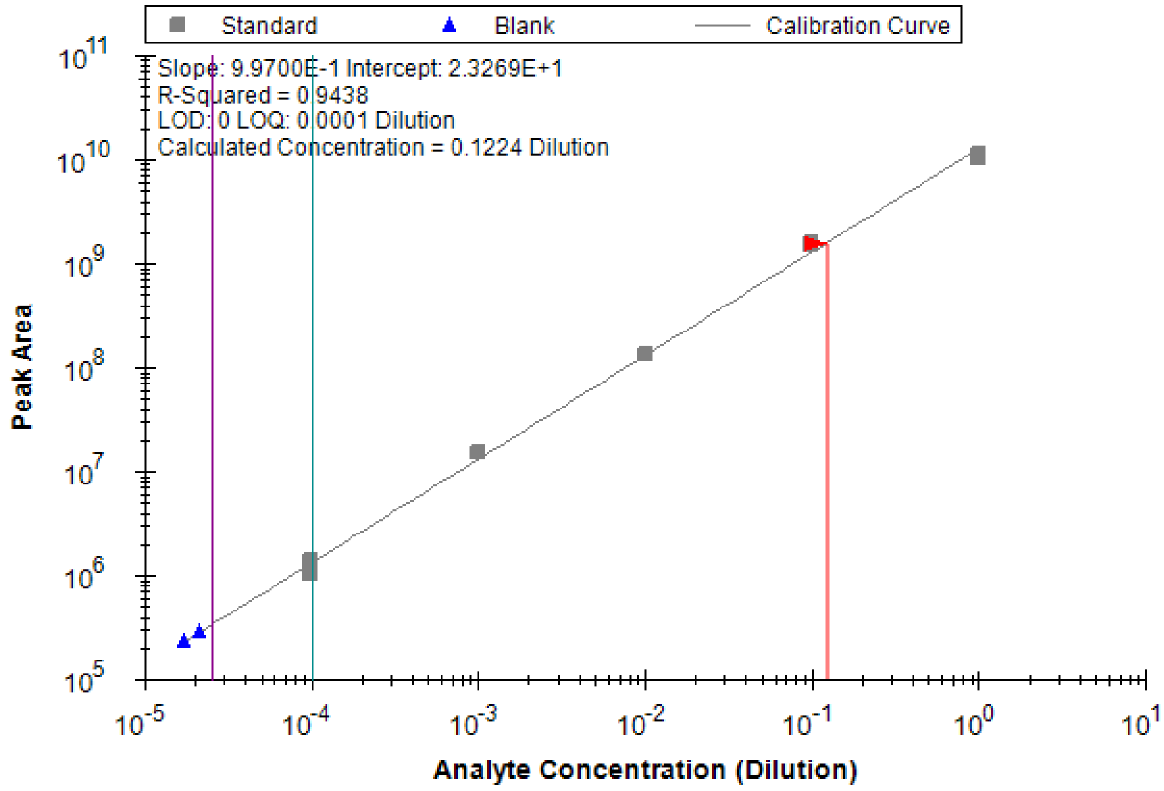

6) SS2_3: LYDPVGGEHFNIFAAGLK

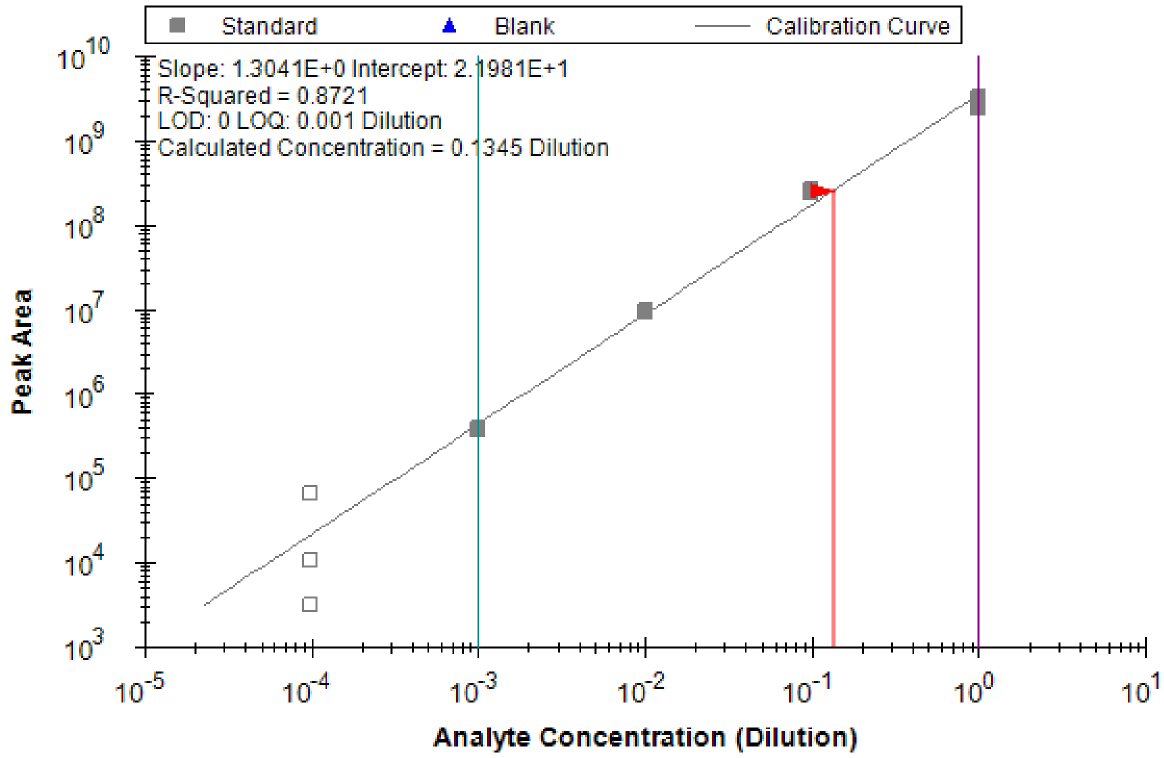

7) SS3_1: VDFENFLLEEK

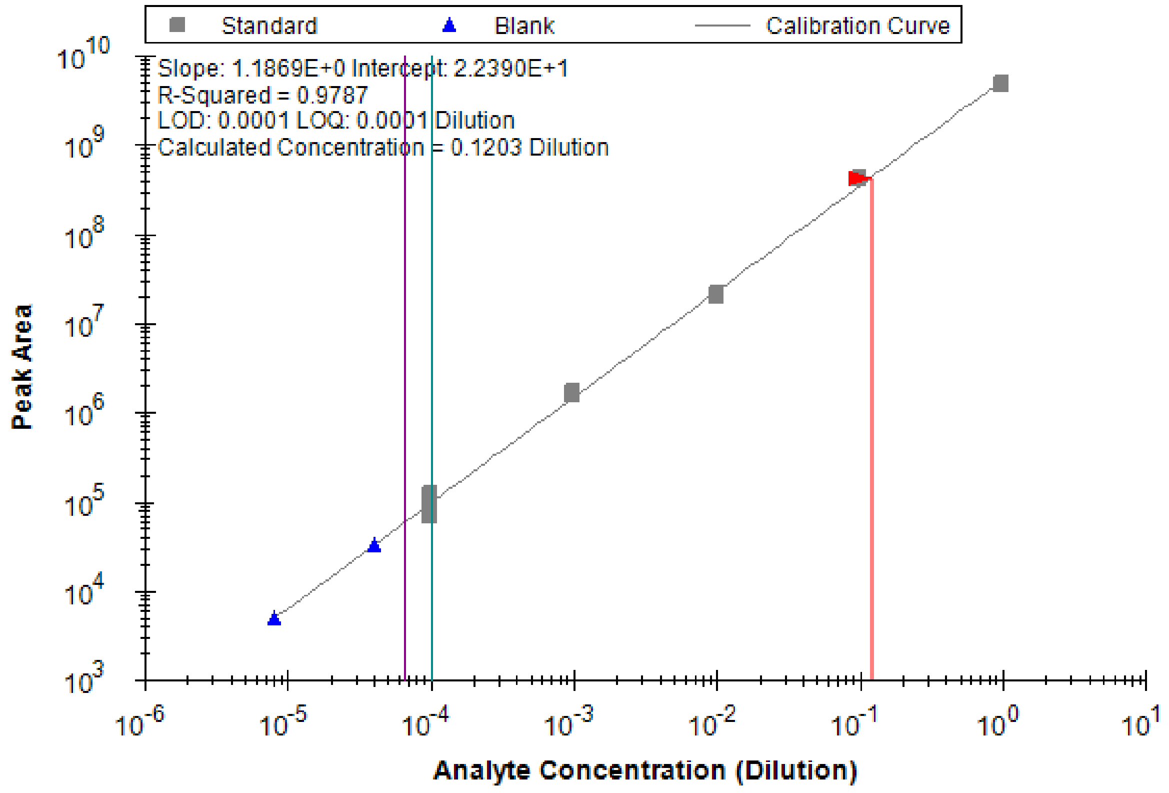

8) SS3_2: VGGLGDVVTSLSR

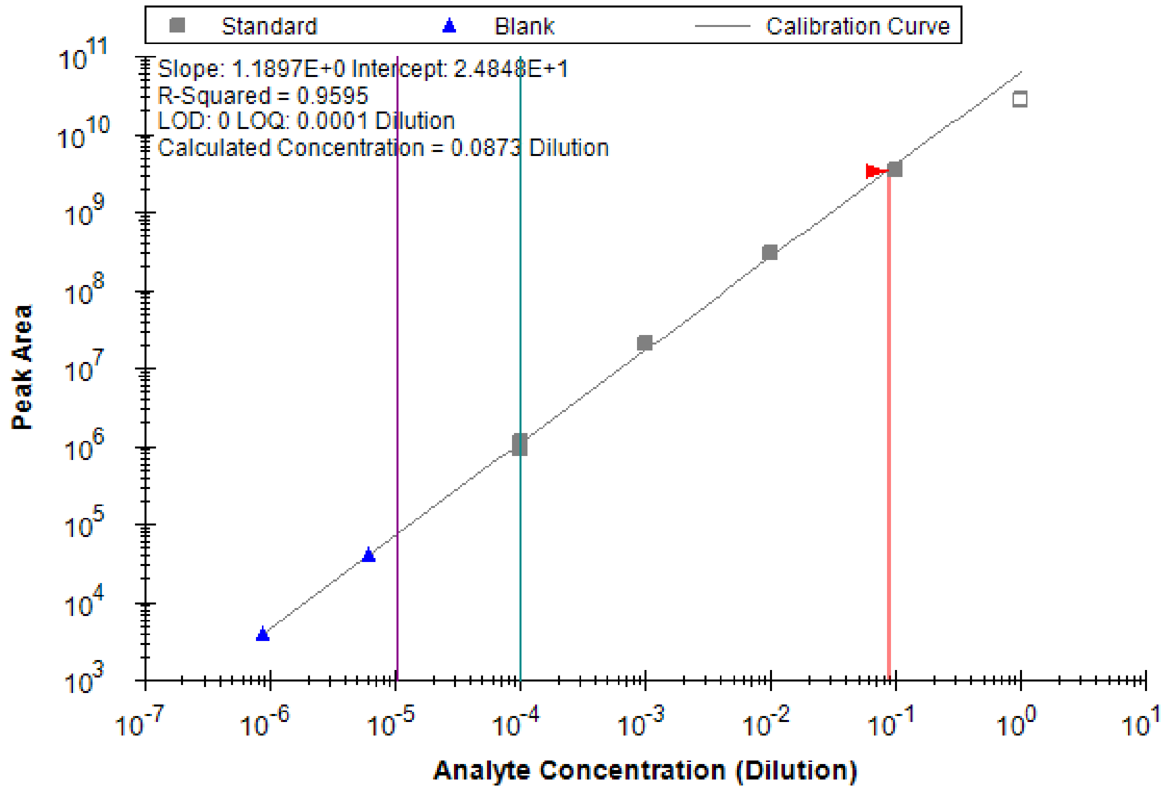

9) SS3_3: SADFPVVGIITR

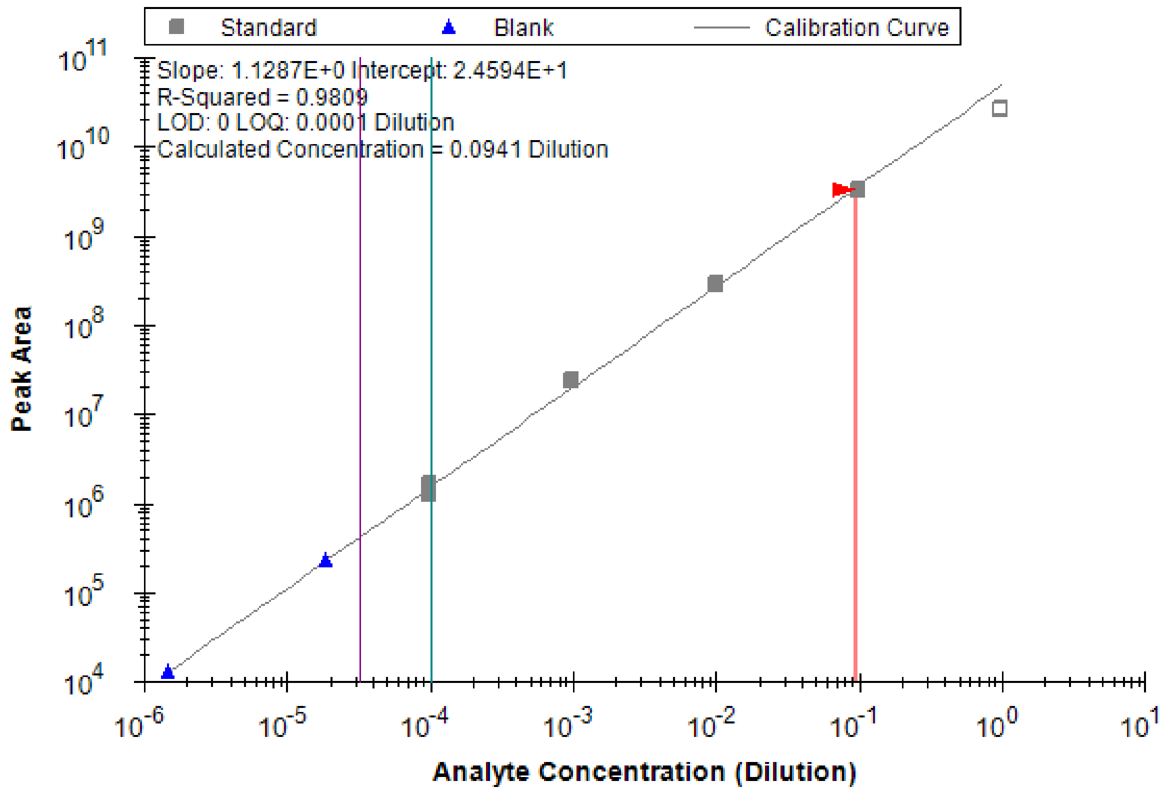

10) SS4_1: AHVELLEEQLEK

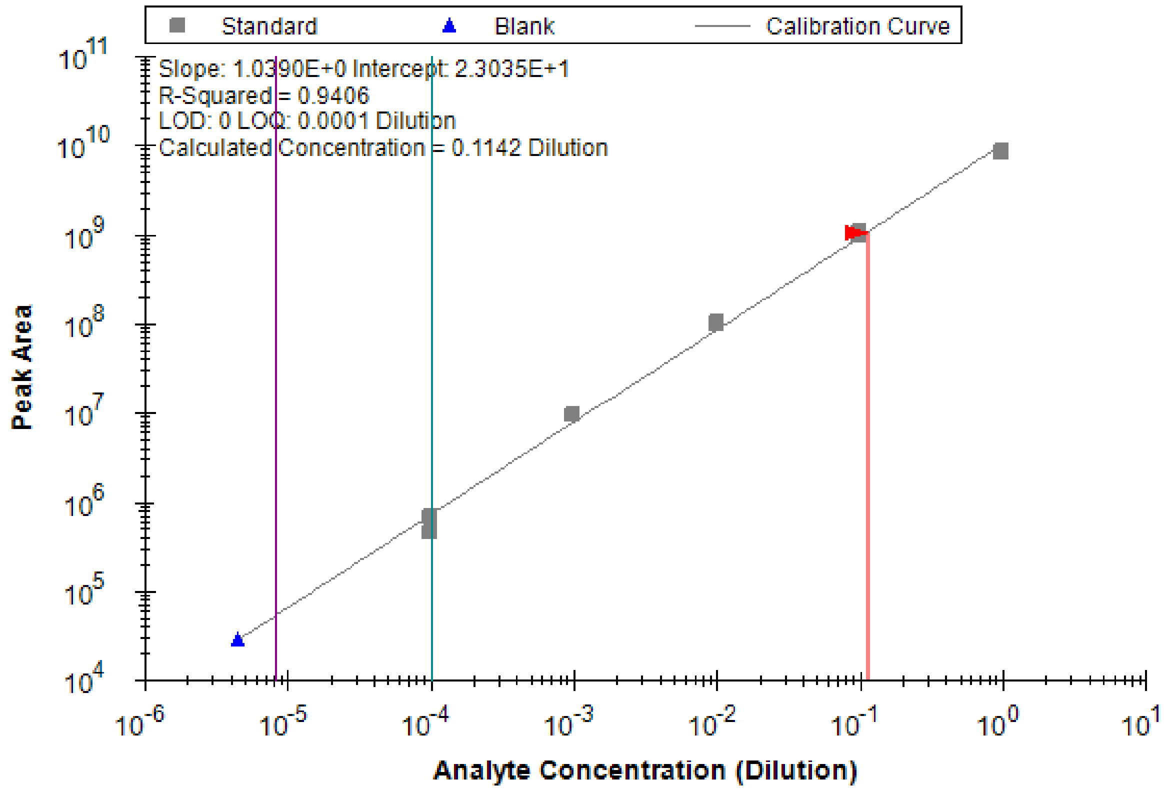

11) SS4_2: LSVSQEDVSQLSTLK

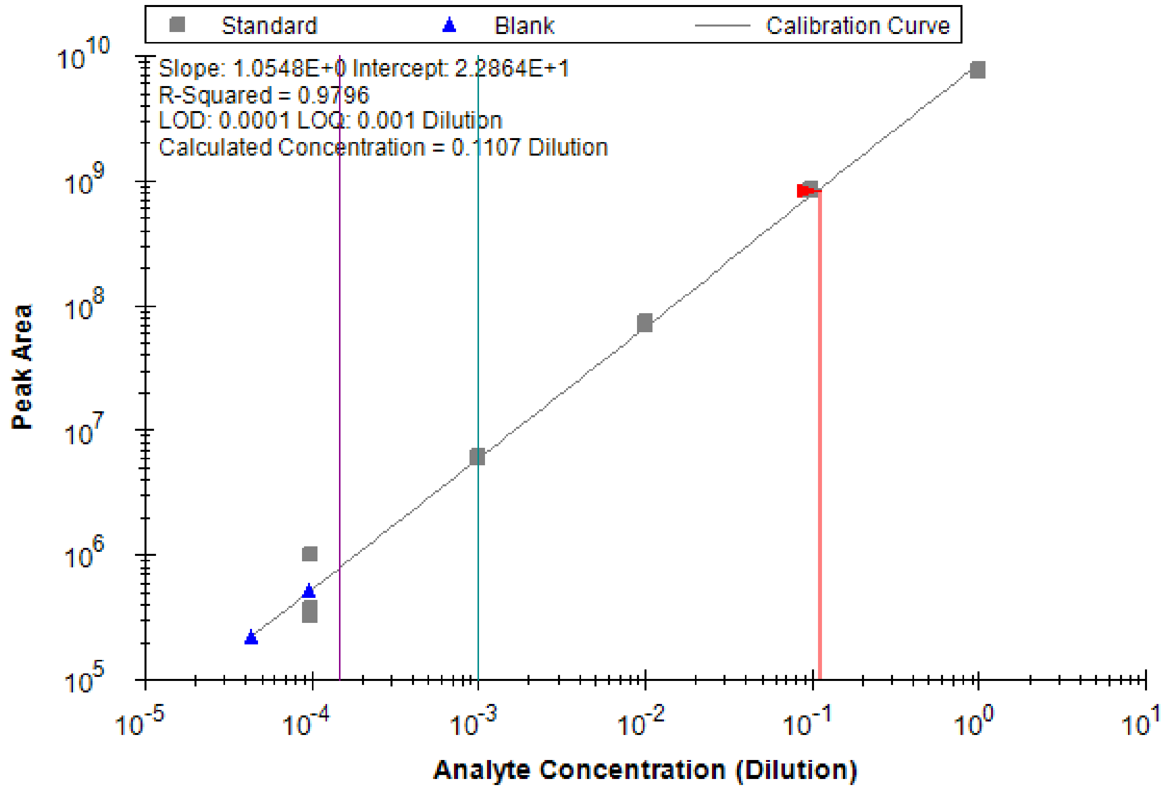

12) SS4_3: VETLQLLLDR

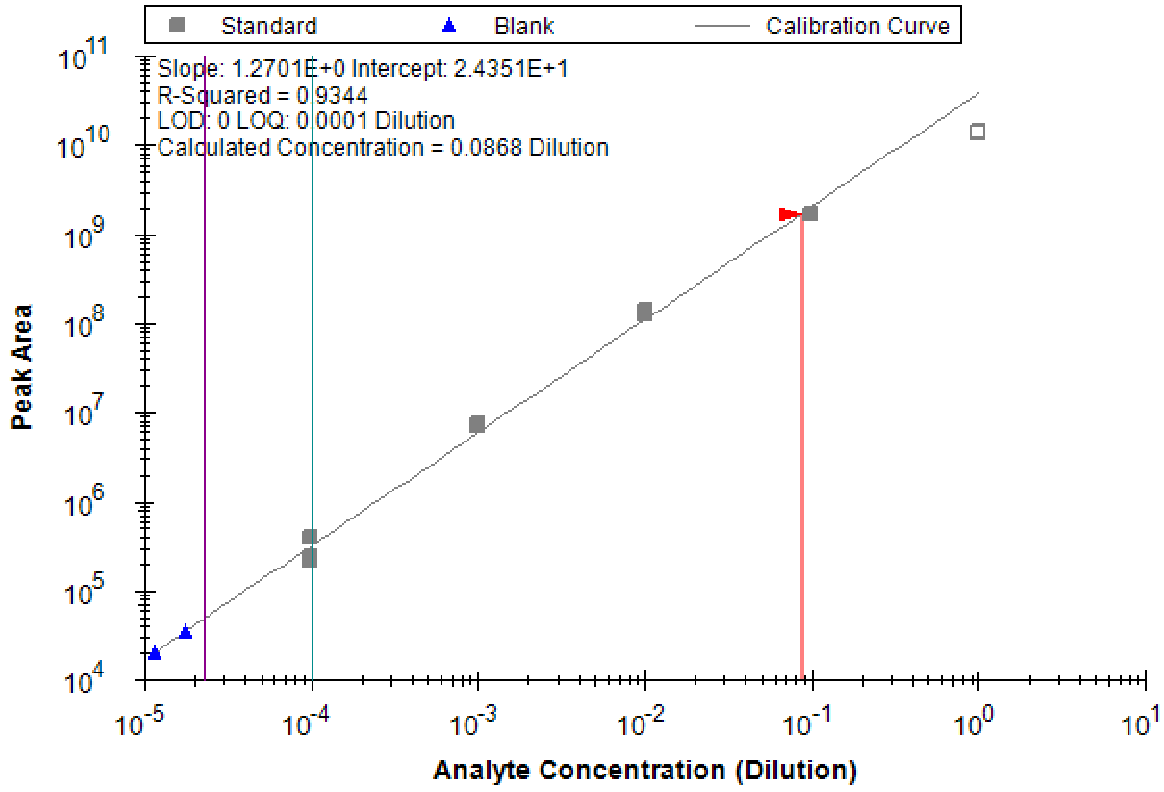

Additional File 12 of Pfister et al.

13) BE2_1: TYNNHLDYR

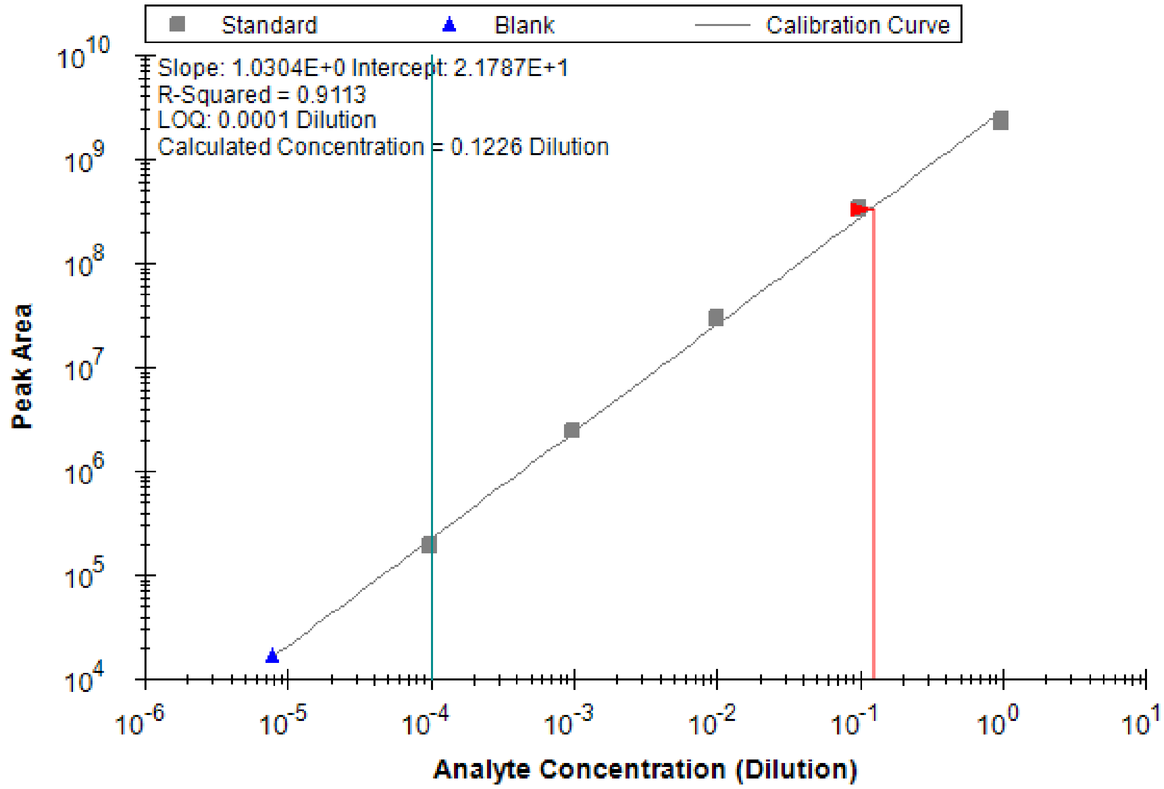

14) BE2_2: IVLDSDDPLFGGFNR

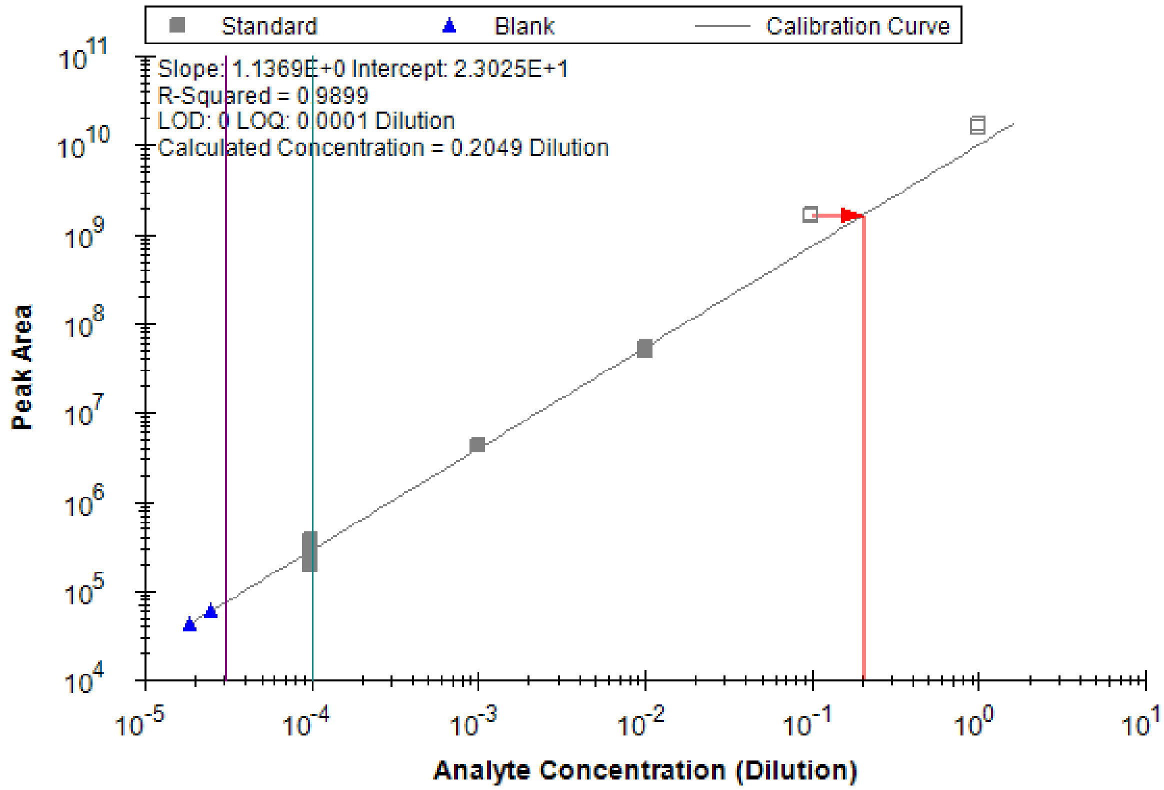

15) BE2_3: YEGGLEAFSR

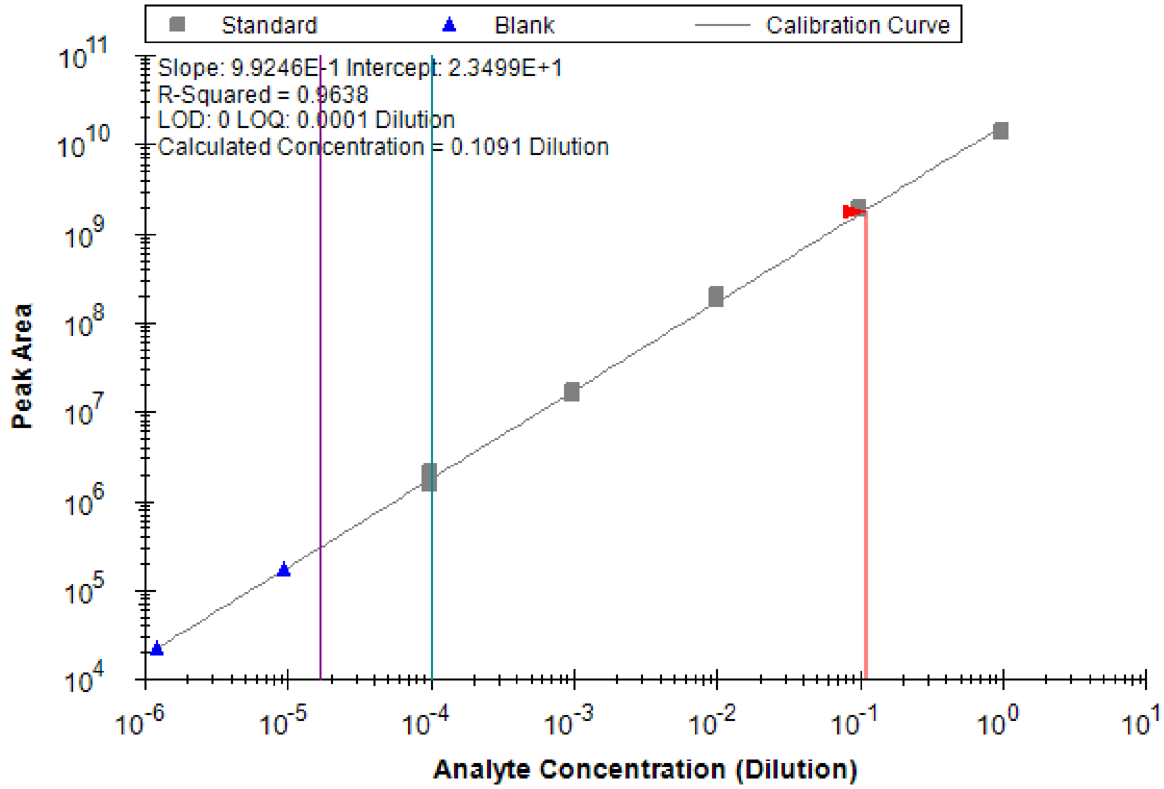

16) BE3_1: GYEIFGFTR

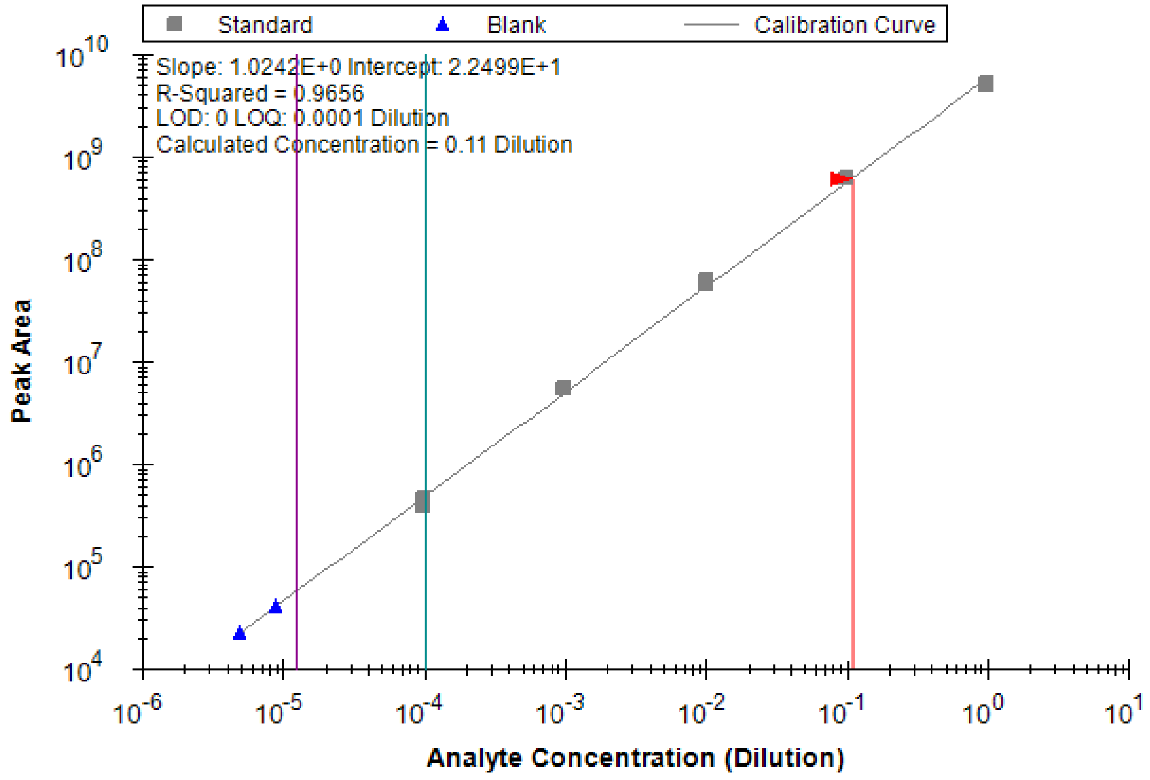

17) BE3_2: TDQHLPDGR

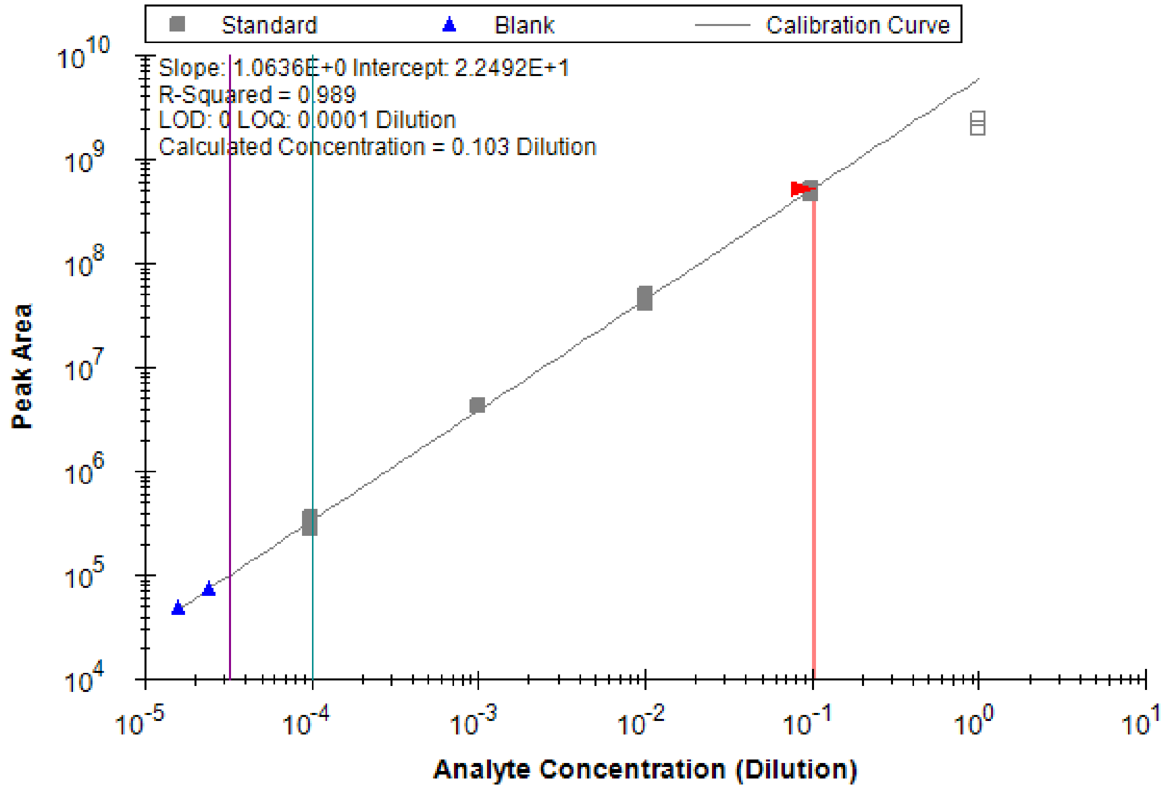

18) BE3_3: VIAGNNGSYDK

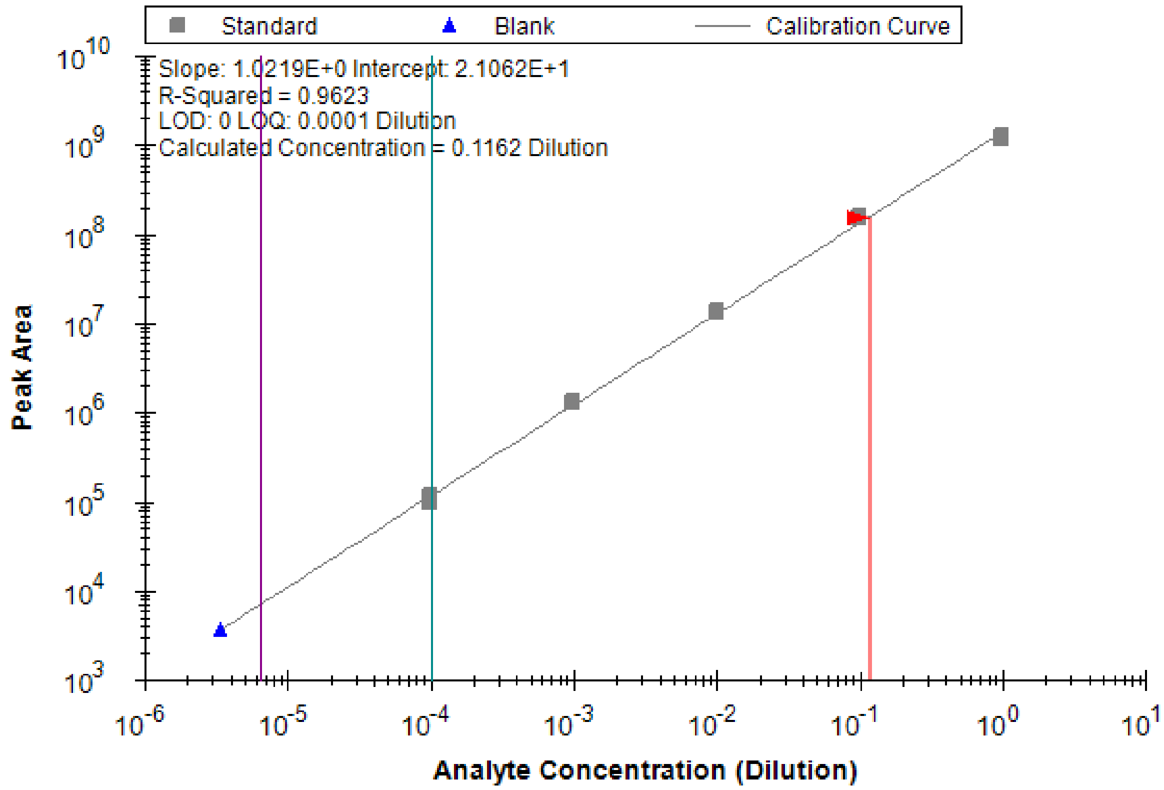

19) BE3_4: IVLDSDNSLFGGFNR

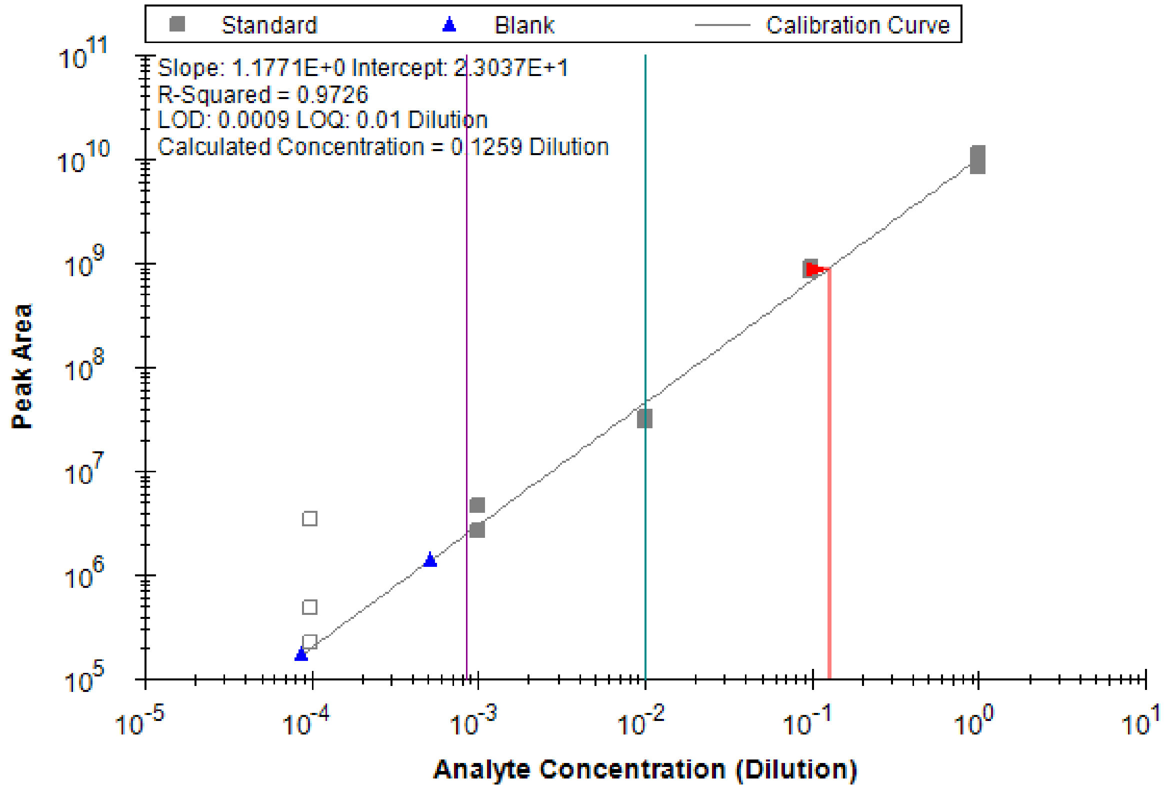

20) ISA1_1: VTEEIQLDPSR

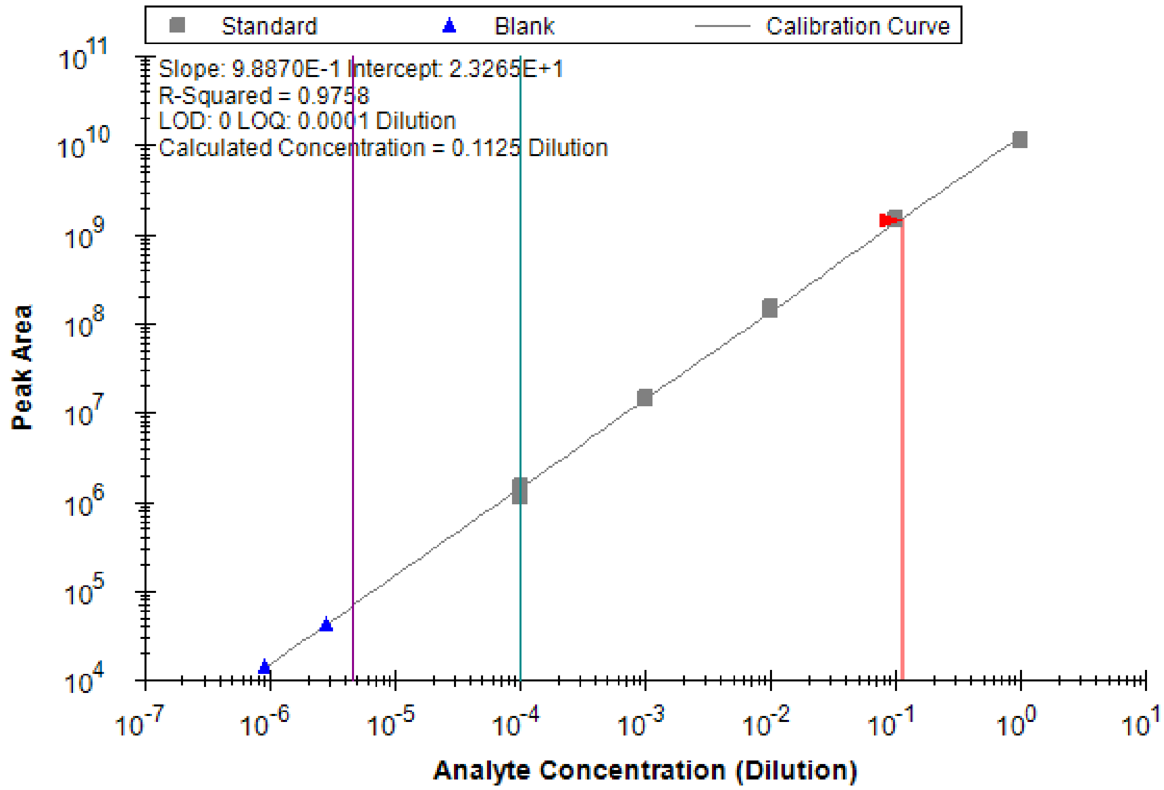

21) ISA1_2: IEFPGTYQGVAEK

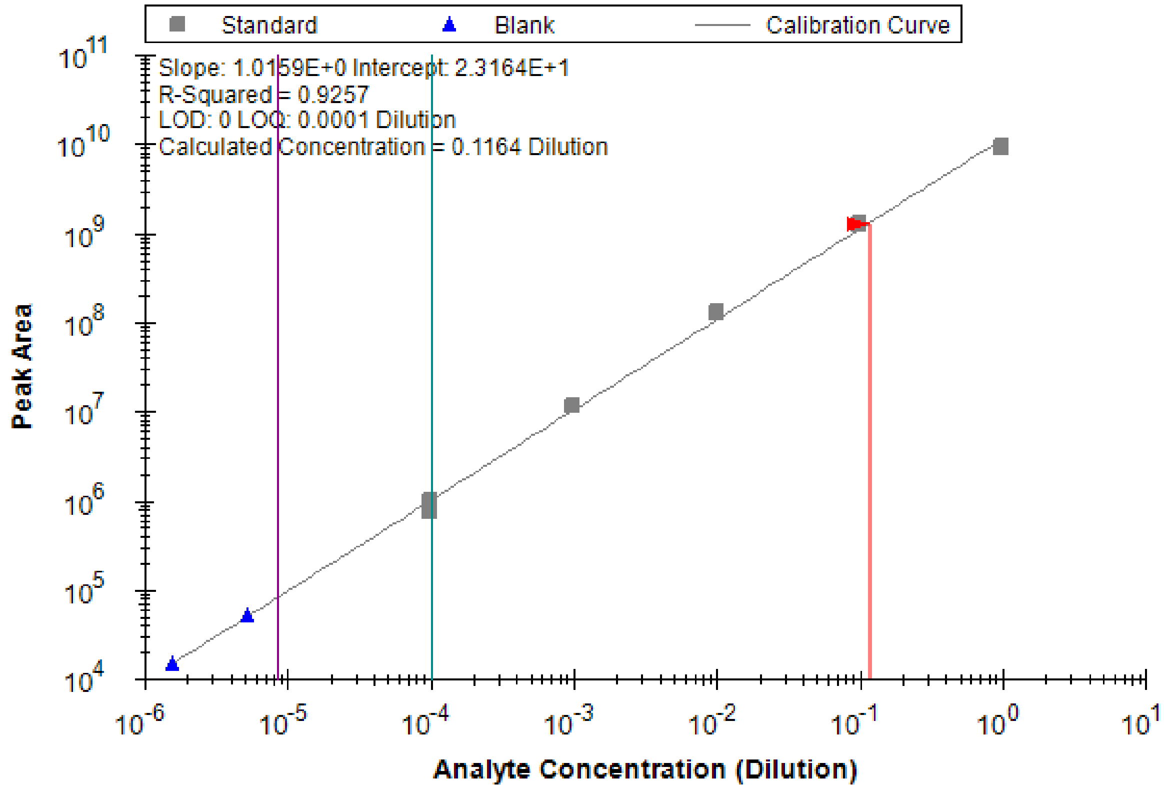

22) ISA1_3: YASASSNNFAGR

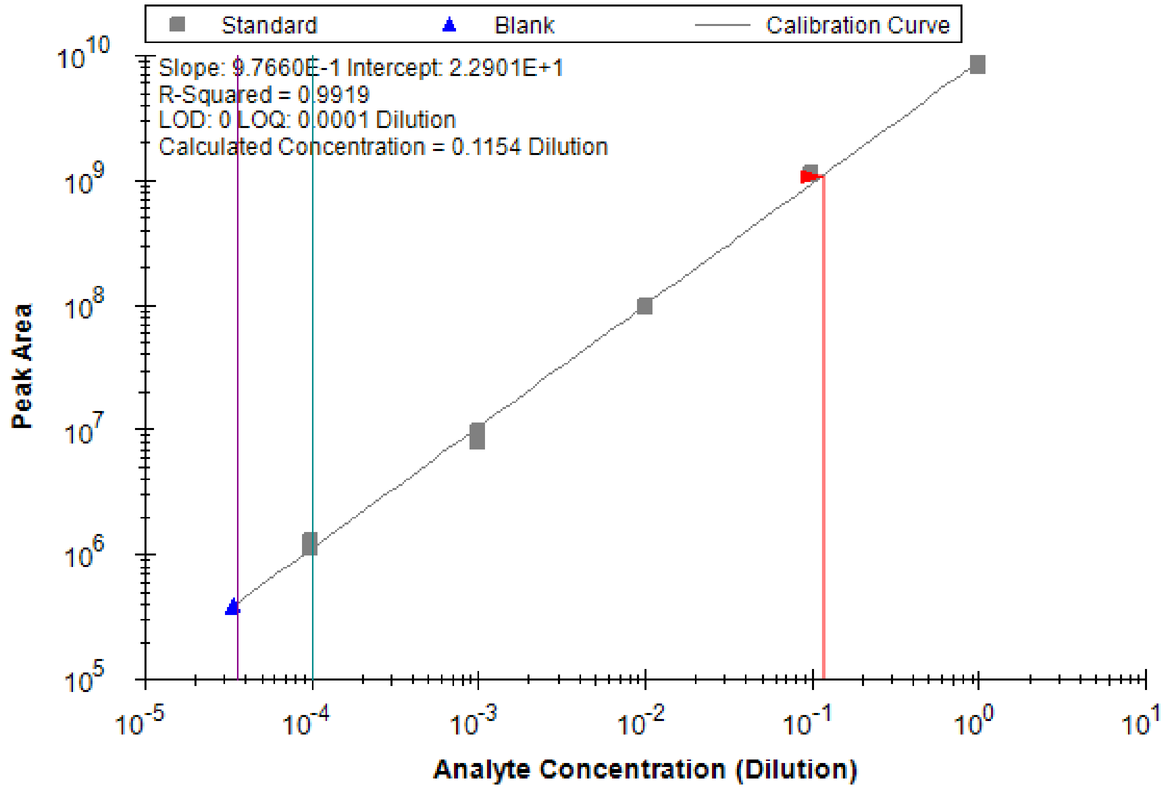

23) ISA2_1: SPSFDWGEDVSPNIPLEK

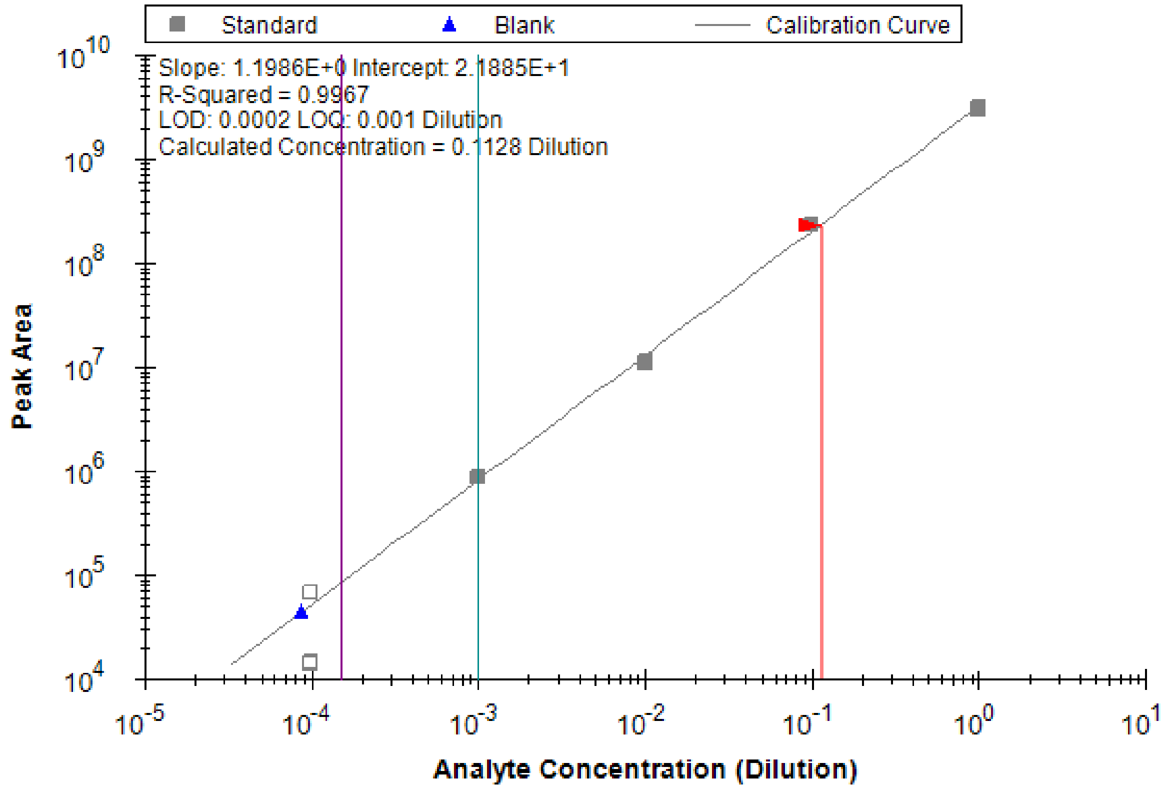

24) ISA2_2: GSPLLESR

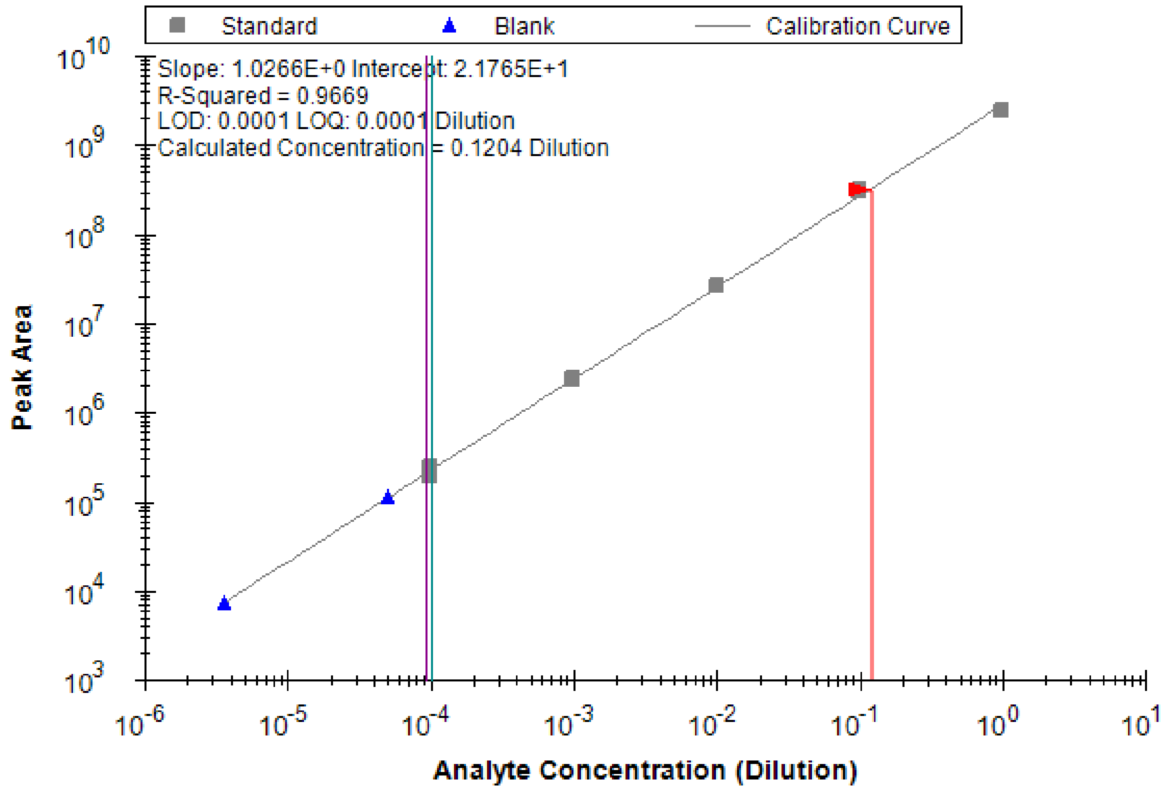

25) ISA2_3: DFLKPENIVWYANDQTTPK

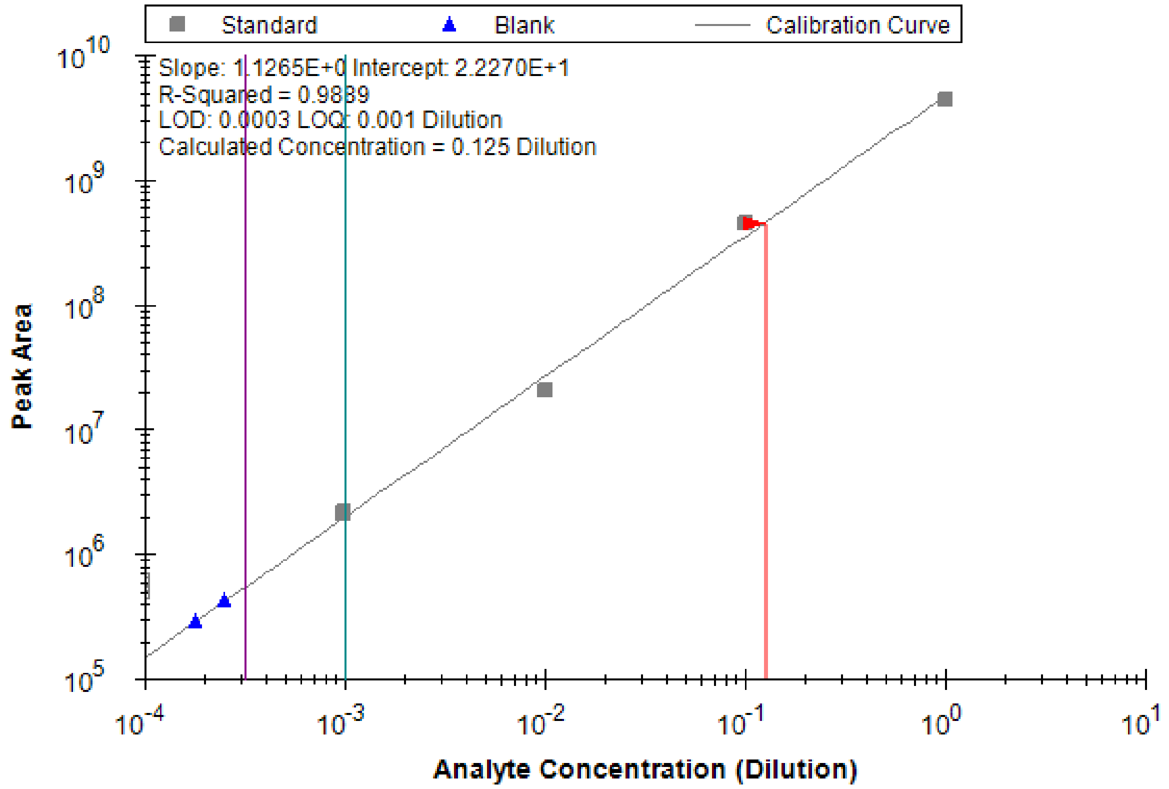

26) glgC_1: DVGTLEAYWK

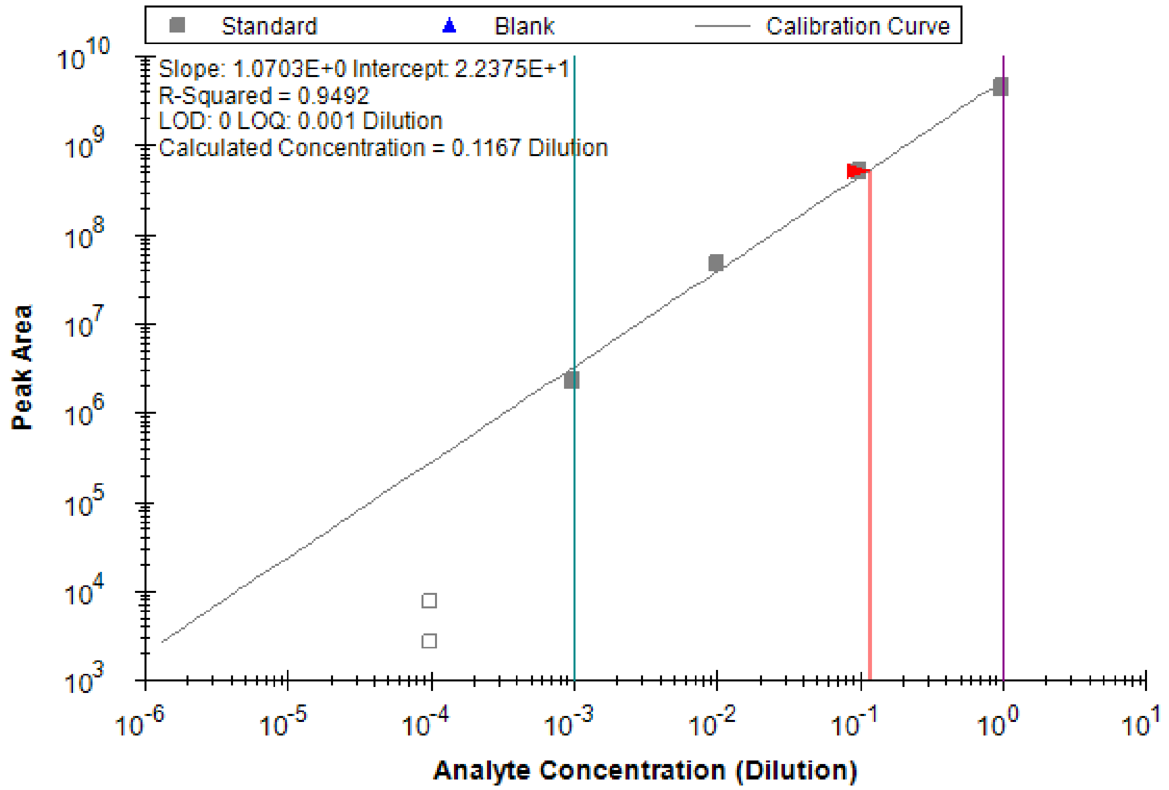

27) glgC_2: SEEGIVLVTR

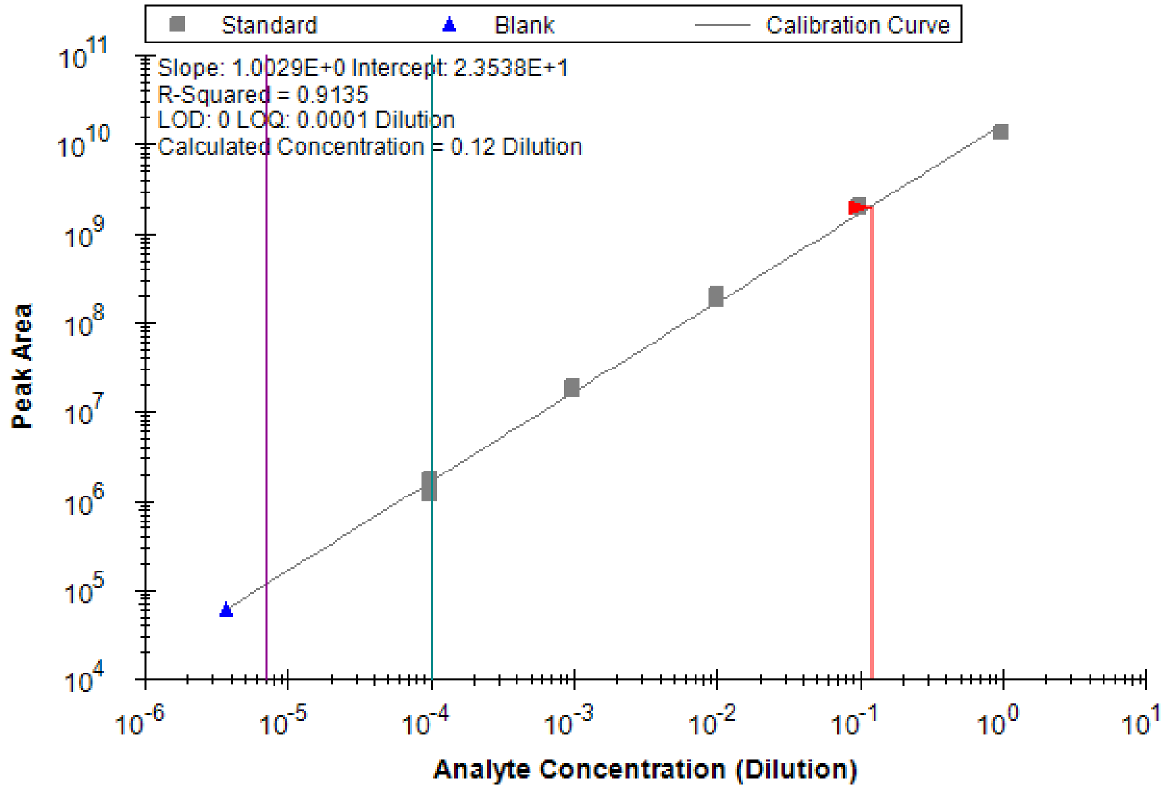

28) glgC_3: TYNESLPPAK

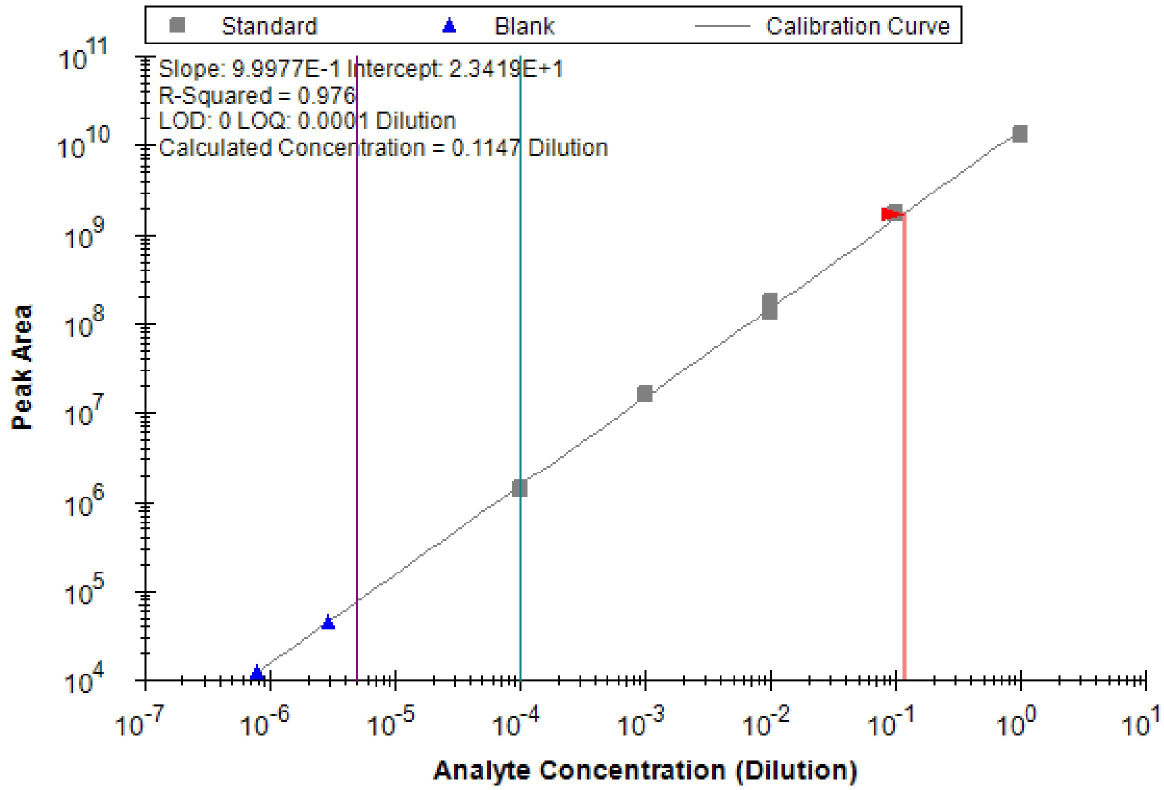

Additional File 12 of Pfister et al.

29) mCherry_1: HPADIPDYLK

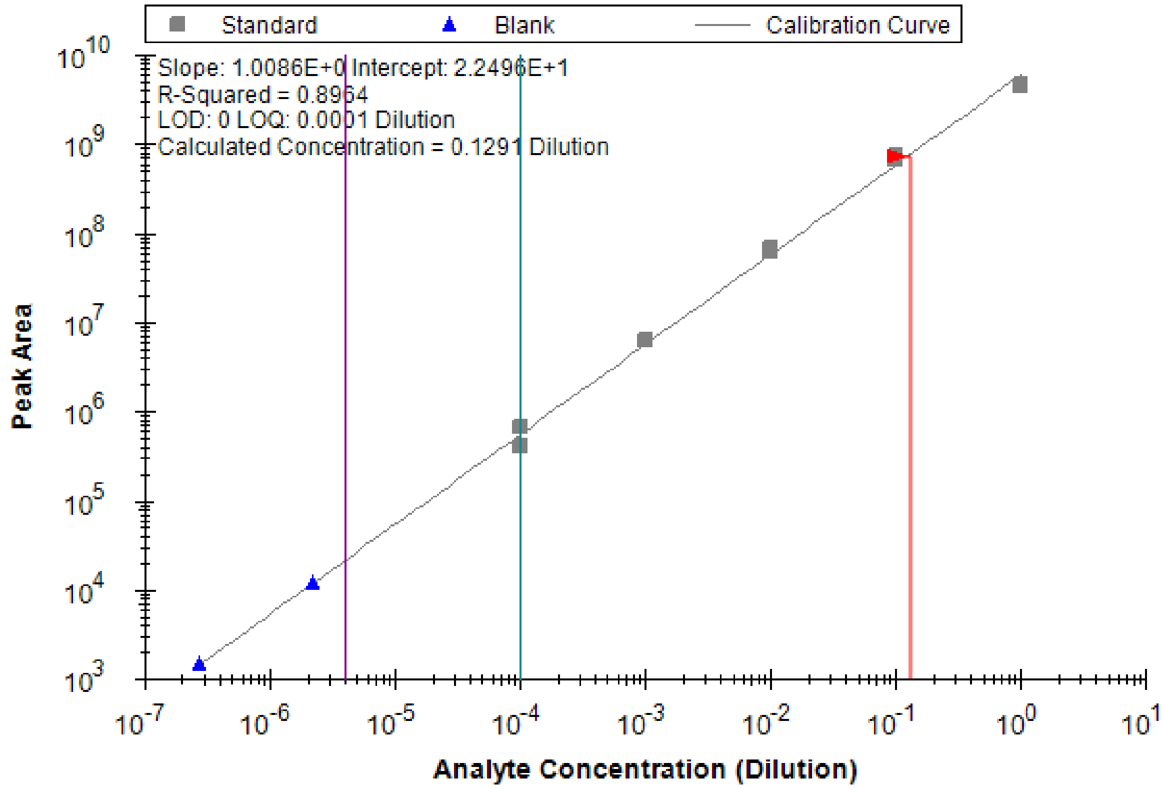

30) mCherry_2: LSFPEGFK

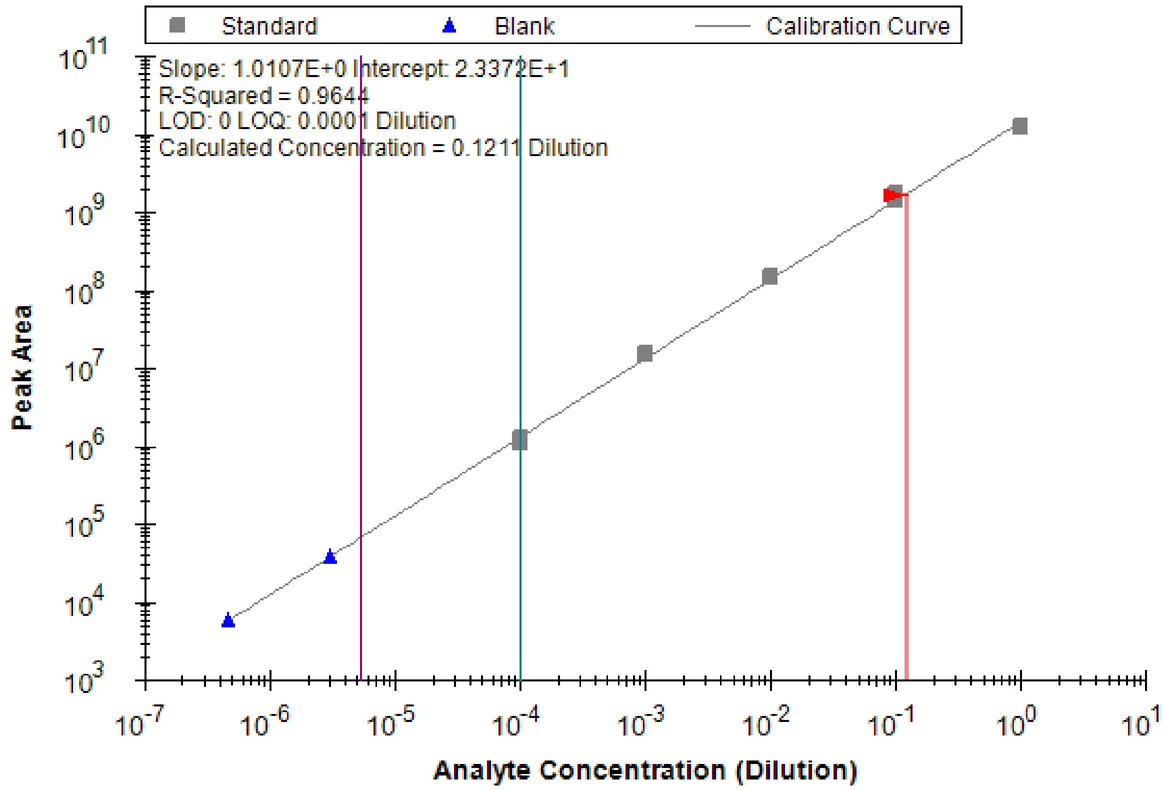

Additional File 12 of Pfister et al.

31) mCherry_3: LDITSHNEDYTIVEQYER

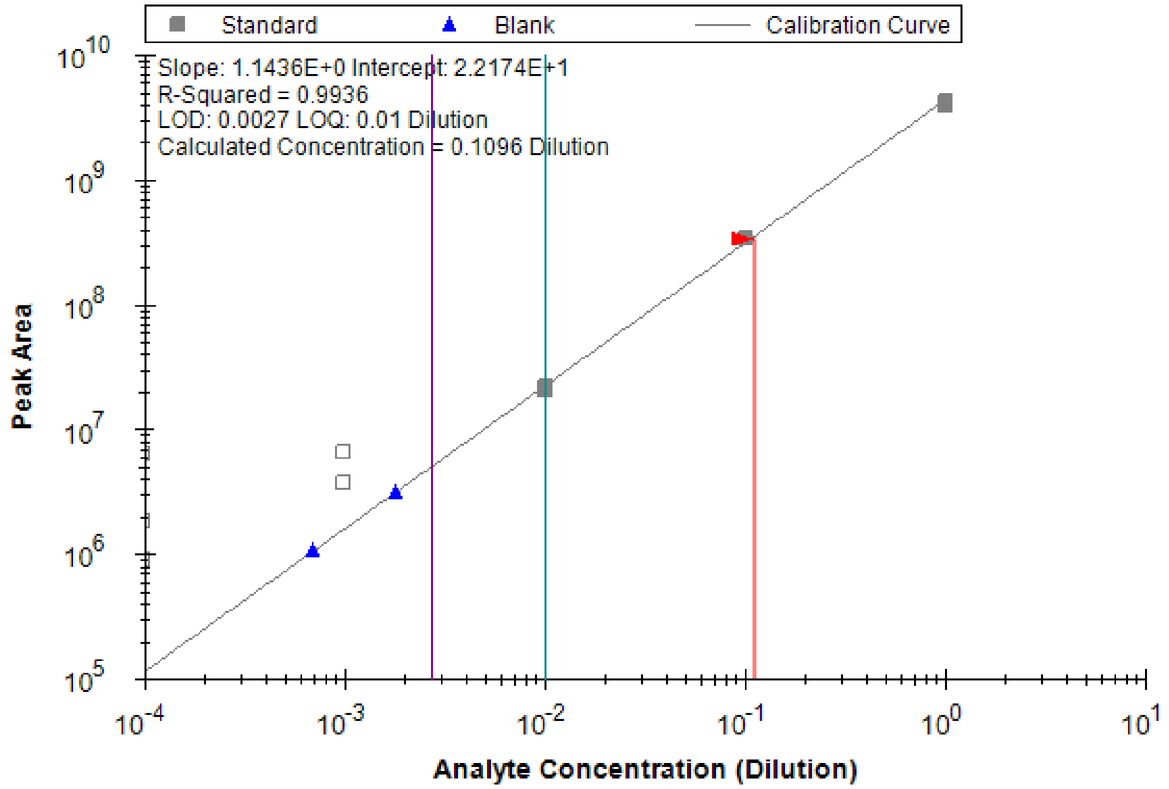

32) N1P9R2_1: GVSDEANLNETGR

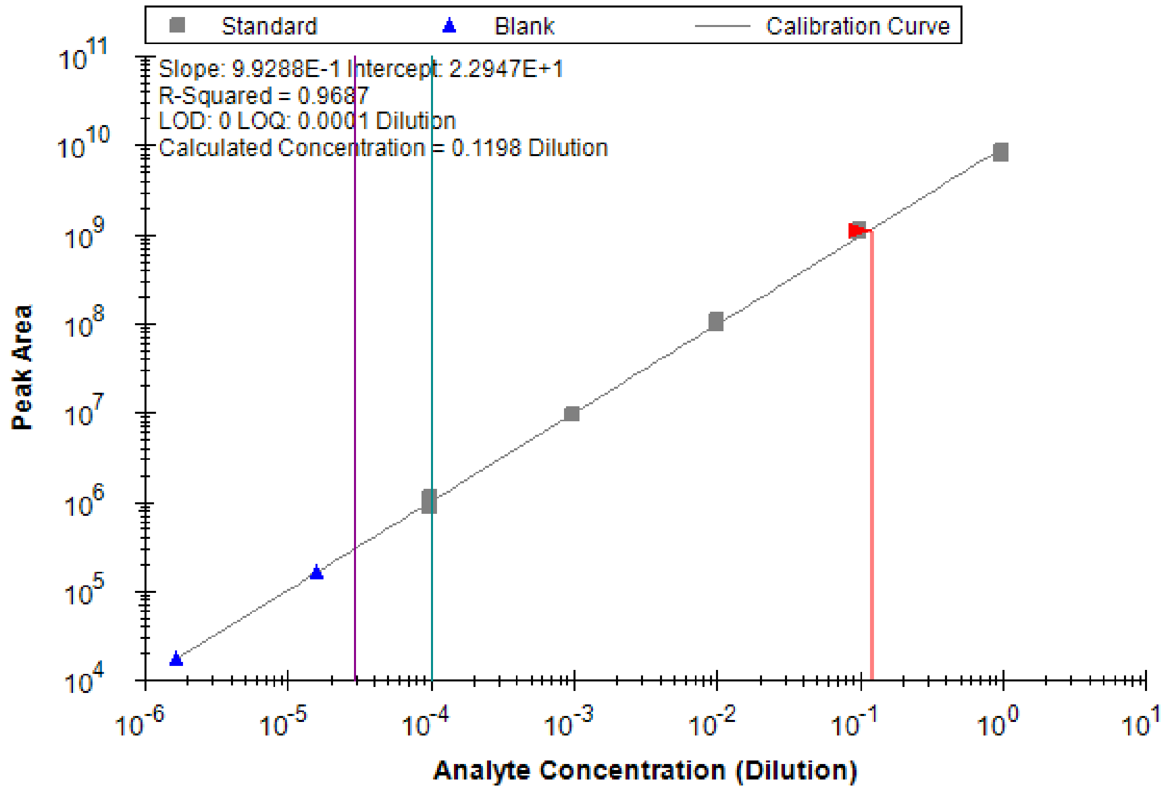

33) N1P9R2_2: VVDALGNPIDGK

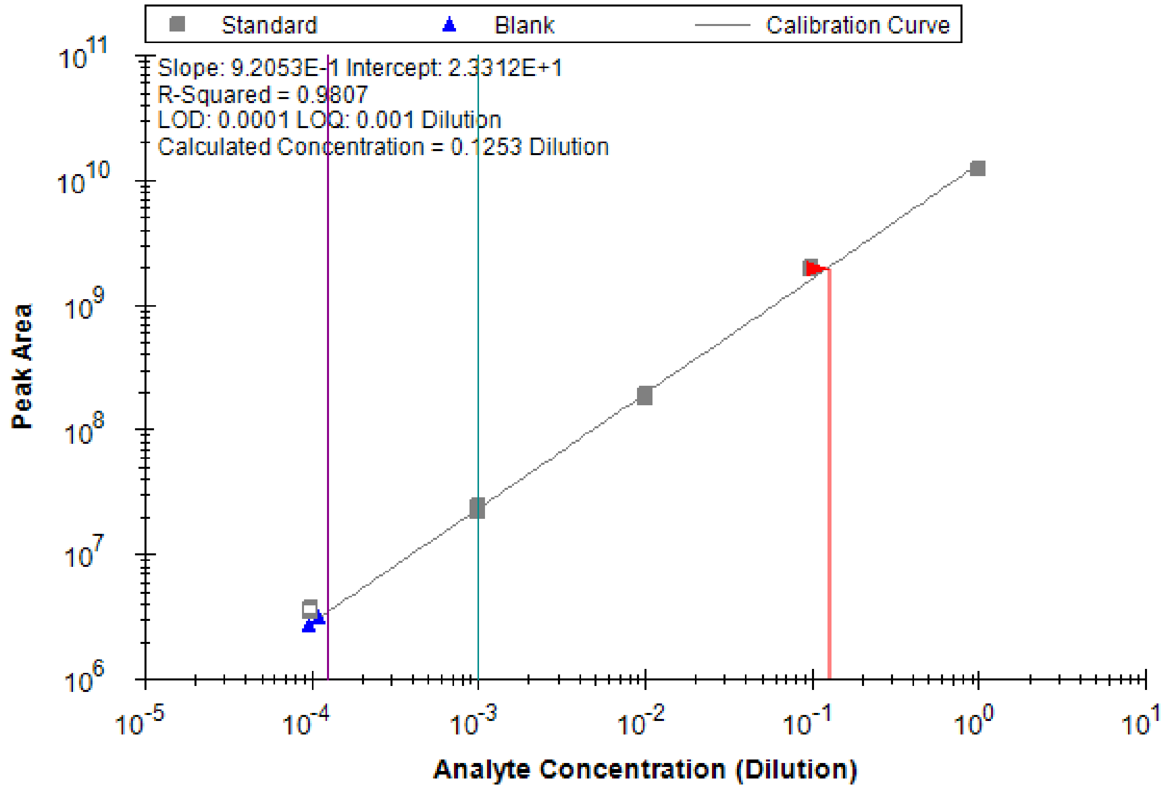

34) N1P9R2_3: AVDALVPIGR

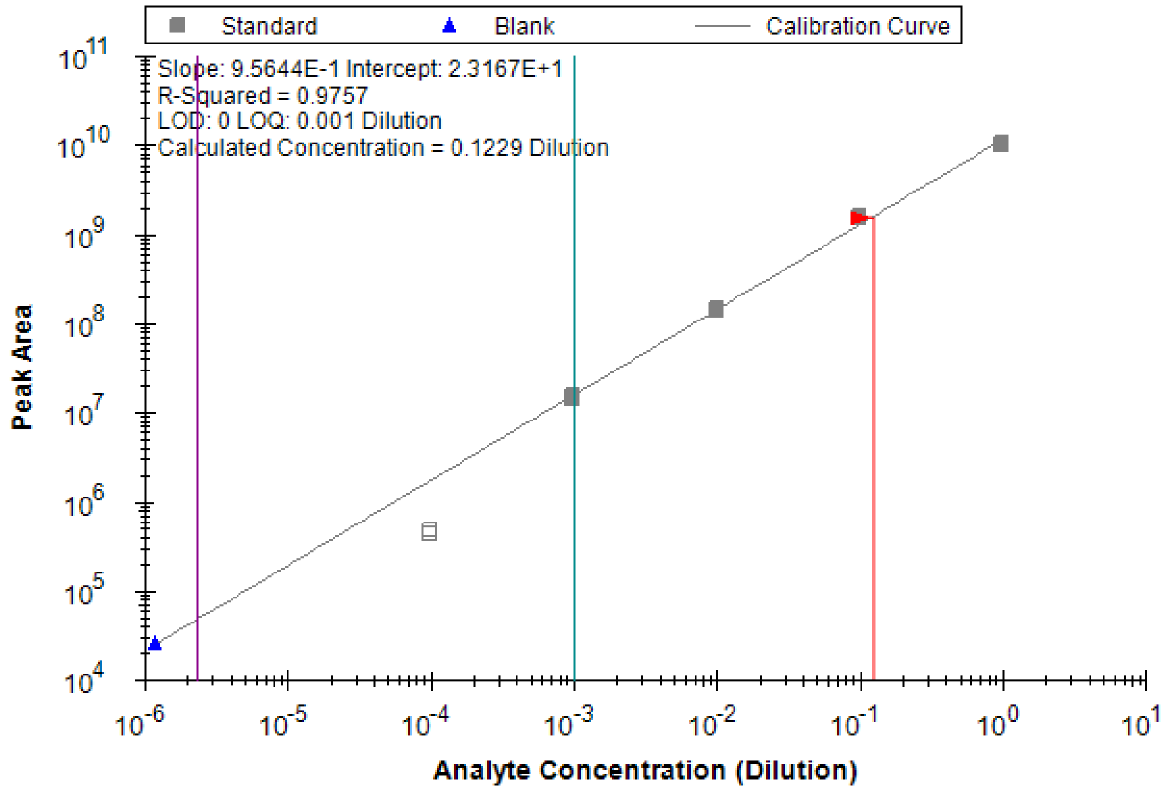

35) N1NZB8_1: ISPGDGATFPK

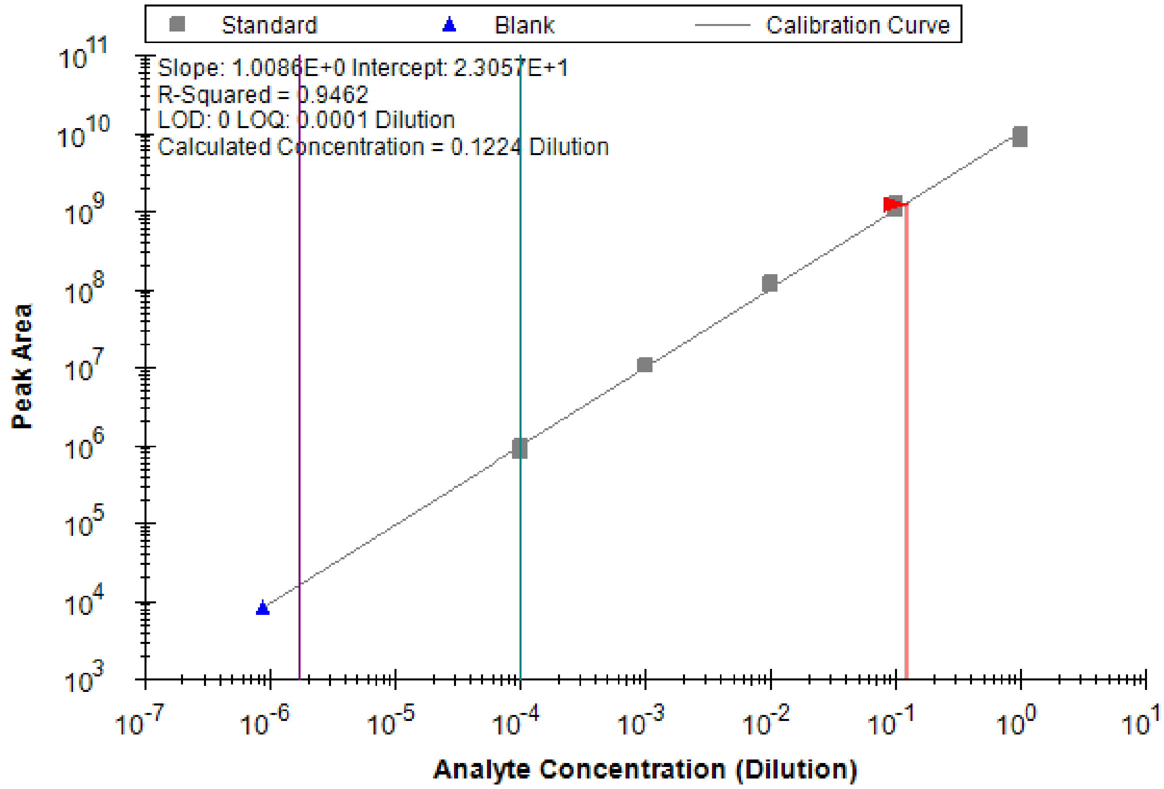

36) N1NZB8_2: FDSSVDR

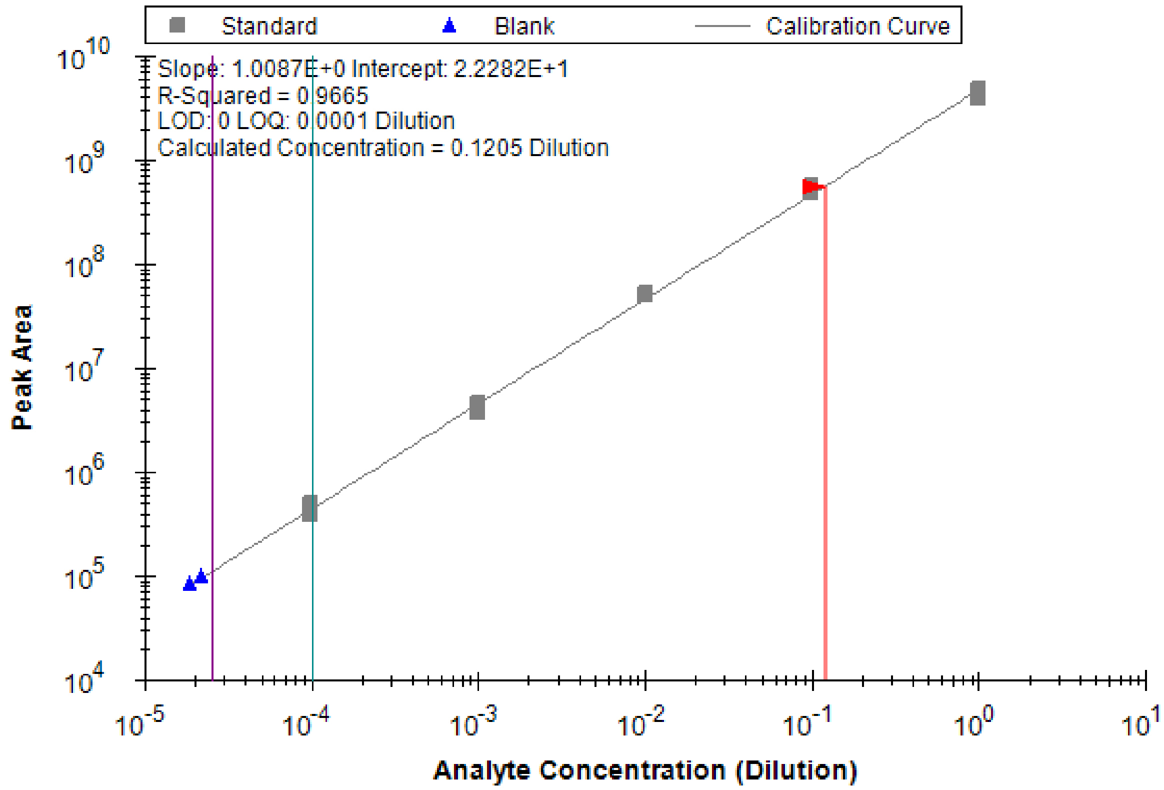

37) N1NZB8_3: GSPFQCNIGVGQVIK

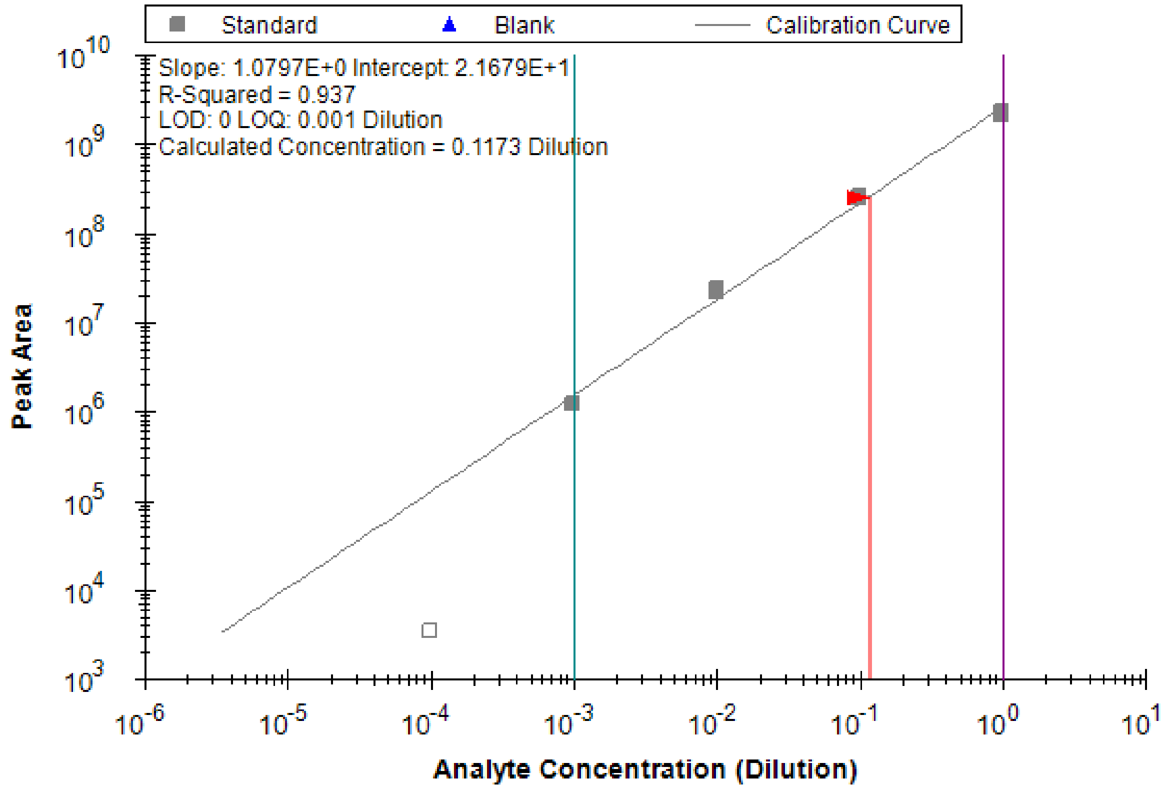

38) N1P2Y2_1: WIDLPISK

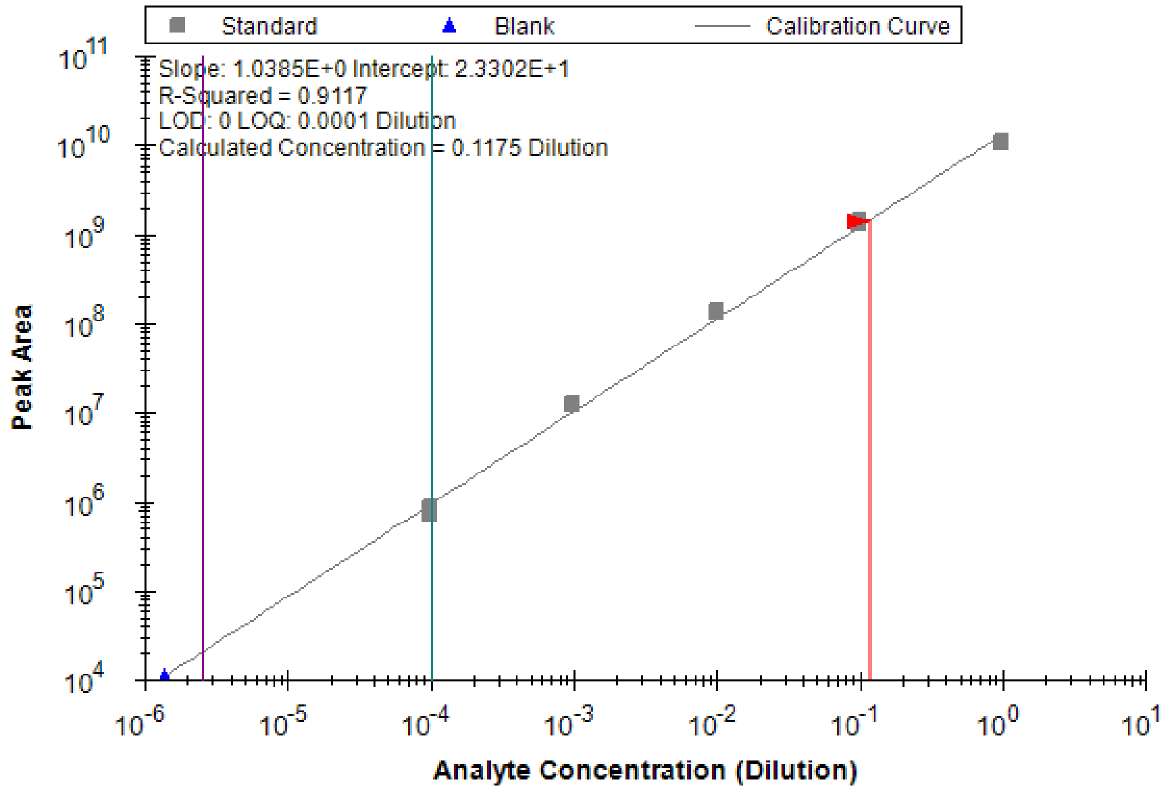

39) N1P2Y2_2: TPQDILLR

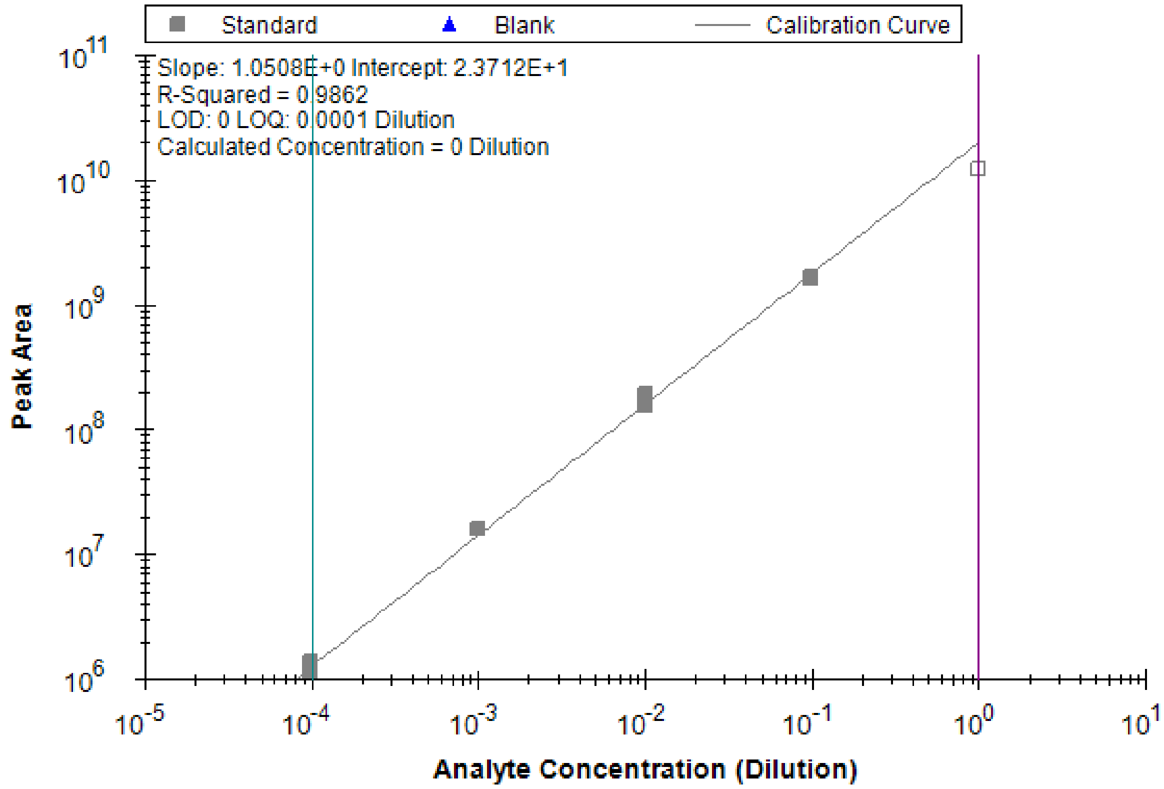

40) N1P2Y2_3: DFIQEHVPGPK

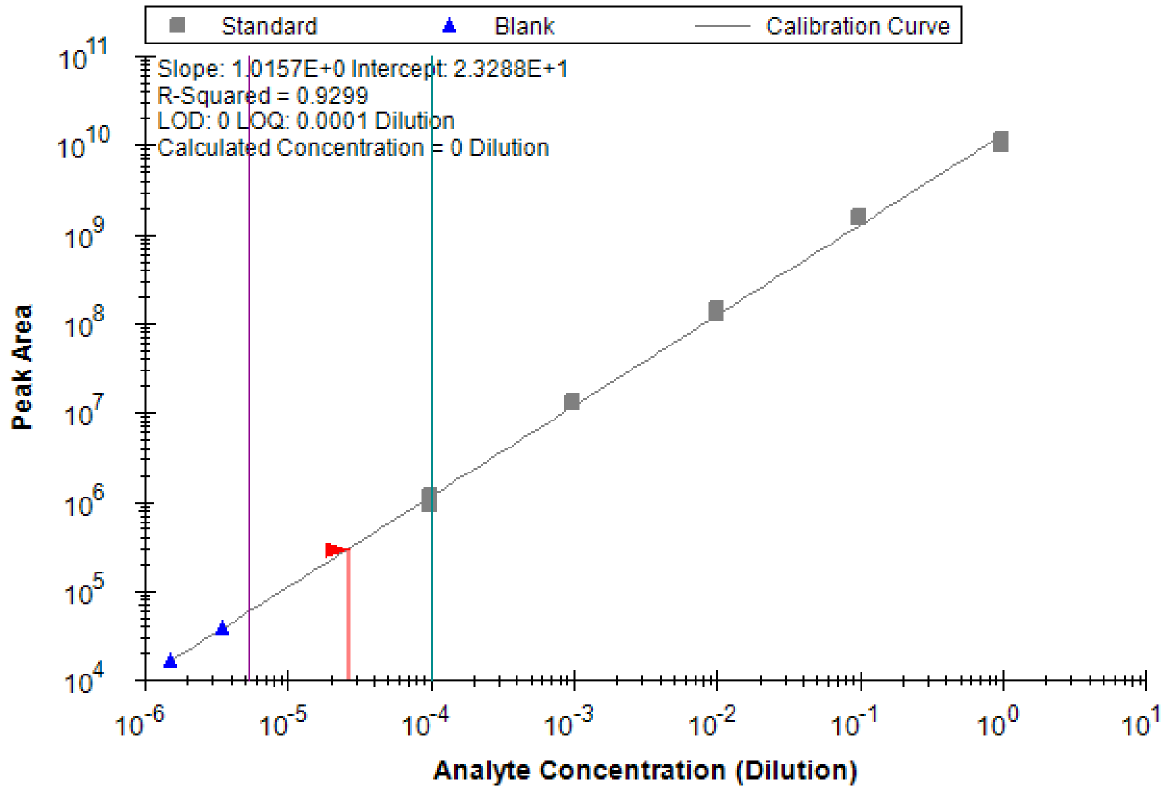

41) N1PAT2_1: ADHLVEEVLEAR

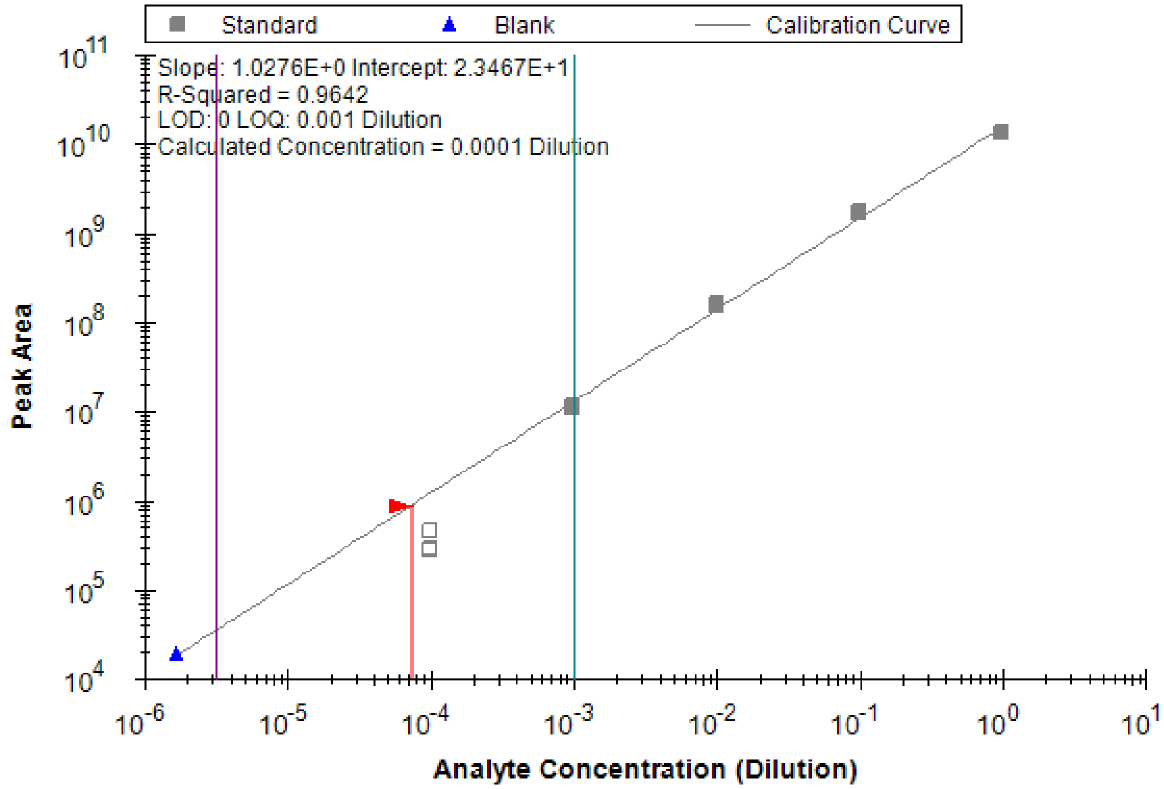

42) N1PAT2_2: GLVEDANAAAK

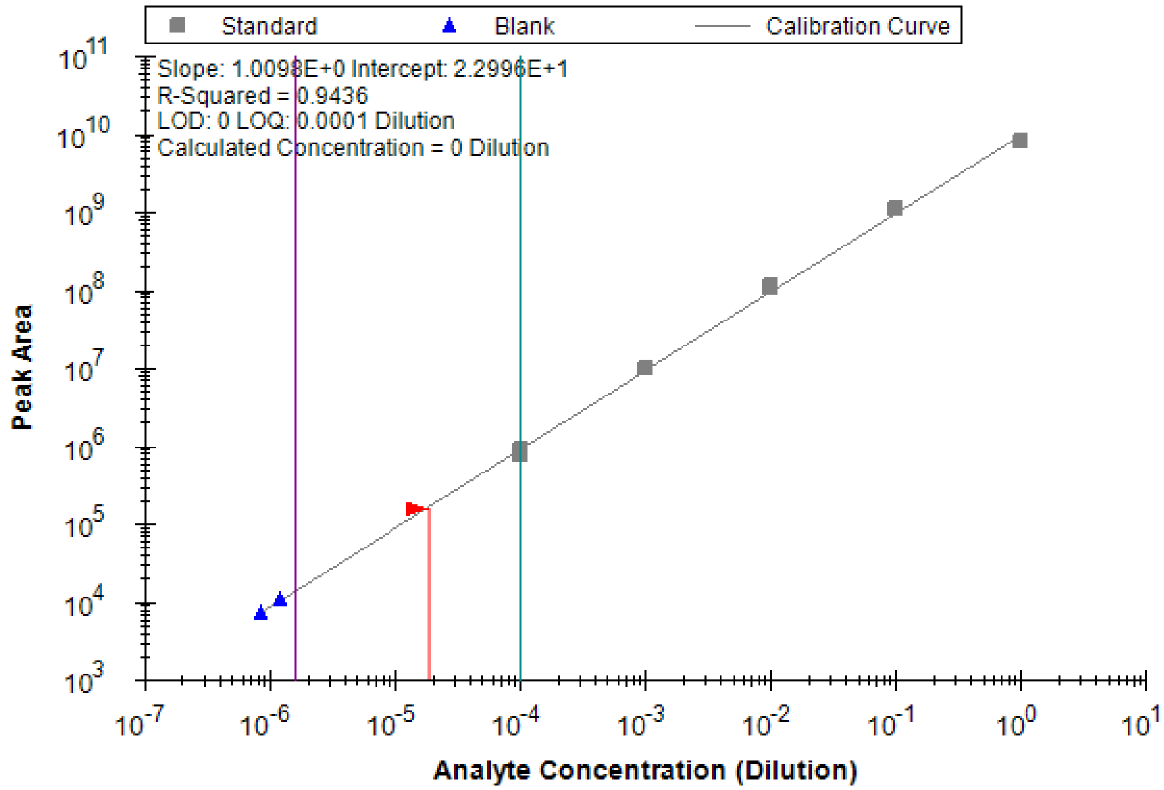

Additional File 12 of Pfister et al.

43) N1PAT2_3: YDIGNPVTGETLESPR

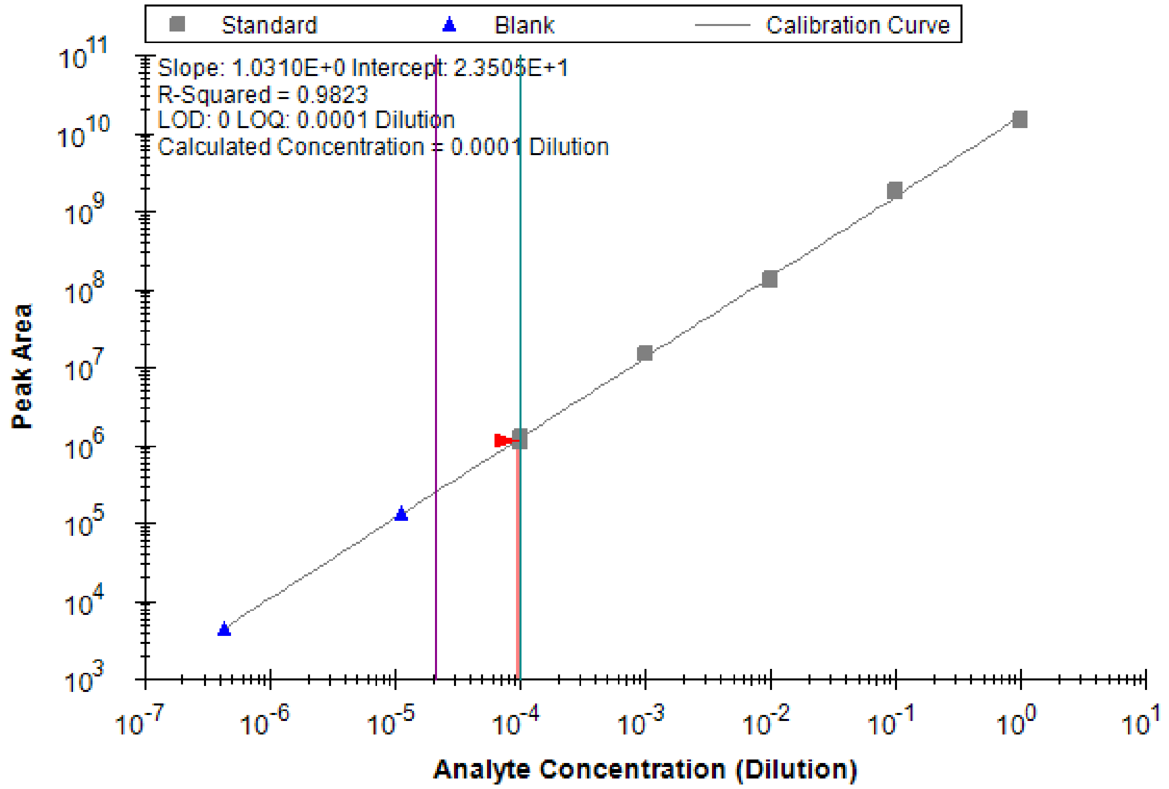

Supplement: Supplementary file 12 — Additional file 12. Targeted proteomics - Calibration curves of heavy-labelled standard peptides tested for quantification. [file 12915_2022_1408_MOESM12_ESM.pdf]
