# Supplementary material for: Tuning heterologous glucan biosynthesis in yeast to understand and exploit plant starch diversity
Source: BMC Biol. 2022 Sep 24;20:207. doi: 10.1186/s12915-022-01408-x (PMC9509603; doi:10.1186/s12915-022-01408-x)
Supplement: Supplementary file 13 — Additional file 13. Shotgun proteomics: Detailed methods. [file 12915_2022_1408_MOESM13_ESM.pdf]

Additional File 13. Detailed methods of label-free shotgun proteomics

A label-free proteomics experiment was conducted on yeast strains 29, 48A, 362.1, 363.1 and wild type (n = 4 replicates each, arising from independent replicate cultures) to compare strains with and without glucan production (**Additional file 1: Table S4**), to identify potential house-keeping proteins and to create a spectral library for subsequent peptide selection for targeted proteomics. All proteins quantified by ProgenesisQI are listed in **Additional file 14.** The MS proteomics data have been deposited to the ProteomeXchange Consortium via the PRIDE (Perez-Riverol et al., 2022) partner repository with the dataset identifier PXD032241.

For preparation of total protein extracts and tryptic peptides, yeasts were cultivated in shake flasks using YPD and YP-galactose as media in pre- and main cultures, respectively, harvested after 3 h shaking in YP-galactose, the cell pellets snap-frozen in liquid nitrogen and stored at -80°C until use as described (Pfister et al., 2016). Thawed cell pellets were homogenized in 3.3 volumes extraction buffer (100 mM Tris pH 8.2 at 4°C, complemented with protease inhibitor [Complete EDTA-free, from Roche]) and 4.3 volumes glass beads (425-600 µm diameter, acid washed) by vortexing at maximum speed for 30 min at 4°C. The homogenized cell suspension (without glass beads) was transferred to a fresh tube, supplemented with SDS to a final concentration of 4% (w/v) and boiled at 95°C for 5 min. The protein extracts were clarified by centrifugation at 16,000 *g* for 8 min at room temperature, protein concentrations in the supernatant determined by bicinchoninic acid (BCA) assay using bovine serum albumin as standard, the extracts complemented with DTT (final concentration 100 mM) and stored at -80°C. Proteins were digested by trypsin using a modified filter-aided sample preparation protocol (Wisniewski et al., 2009) on Ultracel-30 30-kDa centrifugal filters (Merck Millipore). Therefore, protein extracts were sonicated for 1 min with cooling in between, boiled for 95°C for 5 min and sonicated again. Thirty µl extracts containing 60 µg proteins were mixed with 200 µl urea buffer (8 M urea in 100 mM Tris-Cl pH 8.2) and loaded onto the centrifugal filters. Samples were further processed and desalted as described (Carrera et al., 2018), using a trypsin-to-protein ratio of 1:80 (w/w).

Mass spectrometry (MS) analysis was performed on a Q Exactive HF mass spectrometer (Thermo Fisher Scientific) equipped with a Digital PicoView source (New Objective) and coupled to an M-Class UPLC (Waters). Eluents were 0.1% (v/v) formic acid for eluent A and 0.1% (v/v) formic acid, 99.9% (v/v) acetonitrile for eluent B. Samples were loaded onto an Acquity UPLC M-class Symmetry C18 trap column (100 Å, 5 µm, 180 µm x 20 mm; Waters) coupled to an Acquity UPLC M-class HSS T3 column (1.8 µm, 75 µm x 250 mm; Waters).

Dried peptides were resuspended in 3% (v/v) acetonitrile, 0.1% (v/v) formic acid by vortexing and 5-min sonication in a water bath and supplemented with iRT peptides (Biognosys). Samples were injected in a randomized sequence without technical replication. Peptides were eluted at a flow rate of 300 nl min^{-1} using a linear 90-min gradient from 1 to 40% B. The mass spectrometer was operated in data-dependent mode, using profile as spectrum data type and acquiring full-scan MS spectra (350−1500 m/z) at a resolution of 120,000 with a target value of 3,000,000. The 12 most intense signals per cycle were isolated using an isolation window of 1.2 m/z and subjected to higher-energy collision dissociation fragmentation. MS/MS spectra were acquired at a resolution of 30,000 using a normalized collision energy of 28 and a maximum injection time of 50 ms. The automatic gain control was set to 100,000 ions. Unassigned ions and those with charge states 1 or >8 were rejected. Precursor masses previously selected for MS/MS measurement were excluded from further isolation for 30 s.

Data processing using ProgenesisQI for Proteomics (nonlinear dynamics, Waters, version 4.2.7207.22925) was conducted as described previously (Pipitone et al., 2021) but the following modifications: First, replicate 4 of strain 362.1 was used as alignment reference. Second, the Mascot generic file produced in ProgensisQI was searched against a custom forward and reverse (decoy) database (containing the CEN.PK113-7D proteome, all heterologously expressed proteins, common

MS contaminants and iRT peptide [Biognosys] sequences) with trypsin digest with up to one missed cleavages allowed, carbamidomethylation of cysteine as fixed modification, acetylation at the protein's N terminus and oxidation of methionine residue as variable modifications, a precursor ion mass tolerance of 10 ppm, and a fragment ion (MS/MS) tolerance of 10 ppm. Third, the spectrum report in Scaffold Q+S was created using a false discovery rate (FDR) of 10% and 5% at the protein and peptide level, respectively, and a minimum of one identified peptide per protein. Forth, all associated statistics (inclusive *q*-values) were directly exported from ProgenesisQI and only proteins (or protein groups) that were identified with at least two unique peptides were quantified.

References:

Carrera, D., Oddsson, S., Grossmann, J., Trachsel, C., and Streb, S. (2018). Comparative Proteomic Analysis of Plant Acclimation to Six Different Long-Term Environmental Changes. Plant Cell Physiol. **59**: 510–526.

Perez-Riverol, Y. et al. (2022). The PRIDE database resources in 2022: a hub for mass spectrometry-based proteomics evidences. Nucleic Acids Res. **50**: D543–D552.

Pfister, B., Sánchez-Ferrer, A., Diaz, A., Lu, K., Otto, C., Holler, M., Shaik, F.R., Meier, F., Mezzenga, R., and Zeeman, S.C. (2016). Recreating the synthesis of starch granules in yeast. Elife **5**: 1–29.

Pipitone, R., Eicke, S., Pfister, B., Glauser, G., Falconet, D., Uwizeye, C., Pralon, T., Zeeman, S., Kessler, F., and Demarsy, E. (2021). A multifaceted analysis reveals two distinct phases of chloroplast biogenesis during de-etiolation in Arabidopsis. Elife **10**: 1–32.

Wisniewski, J.R., Zougman, A., Nagaraj, N., and Mann, M. (2009). Universal sample preparation method for proteome analysis. Nat. Meth. **6**: 359–362.
